# A network pharmacology study on mechanism of resveratrol in treating preeclampsia *via* regulation of AGE-RAGE and HIF-1 signalling pathways

**DOI:** 10.3389/fendo.2022.1044775

**Published:** 2023-01-05

**Authors:** Jiamiao Shi, Jiahao Wang, Ning Jia, Qinru Sun

**Affiliations:** ^1^ Health Science Center, Xi'an Jiaotong University, Xi’an, Shaanxi, China; ^2^ Department of Human Anatomy, Histology and Embryology, School of Basic Medical Sciences, Health Science Center, Xi’an Jiaotong University, Xi’an, Shaanxi, China; ^3^ College of Medicine & Forensics, Health Science Center, Xi'an Jiaotong University, Xi’an, Shaanxi, China

**Keywords:** resveratrol (RSV), preeclampsia (PE), network pharmacology, bioinformatics, molecular docking

## Abstract

**Background:**

Preeclampsia (PE) is a hypertensive disorder of pregnancy that threatens the lives of millions of pregnant women and their babies worldwide. Without effective medications, there are thousands of maternal and child mortalities every year. Resveratrol (RSV), a non-flavonoid polyphenol extracted from multiple plants, has shown positive effects in treating hypertension, cardiovascular disorders, and even PE. This study aimed to explore the pharmacological mechanism of RSV in treating PE by using network pharmacology and bioinformatics.

**Methods:**

With the use of multiple databases, 66 intersecting targets were obtained from the 347 putative targets of RSV and 526 PE-related genes. Then, Gene Ontology (GO) analysis and Kyoto Encyclopedia of Genes and Genomes (KEGG) analysis were conducted to investigate the functions of the intersecting targets. The protein–protein interaction network and target–pathway network were drawn and analyzed to illustrate the correlation between targets and pathways. Finally, molecular docking was conducted to calculate the binding energy between RSV and core targets.

**Results:**

The results showed that the core targets of RSV were IL6, TNF, IL1B, VEGFA, STAT3, and EGFR. There existed good binding between RSV and IL6, TNF, IL1B, VEGFA, and EGFR. In addition, we found that RSV mainly functioned in the AGE-RAGE and HIF-1 signaling pathways, which are associated with the occurrence and development of PE.

**Conclusion:**

In conclusion, our findings indicated that RSV has the effects of regulating angiogenesis and anti-inflammation and can be a candidate medicine for treating PE.

## 1 Introduction

Preeclampsia (PE) is a multisystem disease in pregnancy, which is characterized by the onset of hypertension after 20 weeks of pregnancy, proteinuria, and malfunctions in multi-organs (1). PE now has become a leading cause of mortality and morbidity in gravidas and fetuses in many countries (2), which would undoubtedly increase the burden of social healthcare (3). In addition, PE could exert many long-term adverse influences on women such as diabetes mellitus, cerebrovascular disease, kidney disease, and even impaired memory (4). Once PE is diagnosed, delivering the babies is the only definitive treatment (2). Currently, the increased antiangiogenic factors from the placenta have been identified as the pathophysiology of PE (5). The releasing antiangiogenic factors mainly included soluble fms-like tyrosine kinase-1 (sFlt-1) and soluble endoglin (sEng). The high levels of sFlt-1 and sEng lead to dysfunction in endothelial cells and vasculature, causing damage to maternal and fetal organ systems (6). Hence, there is an urgent need for the investigation and development of effective drugs for PE patients.

Resveratrol (RSV), a natural plant polyphenol, has been reported to exert many advantageous effects on the human body including anti-oxidation (7), anti-inflammation (8), and anti-cancer (9). Recent research has just reported that RSV has potentially beneficial effects on endothelial cells incubated with PE plasma/serum (10). However, the molecular mechanism of RSV in treating PE is still unclear and needs more investigation.

Here, we conducted our work to identify the molecular mechanism of RSV in the treatment of PE. By employing bioinformatics and network pharmacology, we selected the core targets and the main signaling pathways of RSV in treating PE. Our findings verified the therapeutic effects of RSV and provided new options for the treatment of PE.

## 2 Methods and materials

### 2.1 Detection of drug likeness

The chemical and physical properties of RSV were obtained from the PubChem database (https://pubchem.ncbi.nlm.nih.gov/) (11) and SwissADME (http://www.swissadme.ch/) (12). According to Lipinski’s rule, the parameters include molecular weight (MW), topological polar surface area (TPSA), XLogP3 (octanol/water partition coefficient), counts of rotatable bonds, counts of hydrogen bond acceptor, counts of hydrogen bond donor, gastrointestinal (GI) absorption, and blood–brain barrier (BBB) permeability. A clinical medication must conform to no more than five hydrogen bond donors, no more than 10 hydrogen bond acceptors, MW less than 500, XLogP3 range between −2 and 5, and no more than 10 rotatable bonds.

### 2.2 Investigation of intersecting targets of RSV and PE

The putative targets of RSV were collected from the Traditional Chinese Medicine Systems Pharmacology Database (TCMSP) (13), SwissTargetPrediction database (14), TargetNet (15), STITCH (16), and Similarity ensemble approach (SEA) (17).

The PE-related genes were collected from the GeneCards (http://www.genecards.org/) (18) and Online Mendelian Inheritance in Man (OMIM) (https://omim.org/) (19) databases.

Then, all putative targets of RSV and PE-related genes were imported to the UniProt database for the transformation of gene symbols, and the overlapping part was called the intersecting targets of RSV and PE.

### 2.3 GO/KEGG enrichment analysis

To investigate the functions of the intersecting targets of RSV and PE, Metascape (http://metascape.org/) (20) was employed to conduct Gene Ontology (GO) analysis and Kyoto Encyclopedia of Genes and Genomes (KEGG) pathway enrichment analysis. *p*-Value <0.01 was considered as a potential pathway for all GO terms and KEGG pathways.

### 2.4 Network construction

All intersecting targets of RSV and PE were sent to the STRING database (https://string-db.org/) (21) to investigate the interaction among them, and the relationship was then exhibited by the protein–protein interaction (PPI) network. Cytoscape software (http://www.cytoscape.org/) (22) was applied for visualizing and analyzing the PPI network.

The relationship between the intersecting targets of RSV and PE and the signaling pathways from the KEGG pathway enrichment analysis was also illustrated by the target–pathway (TP) network and visualized by Cytoscape software. Finally, the core targets and the main signaling pathways were obtained.

### 2.5 Molecular docking

To further verify the physical binding capacity between the core targets and RSV, molecular docking was performed by using the AutoDockTools 1.5.6 and PyMOL software (23, 24). The 3D structure of RSV was obtained from PubChem (https://pubchem.ncbi.nlm.nih.gov/) (11), and the 3D structure of core targets was derived from Protein Data Bank (http://www.pdb.org/) (25).

## 3 Results

### 3.1 Drug likeness of RSV

To become a potential drug, a compound needs to be analyzed in accordance with Lipinski’s rule of five (RO5). The fewer violations of Lipinski’s rule there are, the greater drug likeness and pharmacokinetics a compound has. According to Lipinski’s rule, a drug-like molecule must have an MW less than 500 g/mol, an octanol/water partition coefficient (XLogP3) less than 5, no more than 10 hydrogen bond acceptors, no more than 5 hydrogen bond donors, and rotatable bonds less than 10.

The parameters of RSV from the PubChem and SwissADME databases are listed in **Table 1**. The results showed that RSV was in line with Lipinski’s rule, which suggests that RSV possesses pharmaceutical properties. The results also indicated good GI absorption and BBB penetration of RSV.

**Table 1:** The molecular parameters of RSV.

Property	Parameter
MW	228.24 g/mol
TPSA	60.69 Å
XLogP3	3.1
Rotatable bonds	2
H-bond acceptors	3
H-bond donors	3
GI absorption	High
BBB permeability	Yes
Lipinski’s rule	0 violations

MW, molecular with; TPSA, topological polar surface area; XLogP3, octanol/water partition coefficient; H-bond, hydrogen bond; GI, gastrointestinal; BBB, blood–brain barrier; RSV, resveratrol.

### 3.2 The putative targets of RSV and PE-related genes

To collect the putative targets of RSV, five databases were applied including TCMSP, TargetNet, SwissTargetPrediction, STITCH, and SEA. As a result, a total of 347 proteins were collected from the databases mentioned above after deleting the duplicate targets. These targets were listed in **Supplementary Material Additional file 1**.

The PE-related genes were collected from the GeneCards database and OMIM database. After the redundant information was deleted, a total of 511 genes were identified and listed in **Supplementary Material Additional file 2**.

**Figure 1:**
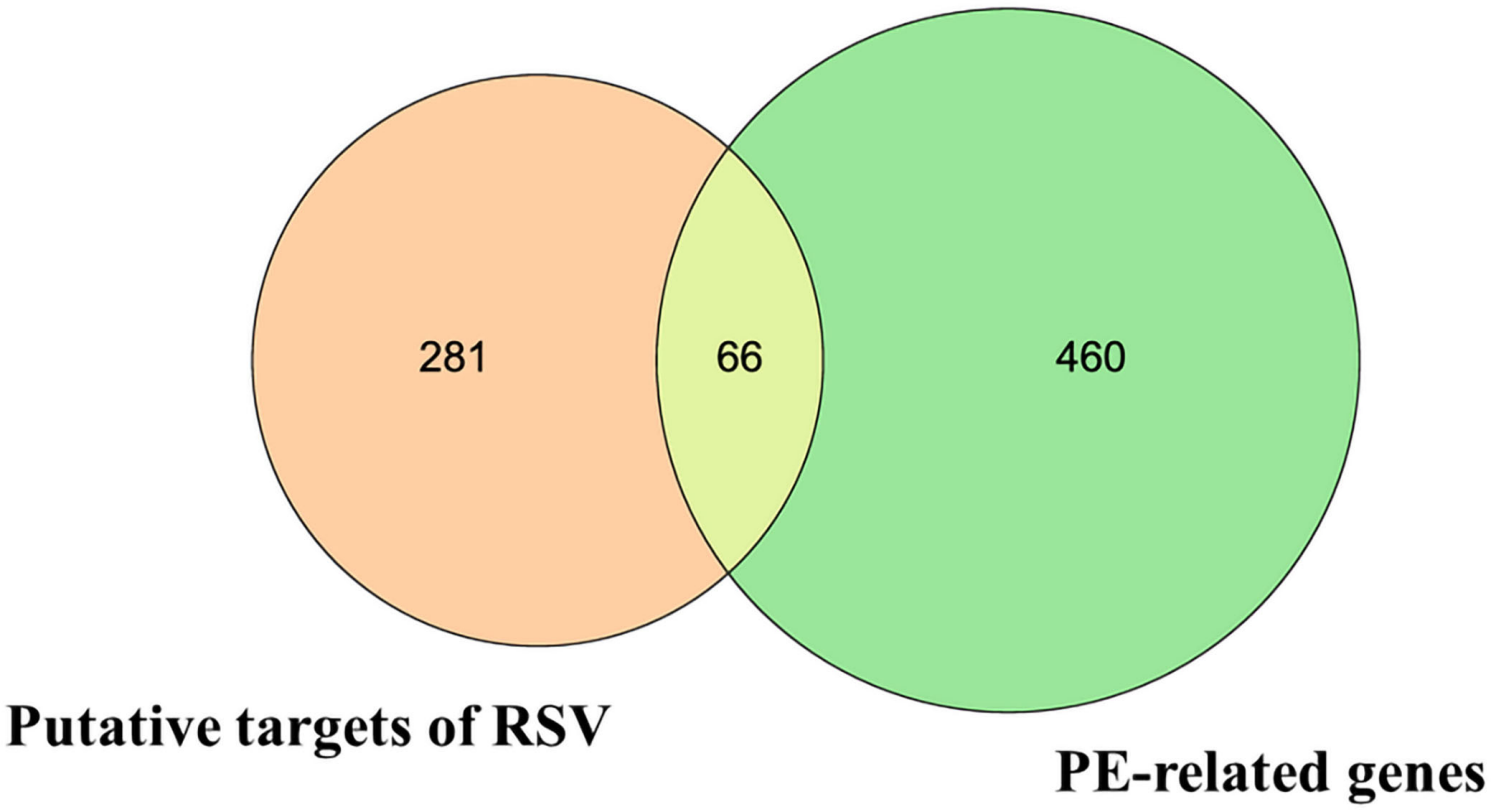
The Venn diagram. The orange circle represents the putative targets of RSV. The green circle represents the PE-related genes. The yellow overlapping part represents the 66 intersecting targets. RSV, resveratrol; PE, preeclampsia.

Then, the 66 overlapping targets of these two lists formed the intersecting targets of RSV and PE (**Figure 1**) (**Supplementary Material Additional file 3**).

### 3.3 Exploration of intersecting targets of RSV and PE

To further identify the functions of the intersecting targets of RSV and PE, GO analysis and KEGG analysis were carried out. A total of 1,707 GO terms and 125 KEGG pathways were identified (**Supplementary Material Additional file 4** and **Additional file 5**). Among the GO terms, 1,582 were for biological processes (BPs), 52 were for cellular components (CCs), and 73 were for molecular functions (MFs).To illustrate more explicitly the results of GO and KEGG analyses, bubble grams were applied.

**Figure 2:**
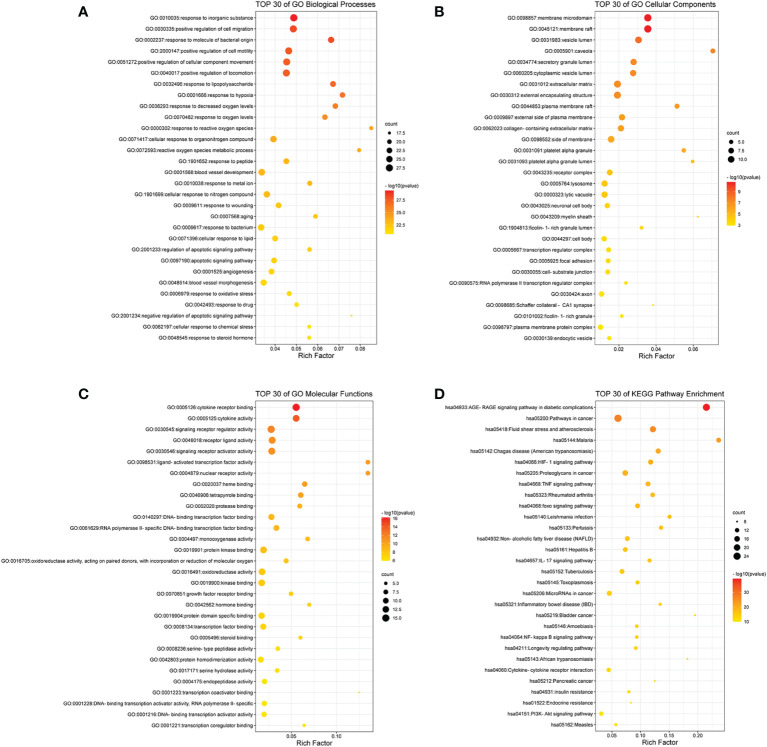
The bubble gram of the results of GO and KEGG pathway enrichment analyses. **(A)** The top 30 GO terms of biological processes. **(B)** The top 30 GO terms of cellular components. **(C)** The top 30 GO terms of molecular functions. **(D)** The top 30 KEGG enrichment pathways. All the GO terms and KEGG pathways with *p*-value <0.01 were considered to be statistically significant. GO, Gene Ontology; KEGG, Kyoto Encyclopedia of Genes and Genomes.

As shown in **Figure 2A**, the top 30 of BP terms were response to inorganic substance (GO:0010035), positive regulation of cell migration (GO:0030335), response to molecule of bacterial origin (GO:0002237), positive regulation of cell motility (GO:2000147), positive regulation of cellular component movement (GO:0051272), positive regulation of locomotion (GO:0040017), response to lipopolysaccharide (GO:0032496), response to hypoxia (GO:0001666), response to decreased oxygen levels (GO:0036293), response to oxygen levels (GO:0070482), response to reactive oxygen species (GO:0000302), cellular response to organonitrogen compound (GO:0071417), reactive oxygen species metabolic process (GO:0072593), response to peptide (GO:1901652), blood vessel development (GO:0001568), response to metal ion (GO:0010038), cellular response to nitrogen compound (GO:1901699), response to wounding (GO:0009611), aging (GO:0007568), response to bacterium (GO:0009617), cellular response to lipid (GO:0071396), regulation of apoptotic signaling pathway (GO:2001233), apoptotic signaling pathway (GO:0097190), angiogenesis (GO:0001525), blood vessel morphogenesis (GO:0048514), response to oxidative stress (GO:0006979), response to drug (GO:0042493), negative regulation of apoptotic signaling pathway (GO:2001234), cellular response to chemical stress (GO:0062197), and response to steroid hormone (GO:0048545).

As shown in **Figure 2B**, the top 30 CC terms were membrane raft (GO:0045121), membrane microdomain (GO:0098857), vesicle lumen (GO:0031983), caveola (GO:0005901), secretory granule lumen (GO:0034774), cytoplasmic vesicle lumen (GO:0060205), extracellular matrix (GO:0031012), external encapsulating structure (GO:0030312), plasma membrane raft (GO:0044853), external side of plasma membrane (GO:0009897), collagen-containing extracellular matrix (GO:0062023), side of membrane (GO:0098552), platelet alpha granule (GO:0031091), platelet alpha granule lumen (GO:0031093), receptor complex (GO:0043235), lytic vacuole (GO:0000323), lysosome (GO:0005764), neuronal cell body (GO:0043025), myelin sheath (GO:0043209), ficolin-1-rich granule lumen (GO:1904813), cell body (GO:0044297), transcription regulator complex (GO:0005667), focal adhesion (GO:0005925), cell–substrate junction (GO:0030055), RNA polymerase II transcription regulator complex (GO:0090575), axon (GO:0030424), Schaffer collateral–CA1 synapse (GO:0098685), ficolin-1-rich granule (GO:0101002), plasma membrane protein complex (GO:0098797), and endocytic vesicle (GO:0030139).

As shown in **Figure 2C**, the top 30 MF terms were cytokine receptor binding (GO:0005126), cytokine activity (GO:0005125), signaling receptor regulator activity (GO:0030545), receptor ligand activity (GO:0048018), signaling receptor activator activity (GO:0030546), nuclear receptor activity (GO:0004879), ligand-activated transcription factor activity (GO:0098531), heme binding (GO:0020037), tetrapyrrole binding (GO:0046906), protease binding (GO:0002020), DNA-binding transcription factor binding (GO:0140297), RNA polymerase II-specific DNA-binding transcription factor binding (GO:0061629), monooxygenase activity (GO:0004497), protein kinase binding (GO:0019901), oxidoreductase activity, acting on paired donors, with incorporation or reduction of molecular oxygen (GO:0016705), oxidoreductase activity (GO:0016491), kinase binding (GO:0019900), growth factor receptor binding (GO:0070851), hormone binding (GO:0042562), protein domain specific binding (GO:0019904), transcription factor binding (GO:0008134), steroid binding (GO:0005496), serine-type peptidase activity (GO:0008236), protein homodimerization activity (GO:0042803), serine hydrolase activity (GO:0017171), endopeptidase activity (GO:0004175), transcription coactivator binding (GO:0001223), DNA-binding transcription activator activity, RNA polymerase II-specific (GO:0001228), DNA-binding transcription activator activity (GO:0001216), and transcription coregulator binding (GO:0001221).

As shown in **Figure 2D**, the top 30 KEGG pathways were AGE-RAGE signaling pathway in diabetic complications (hsa04933), pathways in cancer (hsa05200), fluid shear stress and atherosclerosis (hsa05418), malaria (hsa05144), Chagas disease (American trypanosomiasis) (hsa05142), HIF-1 signaling pathway (hsa04066), proteoglycans in cancer (hsa05205), TNF signaling pathway (hsa04668), rheumatoid arthritis (hsa05323), foxo signaling pathway (hsa04068), *Leishmania* infection (hsa05140), pertussis (hsa05133), non-alcoholic fatty liver disease (NAFLD) (hsa04932), hepatitis B (hsa05161), IL17 signaling pathway (hsa04657), tuberculosis (hsa05152), toxoplasmosis (hsa05145), microRNAs in cancer (hsa05206), inflammatory bowel disease (IBD) (hsa05321), bladder cancer (hsa05219), NF-kappa B signaling pathway (hsa04064), amebiasis (hsa05146), longevity regulating pathway (hsa04211), African trypanosomiasis (hsa05143), cytokine–cytokine receptor interaction (hsa04060), pancreatic cancer (hsa05212), insulin resistance (hsa04931), endocrine resistance (hsa01522), PI3K-Akt signaling pathway (hsa04151), and measles (hsa05162).

### 3.4 Analyses of PPI network and TP network

**Figure 3:**
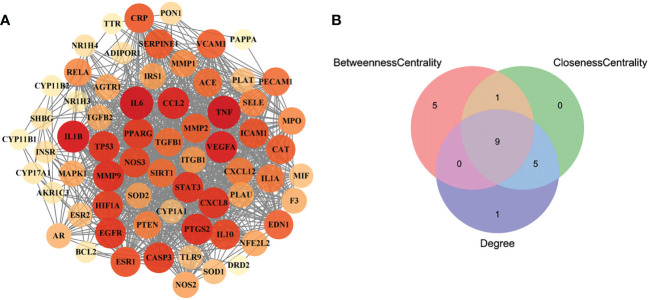
The PPI network of the intersecting targets of RSV and PE and the topological parameters analysis of the PPI network. **(A)** The PPI network of the intersecting targets of RSV and PE. **(B)** The Venn diagram of the top 15 proteins of each topological parameter. Nine proteins located in the central region were identified as the hub targets. The red circle represents the top 15 proteins of betweenness centrality, the green circle represents the top 15 proteins of closeness centrality, and the blue circle represents the top 15 proteins of degree. PPI, protein–protein interaction; RSV, resveratrol; PE, preeclampsia.

In order to obtain the interaction relationship among the intersecting targets, the STRING database was employed. All 66 targets were imported into the STRING database to illustrate the PPI network. Then, the PPI network was modified by using the Cytoscape software (**Figure 3A**). The proteins of the PPI network were evaluated and analyzed using topological parameters, including degree, betweenness centrality, and closeness centrality. The results of the PPI network are shown in **Supplementary Material Additional file 6**. After topological parameters of the proteins of the PPI network were evaluated and the intersection of the top 15 targets of each parameter was obtained, IL6, TNF, EGFR, PPAR, IL1B, PTGS2, VEGFA, CCL2, and STAT3 were identified as the nine hub targets of PPI network (**Figure 3B**).

**Figure 4:**
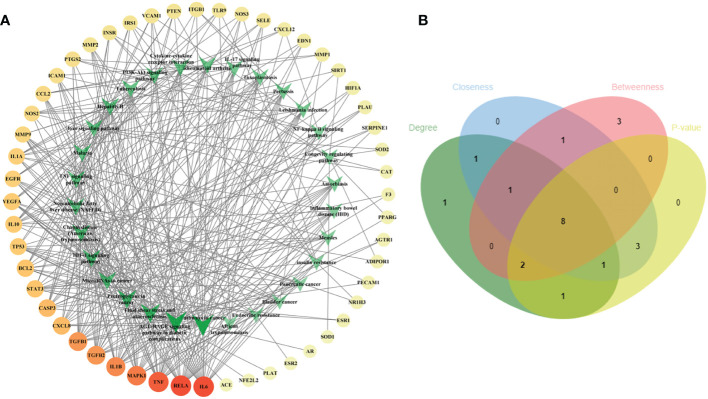
The TP network between the 52 intersecting targets of RSV and PE and the top 30 pathways in the KEGG analysis and the topological parameters analysis of the TP network. **(A)** The TP network between the 52 intersecting targets of RSV and PE and the top 30 pathways in the KEGG analysis. The different colors of circular codes in the outer circle represent the targets. From yellow to red, the score gradually becomes higher. The green V-shaped nodes in the inner circle represent the pathways. The deeper the green node is, the higher its score. **(B)** The Venn diagram of the top 15 pathways of each topological parameter. Eight pathways located in the central region were the main pathways. The green ellipse represents degree. The blue ellipse represents closeness centrality. The red ellipse represents betweenness centrality. The yellow ellipse represents the first 15 pathways with the least *p*-value. Eight pathways located in the central region were identified as the main pathways. TP, target–pathway; RSV, resveratrol; PE, preeclampsia; KEGG, Kyoto Encyclopedia of Genes and Genomes.

To illustrate the correlation between the 52 intersecting targets of RSV and PE and the top 30 related pathways in the KEGG analysis, the TP network was constructed (**Figure 4A**). The result showed that in the top 30 pathways, there were 16 hub proteins including IL6, RELA, TNF, MAPK1, IL1B, TGFB1, TGFB2, CASP3, CXCL8, BCL2, STAT3, VEGFA, TP53, IL10, IL1A, and EGFR in the TP network for their high frequency (>10 times) (**Supplementary Material Additional file 7**). Finally, the intersection of the hub targets of the TP network and hub targets of the PPI network was obtained, and six overlapping targets between the two groups of hub targets were considered as the core targets of RSV in the treatment of PE. These core targets were IL6, TNF, IL1B, VEGFA, STAT3, and EGFR.

Furthermore, the main pathways from the top 15 pathways of the result of KEGG pathway enrichment analysis were screened according to the score of three topological parameters and *p*-value obtained from the Metascape database (**Figure 4B**). The results showed that eight pathways were identified as the main pathways, including pathways in cancer, fluid shear stress and atherosclerosis, AGE-RAGE signaling pathway in diabetic complications, proteoglycans in cancer, NAFLD, Chagas disease (American trypanosomiasis), HIF-1 signaling pathway, and TNF signaling pathway.

**Figure 5:**
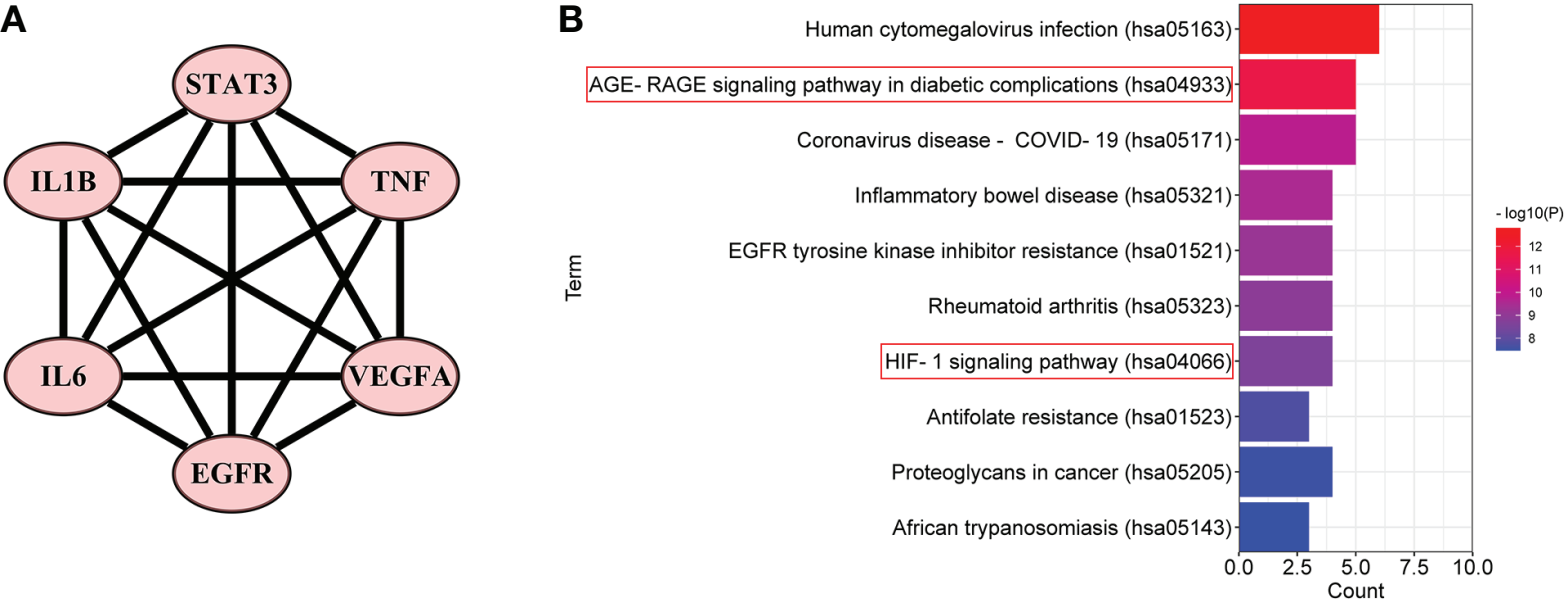
The PPI network and the top 10 related pathways of the six core targets. **(A)** The PPI network of the six core targets. **(B)** The top 10 signaling pathways related to the six core targets. PPI, protein–protein interaction.

Furthermore, in order to elaborate on the correlation between the six core targets, the STRING database was used to construct their PPI network. As shown in **Figure 5A**, these six core targets formed a regular hexagon, which indicated that there is a close relationship between them. In addition, the Metascape database was used to conduct a KEGG pathway enrichment analysis to select the pathways related to the six core targets. As shown in **Figure 5B**, the top 10 pathways included human cytomegalovirus infection (hsa05163), AGE-RAGE signaling pathway in diabetic complications (hsa04933), coronavirus disease–COVID-19 (hsa05171), inflammatory bowel disease (hsa05321), EGFR tyrosine kinase inhibitor resistance (hsa01521), rheumatoid arthritis (hsa05323), HIF-1 signaling pathway (hsa04066), antifolate resistance (hsa01523), proteoglycans in cancer (hsa05205), and African trypanosomiasis (hsa05143). Comparing the above-obtained eight pathways from the TP network, we found that there were four overlapping pathways between them, including the AGE-RAGE signaling pathway in diabetic complications (hsa04933), HIF-1 signaling pathway (hsa04066), proteoglycans in cancer (hsa05205), and African trypanosomiasis (hsa05143). Eventually, by synthetically analyzing the gene count and adjusted *p*-value, two signaling pathways, the AGE-RAGE signaling pathway in diabetic complications (hsa04933) and the HIF-1 signaling pathway (hsa04066), were chosen to be the potential signaling pathways of RSV in the treatment of PE.

### 3.5 Molecular docking

To further investigate the physical binding between the core targets and RSV, molecular docking was carried out and visualized. The values of binding energy are shown in **Table 2**. It is obvious that the values of binding energy between the core targets and RSV were lower than −5 kcal/mol except for STAT3, which indicated that there was good binding between them.

**Table 2:** The binding energy of RSV and the core targets.

Binding energy (kcal/mol)	IL6	TNF	IL1B	VEGFA	STAT3	EGFR
RSV	−5.53	−5.2	−6.15	−5.93	−4.89	−5.19

RSV, resveratrol.

**Figure 6:**
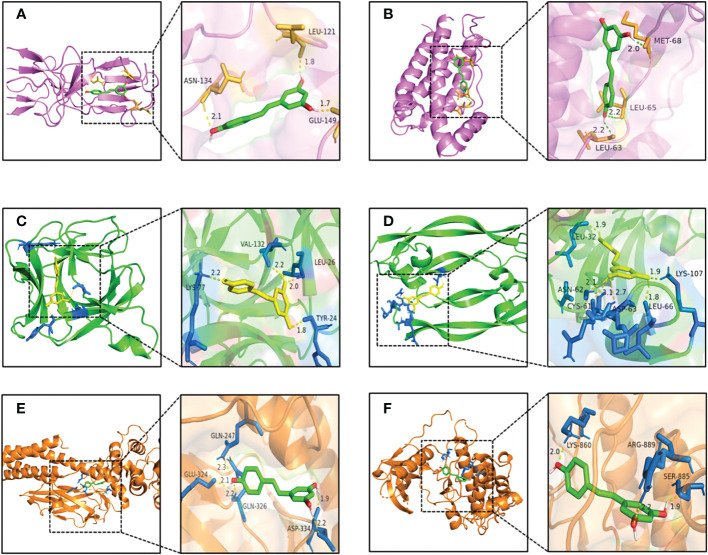
The pattern diagrams of molecular docking of RSV and core targets. **(A)** RSV and TNF, **(B)** RSV and IL6, **(C)** RSV and IL1B, **(D)** RSV and VEGFA, **(E)** RSV and STAT3, and **(F)** RSV and EGFR. The decimals mark the length of hydrogen bonds. RSV, resveratrol.

As shown in **Figure 6A**, RSV was embedded in the pocket formed by the active sites of ASN-134, LEU-121, and GLU-149, and there existed hydrogen bonds varying within a range of 1.7 to 2.1 Å between RSV and the active sites of TNF. As shown in **Figure 6B**, the active sites of IL6 including MET-68, LEU-65, and LEU-63 interplayed with RSV by several shorter hydrogen bonds. **Figure 6C** shows that there was a binding between RSV and IL1B at the active sites of LYS-77, VAL-132, LEU-26, and TYR-24. The interaction of VEGFA and RSV is shown in **Figure 6D**, and the active sites were CYS-61, ASN-62, ASP-63, LEU-32, LYS-107, and LEU-66. As shown in **Figure 6E**, RSV bonded with STAT3 in the pocket made by four active sites including GLN-247, GLU-324, GLN-326, and ASP-334. **Figure 6F** shows that EGFR interacted with RSV in the active sites of LYS-860, ARG-889, and SER-885.

## 4 Discussion

PE has become the major cause of maternal and fetal death worldwide. Furthermore, during their lifetime, women with preeclampsia are also more likely to suffer from cardiovascular disorders than those without. Unfortunately, there is still no effective treatment except termination of pregnancy. There is an urgent need to seek new drug targets and develop new effective therapies. RSV is a native polyphenol extracted from many plants. It has been reported to exert anti-oxidation, anti-inflammation, and anti-cancer effects. The positive influences on complicated pregnancy and a decrease in cardiovascular events both have been verified (26, 27).

As a good source of RSV, the Mediterranean diet is rich in many phenols. As the main components of the Mediterranean diet, red wine is rich in RSV, and olive oil can increase the absorption of RSV (28, 29). The Mediterranean diet can modulate inflammatory factors such as TNF and IL6, which are consistent with the finding of core targets of RSV (30) (31). Recent studies have shown that the Mediterranean diet can significantly alleviate gestational diabetes mellitus (GDM) and insulin resistance, which could be induced by obesity (32, 33). Women with GDM have an increased risk of PE due to insulin resistance-induced hyperinsulinemia (34). A cohort study showed that the Mediterranean diet alleviated symptoms of PE (35). Therefore, we speculate that there is a curious coincidence between the Mediterranean diet and the administration of RSV, and RSV might be the main active ingredient of the Mediterranean diet.

PE is a complicated disease that affects multiple cells, organs, or systems including the placenta, trophoblast cells, and endothelial cells. Previous studies have shown that RSV upregulates VEGF (36) and inhibits the release of sFlt-1 from the placenta (37, 38). In addition, RSV could protect the trophoblast against oxidative stress (39, 40) and improve vascular endothelial functions (41–43). A clinical trial showed that RSV is a safe and effective adjuvant of oral nifedipine to attenuate hypertensive symptoms among PE patients (44). A meta-analysis showed that the combined treatment of RSV and nifedipine could take less time to achieve the target blood pressure than nifedipine alone, and less nifedipine dose was required in the combined treatment (45). However, the molecular mechanism of RSV against PE remains unclear.

In this study, we investigated the potential of RSV as a candidate medicine for the treatment of PE. There were 66 intersecting targets of RSV and PE between the 347 potential targets of RSV and the 526 PE-related genes. The results of GO showed that more than 38 GO terms widely participated in the pathophysiology of PE. These GO terms included pregnancy, regulation of blood pressure, positive regulation of angiogenesis, inflammatory factors, endothelial cell proliferation and migration, and oxidative stress. Previous studies have shown that these processes are involved in the pathological processes of PE. Specifically, PE is a main complication of pregnancy, manifested as dysregulation of blood pressure (46). The decrease of angiogenesis in the uterine led to maternal blood pressure, and it impaired placental and fetal development as well (47). Some researchers have theorized that maternal endothelial dysfunction may be a biomarker for PE (48). The excessive activation of the immune system and increase of proinflammatory cytokines are considered manifestations of PE (49). Notably, it has been generally accepted that excessive production of reactive oxygen species (ROS) and antioxidant system dysfunction are involved in the etiology of PE (50). Taken together, we reasonably assume that RSV has therapeutic effects on PE through regulation of blood pressure, participation in circulation and vasculature, modulation of cytokines and hormones, control of epithelial cells, and response to oxidative stress and inflammation.

In order to further obtain the core targets among the intersecting targets, the PPI network between the intersecting targets and the TP network between the intersecting targets and the signaling pathways were constructed and analyzed. Eventually, six core targets, namely, TNF, IL6, IL1B, VEGFA, STAT3, and EGFR, were required.

TNF, as a pro-inflammatory cytokine, functions to a great extent in PE. The increase in TNF has been verified in the preclinical models of PE (51). There have been considerable studies to support that the level of TNF can be used as a biomarker for preeclampsia diagnosis (52). The serum levels of IL6, an important pro-inflammatory cytokine, were abnormal in women with PE (53). IL1B, as a pro-inflammatory cytokine, has been considered to be involved in the pathogenesis of PE *via* activation of NOD-like receptor protein 3 (NLRP3) inflammasome (54, 55). VEGFA is proposed to play roles in angiogenesis and vasculature in the human body. The decrease of VEGFA in cerebrospinal fluid protein in PE has been detected (56). Downregulation of VEGFA may contribute to the development of PE (57). Recently, EGFR has attracted increasing attention due to its involvement in the mechanism of aspirin in the treatment of PE (58). A recent study has also demonstrated that the EGFR signaling regulated the secretion of sFlt-1, which has been strongly implicated in the pathogenesis of PE (59). Additionally, the downregulation of EGFR-related pathways might account for impaired trophoblast invasion in PE (60). In a word, the core targets that we obtained are engaged in the pathophysiology of PE and may be the therapeutic targets of RSV against PE. Signal transducer and activator of transcription 3 (STAT3), a latent cytoplasmic transcription factor involved in endothelial cell differentiation, survival, and angiogenesis, may play an important role in preeclampsia-associated endothelial dysfunction (61). Several studies have verified the efficacy of RSV in the treatment of PE through core targets that we found. RSV could improve the expression of VEGFA in PE (36) and reduce the secretion of IL6, TNF, and IL1B (37). In addition, RSV could decrease the level of EGFR and reduce EGFR and STAT3 activation (62). Of course, more studies are needed to verify the pharmacological mechanism of RSV in the treatment of PE.

Furthermore, we conducted molecular docking to validate the relationship between RSV and six core targets of PE. The results showed that they all had good binding with RSV except STAT3. However, a recent study has demonstrated that the IL6/Jak2/STAT3 signaling pathway is involved in the development of PE (63). These results finally indicated that these six targets might be the targets of RSV against PE, and more experimental and clinical studies are still needed in the future.

By constructing the TP network, among a total of 125 pathways from KEGG pathway enrichment analysis, eight pathways related to the intersecting targets of RSV and PE were obtained including pathways in cancer, fluid shear stress and atherosclerosis, AGE-RAGE signaling pathway in diabetic complications, proteoglycans in cancer, NAFLD, Chagas disease (American trypanosomiasis), HIF-1 signaling pathway, and TNF signaling pathway. Further, the interaction between these eight pathways and the signaling pathways related to the six core targets were obtained, and two pathways, the AGE-RAGE signaling pathway in diabetic complications and the HIF-1 signaling pathway, were finally acquired.

An abnormal elevation of AGE/RAGE in PE patients *via* possible involvement of placental oxidative and nitrative stress has been reported (64). An enhanced expression of AGE/RAGE may be the biomarker for the diagnosis and prognosis of PE patients (65). The AGE-RAGE signaling pathway may also participate in the occurrence of GDM (66, 67), which probably shares some common pathophysiological pathways (68). Hypoxia-inducible factor (HIF) is a transcription factor that can be activated by hypoxia. Placental hypoxia may be one of the major pathogenesis of PE, which can lead to the accumulation of HIF (69). The upregulation of HIF may damage the angiogenesis of the placenta (70), suppress the invasion and migration of trophoblastic cells (71), and contribute to the development of PE (72). In addition to the above, some researchers proposed that the downregulation of HIF might be an efficient therapy for PE (73, 74). Previous studies have shown that the HIF-1 signaling pathway is involved in the pathogenesis of PE (70, 74–78). There are two main subtypes of PE, early-onset PE and late-onset PE. Early-onset PE is considered to be related to specific trophoblast defects, and late-onset PE is related to maternal metabolic defects (79). However, HIF-1 and AGE-RAGE signaling pathways are considered to be common pathways involved in the pathogenesis of early- and late-onset PE (80). Further experimental and clinical studies in developing therapies for PE targeting these pathways remain necessary in the future.

## Data availability statement

The datasets presented in this study can be found in online repositories. The names of the repository/repositories and accession number(s) can be found in the article/**Supplementary Material**.

## Author contributions

Conceptualization: QS and NJ. Investigation: JS and JW. Methodology: JS and JW. Supervision: QS and NJ. Visualization: JS and JW. Writing—original draft: QS, JS, and JW. Writing—review and editing: QS and NJ. All authors contributed to the article and approved the submitted version.
