# Supplementary material for: A network pharmacology study on mechanism of resveratrol in treating preeclampsia via regulation of AGE-RAGE and HIF-1 signalling pathways
Source: Front Endocrinol (Lausanne). 2023 Jan 5;13:1044775. doi: 10.3389/fendo.2022.1044775 (PMC9849370; doi:10.3389/fendo.2022.1044775)
Supplement: Supplementary file 1 [file Table_1.docx]

**Additional file 1. The putative targets of RSV**

| **No.** | **Target Name** | **Gene Symbol** |
| --- | --- | --- |
| 1 | Alpha- and gamma-adaptin-binding protein p34 | AAGAB |
| 2 | 4-aminobutyrate aminotransferase, mitochondrial | ABAT |
| 3 | ATP-dependent translocase ABCB1 | ABCB1 |
| 4 | Multidrug resistance protein 1 | ABCB1 |
| 5 | Multidrug resistance-associated protein 1 | ABCC1 |
| 6 | ATP-binding cassette sub-family C member 2 | ABCC2 |
| 7 | ATP-binding cassette sub-family G member 1 | ABCG1 |
| 8 | ATP-binding cassette sub-family G member 2 | ABCG2 |
| 9 | Angiotensin-converting enzyme | ACE |
| 10 | Acetylcholinesterase | ACHE |
| 11 | Adiponectin receptor protein 1 | ADIPOR1 |
| 12 | Adiponectin receptor protein 2 | ADIPOR2 |
| 13 | Activity-dependent neuroprotector homeobox protein | ADNP |
| 14 | Adenosine receptor A2a | ADORA2A |
| 15 | Alpha-2A adrenergic receptor | ADRA2A |
| 16 | Alpha-2C adrenergic receptor | ADRA2C |
| 17 | Type-1 angiotensin II receptor | AGTR1 |
| 18 | Aryl hydrocarbon receptor | AHR |
| 19 | Activator of 90 kDa heat shock protein ATPase homolog 1 | AHSA1 |
| 20 | Aldo-keto reductase family 1 member B1 | Akr1b1 |
| 21 | Aldo-keto reductase family 1 member B10 | AKR1B10 |
| 22 | Aldo-keto reductase family 1 member C3 | AKR1C3 |
| 23 | RAC-alpha serine/threonine-protein kinase | AKT1 |
| 24 | Succinate-semialdehyde dehydrogenase, mitochondrial | ALDH5A1 |
| 25 | Polyunsaturated fatty acid lipoxygenase ALOX15 | ALOX15 |
| 26 | Polyunsaturated fatty acid lipoxygenase ALOX15B | Alox15b |
| 27 | Polyunsaturated fatty acid 5-lipoxygenase | ALOX5 |
| 28 | Polyunsaturated fatty acid 5-lipoxygenase | ALOX5 |
| 29 | Alkaline phosphatase, tissue-nonspecific isozyme | ALPL |
| 30 | Aminopeptidase N | ANPEP |
| 31 | Apoptotic protease-activating factor 1 | APAF1 |
| 32 | DNA dC->dU-editing enzyme APOBEC-3A | APOBEC3A |
| 33 | DNA dC->dU-editing enzyme APOBEC-3G | APOBEC3G |
| 34 | Amyloid-beta precursor protein | APP |
| 35 | Androgen receptor | AR |
| 36 | Potassium-transporting ATPase alpha chain 1 | ATP4A |
| 37 | Beta-secretase 1 | BACE1 |
| 38 | Bcl-2 homologous antagonist/killer | BAK1 |
| 39 | Apoptosis regulator BAX | BAX |
| 40 | Bcl-2-binding component 3, isoforms 3/4 | BBC3 |
| 41 | Basal cell adhesion molecule | BCAM |
| 42 | Cholinesterase | BCHE |
| 43 | Apoptosis regulator Bcl-2 | BCL2 |
| 44 | Bcl-2-related protein A1 | BCL2A1 |
| 45 | Bcl-2-like protein 1 | BCL2L1 |
| 46 | Bcl-2-like protein 11 | BCL2L11 |
| 47 | Baculoviral IAP repeat-containing protein 3 | BIRC3 |
| 48 | Baculoviral IAP repeat-containing protein 5 | BIRC5 |
| 49 | Breast cancer type 1 susceptibility protein | BRCA1 |
| 50 | Breast cancer type 2 susceptibility protein | BRCA2 |
| 51 | Basigin | BSG |
| 52 | C5a anaphylatoxin chemotactic receptor 1 | C5AR1 |
| 53 | Carbonic anhydrase 1 | CA1 |
| 54 | Carbonic anhydrase 12 | CA12 |
| 55 | Carbonic anhydrase 13 | CA13 |
| 56 | Carbonic anhydrase 14 | CA14 |
| 57 | Carbonic anhydrase 15 | Ca15 |
| 58 | Carbonic anhydrase 2 | CA2 |
| 59 | Carbonic anhydrase 3 | CA3 |
| 60 | Carbonic anhydrase 4 | CA4 |
| 61 | Carbonic anhydrase 5A, mitochondrial | CA5A |
| 62 | Carbonic anhydrase 5B, mitochondrial | CA5B |
| 63 | Carbonic anhydrase 6 | CA6 |
| 64 | Carbonic anhydrase 7 | CA7 |
| 65 | Carbonic anhydrase 9 | CA9 |
| 66 | Voltage-dependent N-type calcium channel subunit alpha-1B | CACNA1B |
| 67 | Voltage-dependent T-type calcium channel subunit alpha-1H | CACNA1H |
| 68 | Caspase-3 | CASP3 |
| 69 | Caspase-9 | CASP9 |
| 70 | Catalase | CAT |
| 71 | Carbonyl reductase [NADPH] 1 | CBR1 |
| 72 | C-C motif chemokine 2 | CCL2 |
| 73 | G2/mitotic-specific cyclin-B1 | CCNB1 |
| 74 | G1/S-specific cyclin-D1 | CCND1 |
| 75 | G1/S-specific cyclin-D2 | CCND2 |
| 76 | G1/S-specific cyclin-E1 | CCNE1 |
| 77 | G1/S-specific cyclin-E2 | CCNE2 |
| 78 | C-C chemokine receptor type 2 | CCR2 |
| 79 | T-cell-specific surface glycoprotein CD28 | CD28 |
| 80 | CD320 antigen | CD320 |
| 81 | T-lymphocyte activation antigen CD80 | CD80 |
| 82 | M-phase inducer phosphatase 2 | CDC25B |
| 83 | Cell division control protein 42 homolog | CDC42 |
| 84 | Cyclin-dependent kinase 1 | CDK1 |
| 85 | Cyclin-dependent kinase 4 | CDK4 |
| 86 | Cyclin-dependent-like kinase 5 | CDK5 |
| 87 | Cyclin-dependent kinase 6 | CDK6 |
| 88 | Cyclin-dependent kinase 7 | CDK7 |
| 89 | Cyclin-dependent kinase inhibitor 1 | CDKN1A |
| 90 | Liver carboxylesterase 1 | CES1 |
| 91 | Cocaine esterase | CES2 |
| 92 | CASP8 and FADD-like apoptosis regulator | CFLAR |
| 93 | CHRNA7-FAM7A fusion protein | CHRFAM7A |
| 94 | Muscarinic acetylcholine receptor M1 | CHRM1 |
| 95 | Muscarinic acetylcholine receptor M2 | CHRM2 |
| 96 | Muscarinic acetylcholine receptor M4 | CHRM4 |
| 97 | Muscarinic acetylcholine receptor M5 | CHRM5 |
| 98 | Neuronal acetylcholine receptor subunit alpha-4 | CHRNA4 |
| 99 | Neuronal acetylcholine receptor subunit alpha-7 | CHRNA7 |
| 100 | CDGSH iron-sulfur domain-containing protein 1 | CISD1 |
| 101 | Dual specificity protein kinase CLK1 | CLK1 |
| 102 | Cannabinoid receptor 2 | CNR2 |
| 103 | Collagen alpha-1 | COL2A1 |
| 104 | CREB/ATF bZIP transcription factor | CREBZF |
| 105 | C-reactive protein | CRP |
| 106 | Casein kinase II subunit alpha | CSNK2A1 |
| 107 | Carboxy-terminal domain RNA polymerase II polypeptide A small phosphatase 1 | CTDSP1 |
| 108 | Catenin beta-1 | CTNNB1 |
| 109 | Cullin-3 | CUL3 |
| 110 | Stromal cell-derived factor 1 | CXCL12 |
| 111 | Interleukin-8 | CXCL8 |
| 112 | Cytochrome P450 11B1, mitochondrial | CYP11B1 |
| 113 | Cytochrome P450 11B2, mitochondrial | CYP11B2 |
| 114 | Steroid 17-alpha-hydroxylase/17,20 lyase | CYP17A1 |
| 115 | Aromatase | CYP19A1 |
| 116 | Cytochrome P450 1A1 | CYP1A1 |
| 117 | Cytochrome P450 1A2 | CYP1A2 |
| 118 | Cytochrome P450 1B1 | CYP1B1 |
| 119 | 25-hydroxyvitamin D-1 alpha hydroxylase, mitochondrial | Cyp27b1 |
| 120 | Cytochrome P450 2C19 | CYP2C19 |
| 121 | Cytochrome P450 2C9 | CYP2C9 |
| 122 | Cytochrome P450 3A4 | CYP3A4 |
| 123 | Cysteinyl leukotriene receptor 1 | CYSLTR1 |
| 124 | DDIT3 upstream open reading frame protein | DDIT3 |
| 125 | Dihydroorotate dehydrogenase | DHODH |
| 126 | Dipeptidase 1 | DPEP1 |
| 127 | Dipeptidyl peptidase 4 | DPP4 |
| 128 | D(1A) dopamine receptor | DRD1 |
| 129 | D(2) dopamine receptor | DRD2 |
| 130 | D(4) dopamine receptor | DRD4 |
| 131 | D(1B) dopamine receptor | DRD5 |
| 132 | Dual oxidase 2 | DUOX2 |
| 133 | Dual specificity protein phosphatase 3 | DUSP3 |
| 134 | Dual specificity tyrosine-phosphorylation-regulated kinase 1A | DYRK1A |
| 135 | Dual specificity tyrosine-phosphorylation-regulated kinase 1B | DYRK1B |
| 136 | Endothelin-1 | EDN1 |
| 137 | Epidermal growth factor receptor | EGFR |
| 138 | Eukaryotic translation initiation factor 2 subunit 1 | EIF2S1 |
| 139 | Eukaryotic translation initiation factor 6 | EIF6 |
| 140 | Serine/threonine-protein kinase/endoribonuclease IRE1 | ERN1 |
| 141 | Estrogen receptor | ESR1 |
| 142 | Estrogen receptor beta | ESR2 |
| 143 | Steroid hormone receptor ERR1 | ESRRA |
| 144 | Steroid hormone receptor ERR2 | ESRRB |
| 145 | Tissue factor | F3 |
| 146 | Receptor-type tyrosine-protein kinase FLT3 | FLT3 |
| 147 | Proto-oncogene c-Fos | FOS |
| 148 | Forkhead box protein O1 | FOXO1 |
| 149 | Forkhead box protein O3 | FOXO3 |
| 150 | Tyrosine-protein kinase Fyn | FYN |
| 151 | Glucose-6-phosphatase | G6PC |
| 152 | Galanin receptor type 3 | GALR3 |
| 153 | GTP cyclohydrolase 1 | GCH1 |
| 154 | FAD-linked sulfhydryl oxidase ALR | GFER |
| 155 | Gap junction alpha-1 protein | GJA1 |
| 156 | Lactoylglutathione lyase | GLO1 |
| 157 | G-protein coupled receptor 35 | GPR35 |
| 158 | Glutamate receptor 2 | GRIA2 |
| 159 | Glutamate receptor ionotropic, NMDA 1 | GRIN1 |
| 160 | Glutamate receptor ionotropic, NMDA 2B | GRIN2B |
| 161 | Metabotropic glutamate receptor 4 | GRM4 |
| 162 | Glycogen synthase kinase-3 alpha | GSK3A |
| 163 | Glycogen synthase kinase-3 beta | GSK3B |
| 164 | Hydroxycarboxylic acid receptor 2 | HCAR2 |
| 165 | Histone deacetylase 1 | HDAC1 |
| 166 | Histone deacetylase 2 | HDAC2 |
| 167 | Histone deacetylase 3 | HDAC3 |
| 168 | Histone deacetylase 4 | HDAC4 |
| 169 | Histone deacetylase 6 | HDAC6 |
| 170 | Histone deacetylase 8 | HDAC8 |
| 171 | Hepatocyte growth factor | HGF |
| 172 | Hypoxia-inducible factor 1-alpha | HIF1A |
| 173 | Hepatocyte nuclear factor 4-alpha | HNF4A |
| 174 | Corticosteroid 11-beta-dehydrogenase isozyme 1 | Hsd11b1 |
| 175 | 17-beta-hydroxysteroid dehydrogenase type 1 | HSD17B1 |
| 176 | 17-beta-hydroxysteroid dehydrogenase 14 | HSD17B14 |
| 177 | 17-beta-hydroxysteroid dehydrogenase type 2 | HSD17B2 |
| 178 | Testosterone 17-beta-dehydrogenase 3 | Hsd17b3 |
| 179 | Heat shock protein HSP 90-alpha | HSP90AA1 |
| 180 | Heat shock protein HSP 90-beta | HSP90AB1 |
| 181 | Endoplasmic reticulum chaperone BiP | HSPA5 |
| 182 | 5-hydroxytryptamine receptor 1E | HTR1E |
| 183 | 5-hydroxytryptamine receptor 2C | HTR2C |
| 184 | 5-hydroxytryptamine receptor 5A | HTR5A |
| 185 | 5-hydroxytryptamine receptor 6 | HTR6 |
| 186 | Intercellular adhesion molecule 1 | ICAM1 |
| 187 | Insulin-like growth factor 1 receptor | IGF1R |
| 188 | Interleukin-10 | IL10 |
| 189 | Interleukin-17B | IL17B |
| 190 | Interleukin-1 alpha | IL1A |
| 191 | Interleukin-1 beta | IL1B |
| 192 | Interleukin-6 | IL6 |
| 193 | Oligo-1,6-glucosidase IMA1 | IMA1 |
| 194 | Insulin receptor | INSR |
| 195 | Insulin receptor substrate 1 | IRS1 |
| 196 | Integrin beta-1 | ITGB1 |
| 197 | Tyrosine-protein kinase JAK1 | JAK1 |
| 198 | Transcription factor AP-1 | JUN |
| 199 | Potassium channel subfamily K member 2 | KCNK2 |
| 200 | Lysine-specific histone demethylase 1A | KDM1A |
| 201 | Kelch-like ECH-associated protein 1 | KEAP1 |
| 202 | Mast/stem cell growth factor receptor Kit | KIT |
| 203 | Krueppel-like factor 10 | KLF10 |
| 204 | Tyrosine-protein kinase Lck | LCK |
| 205 | Lengsin | LGSN |
| 206 | Leukotriene B4 receptor 1 | LTB4R |
| 207 | Amine oxidase [flavin-containing] A | MAOA |
| 208 | Amine oxidase [flavin-containing] B | MAOB |
| 209 | Mitogen-activated protein kinase 1 | MAPK1 |
| 210 | Mitogen-activated protein kinase 3 | MAPK3 |
| 211 | Mitogen-activated protein kinase 8 | MAPK8 |
| 212 | MAP kinase-activated protein kinase 2 | MAPKAPK2 |
| 213 | Microtubule-associated protein tau | MAPT |
| 214 | Induced myeloid leukemia cell differentiation protein Mcl-1 | MCL1 |
| 215 | E3 ubiquitin-protein ligase Mdm2 | MDM2 |
| 216 | Methionine aminopeptidase 2 | METAP2 |
| 217 | Maltase-glucoamylase, intestinal [Includes: Maltase | MGAM |
| 218 | Monoglyceride lipase | MGLL |
| 219 | Macrophage migration inhibitory factor | MIF |
| 220 | Interstitial collagenase | MMP1 |
| 221 | Macrophage metalloelastase | MMP12 |
| 222 | 72 kDa type IV collagenase | MMP2 |
| 223 | Matrix metalloproteinase-9 | MMP9 |
| 224 | Myeloperoxidase | MPO |
| 225 | Serine/threonine-protein kinase mTOR | MTOR |
| 226 | Myc proto-oncogene protein | MYC |
| 227 | Nuclear receptor coactivator 2 | NCOA2 |
| 228 | Nuclear factor erythroid 2-related factor 2 | NFE2L2 |
| 229 | NF-kappa-B inhibitor alpha | NFKBIA |
| 230 | Nitric oxide synthase, inducible | NOS2 |
| 231 | Nitric oxide synthase, endothelial | NOS3 |
| 232 | NAD(P)H dehydrogenase [quinone] 1 | NQO1 |
| 233 | Quinone reductase 2 | NQO2 |
| 234 | Oxysterols receptor LXR-alpha | NR1H3 |
| 235 | Bile acid receptor | NR1H4 |
| 236 | COUP transcription factor 2 | NR2F2 |
| 237 | High affinity nerve growth factor receptor | NTRK1 |
| 238 | Ornithine decarboxylase | ODC1 |
| 239 | Delta-type opioid receptor | OPRD1 |
| 240 | Kappa-type opioid receptor | OPRK1 |
| 241 | Mu-type opioid receptor | OPRM1 |
| 242 | Pappalysin-1 | PAPPA |
| 243 | cAMP-specific 3',5'-cyclic phosphodiesterase 4D | PDE4D |
| 244 | Platelet-derived growth factor receptor alpha | PDGFRA |
| 245 | Platelet-derived growth factor receptor beta | PDGFRB |
| 246 | Platelet endothelial cell adhesion molecule | PECAM1 |
| 247 | Phosphatidylinositol N-acetylglucosaminyltransferase subunit A | pigA |
| 248 | Phosphatidylinositol 4,5-bisphosphate 3-kinase catalytic subunit alpha isoform | PIK3CA |
| 249 | Phosphatidylinositol 4,5-bisphosphate 3-kinase catalytic subunit beta isoform | PIK3CB |
| 250 | Serine/threonine-protein kinase pim-1 | PIM1 |
| 251 | Phospholipase A2 | PLA2G1B |
| 252 | Tissue-type plasminogen activator | PLAT |
| 253 | Urokinase-type plasminogen activator | PLAU |
| 254 | Perilipin-1 | PLIN1 |
| 255 | Perilipin-5 | PLIN5 |
| 256 | Phorbol-12-myristate-13-acetate-induced protein 1 | PMAIP1 |
| 257 | DNA polymerase beta | POLB |
| 258 | Serum paraoxonase/arylesterase 1 | PON1 |
| 259 | Peroxisome proliferator-activated receptor alpha | PPARA |
| 260 | Peroxisome proliferator-activated receptor gamma | PPARG |
| 261 | Polyphenol oxidase 2 | PPO2 |
| 262 | cAMP-dependent protein kinase catalytic subunit alpha | PRKACA |
| 263 | 5'-AMP-activated protein kinase subunit gamma-2 | PRKAG2 |
| 264 | Protein kinase C alpha type | PRKCA |
| 265 | Protein kinase C beta type | PRKCB |
| 266 | Protein kinase C delta type | PRKCD |
| 267 | Protein kinase C epsilon type | PRKCE |
| 268 | Protein kinase C gamma type | PRKCG |
| 269 | Serine/threonine-protein kinase D1 | PRKD1 |
| 270 | Trypsin-2 | PRSS2 |
| 271 | Phosphotyrosine protein phosphatase | ptbB |
| 272 | Phosphatidylinositol 3,4,5-trisphosphate 3-phosphatase and dual-specificity protein phosphatase PTEN | PTEN |
| 273 | Prostaglandin E2 receptor EP2 subtype | PTGER2 |
| 274 | Prostaglandin E synthase | PTGES |
| 275 | Prostaglandin G/H synthase 1 | PTGS1 |
| 276 | Prostaglandin G/H synthase 2 | PTGS2 |
| 277 | Focal adhesion kinase 1 | PTK2 |
| 278 | Protein-tyrosine kinase 2-beta | PTK2B |
| 279 | Heparin-binding growth factor 2 | PTN |
| 280 | Tyrosine-protein phosphatase non-receptor type 1 | PTPN1 |
| 281 | Tyrosine-protein phosphatase non-receptor type 22 | PTPN22 |
| 282 | Tyrosine-protein phosphatase non-receptor type 7 | PTPN7 |
| 283 | Receptor-type tyrosine-protein phosphatase C | PTPRC |
| 284 | Pygopus homolog 1 | PYGO1 |
| 285 | Ras-related C3 botulinum toxin substrate 1 | RAC1 |
| 286 | RAF proto-oncogene serine/threonine-protein kinase | RAF1 |
| 287 | Retinoic acid receptor alpha | RARA |
| 288 | Retinoic acid receptor beta | RARB |
| 289 | Retinoic acid receptor gamma | RARG |
| 290 | Transcription factor p65 | RELA |
| 291 | Proto-oncogene tyrosine-protein kinase receptor Ret | RET |
| 292 | Receptor-interacting serine/threonine-protein kinase 2 | RIPK2 |
| 293 | Nuclear receptor ROR-alpha | RORA |
| 294 | 40S ribosomal protein S6 | RPS6 |
| 295 | Ribosomal protein S6 kinase alpha-3 | RPS6KA3 |
| 296 | Protein CBFA2T1 | RUNX1T1 |
| 297 | Protein S100-A9 | S100A9 |
| 298 | Sphingosine 1-phosphate receptor 1 | S1PR1 |
| 299 | Sphingosine 1-phosphate receptor 2 | S1PR2 |
| 300 | Sphingosine 1-phosphate receptor 4 | S1PR4 |
| 301 | Sphingosine 1-phosphate receptor 5 | S1PR5 |
| 302 | Sterol carrier protein 2 | SCP-2 |
| 303 | E-selectin | SELE |
| 304 | Plasminogen activator inhibitor 1 | SERPINE1 |
| 305 | Sex hormone-binding globulin | SHBG |
| 306 | Sigma non-opioid intracellular receptor 1 | SIGMAR1 |
| 307 | NAD-dependent protein deacetylase sirtuin-1 | SIRT1 |
| 308 | NAD-dependent protein deacetylase sirtuin-2 | SIRT2 |
| 309 | Solute carrier family 2, facilitated glucose transporter member 4 | SLC2A4 |
| 310 | Sodium-dependent noradrenaline transporter | SLC6A2 |
| 311 | Sodium-dependent dopamine transporter | SLC6A3 |
| 312 | Superoxide dismutase [Cu-Zn] | SOD1 |
| 313 | Superoxide dismutase [Mn], mitochondrial | SOD2 |
| 314 | SPARC | SPARC |
| 315 | Proto-oncogene tyrosine-protein kinase Src | SRC |
| 316 | Sterol regulatory element-binding protein 1 | SREBF1 |
| 317 | Signal transducer and activator of transcription 1-alpha/beta | STAT1 |
| 318 | Signal transducer and activator of transcription 3 | STAT3 |
| 319 | Steryl-sulfatase | STS |
| 320 | Sulfotransferase 1E1 | SULT1E1 |
| 321 | Tyrosine-protein kinase SYK | SYK |
| 322 | Trace amine-associated receptor 1 | TAAR1 |
| 323 | Telomerase protein component 1 | TEP1 |
| 324 | Telomerase reverse transcriptase | TERT |
| 325 | Protransforming growth factor alpha [Cleaved into: Transforming growth factor alpha | TGFA |
| 326 | Transforming growth factor beta-1 proprotein [Cleaved into: Latency-associated peptide | TGFB1 |
| 327 | Transforming growth factor beta-2 proprotein | TGFB2 |
| 328 | Thyroid hormone receptor alpha | THRA |
| 329 | Toll-like receptor 9 | TLR9 |
| 330 | Tumor necrosis factor | TNF |
| 331 | Tumor necrosis factor receptor superfamily member 10A | TNFRSF10A |
| 332 | Tumor necrosis factor receptor superfamily member 10B | TNFRSF10B |
| 333 | Tumor necrosis factor ligand superfamily member 10 | TNFSF10 |
| 334 | Cellular tumor antigen p53 | TP53 |
| 335 | TNF receptor-associated factor 2 | TRAF2 |
| 336 | Transient receptor potential cation channel subfamily A member 1 | Trpa1 |
| 337 | Transthyretin | TTR |
| 338 | Tubulin alpha-1A chain | TUBA1A |
| 339 | Tubulin beta-1 chain | TUBB1 |
| 340 | Tubulin beta-2B chain | TUBB2B |
| 341 | Tubulin beta-3 chain | TUBB3 |
| 342 | Tyrosinase | TYR |
| 343 | Vascular cell adhesion protein 1 | VCAM1 |
| 344 | Vascular endothelial growth factor A | VEGFA |
| 345 | Xanthine dehydrogenase/oxidase [Includes: Xanthine dehydrogenase | XDH |
| 346 | E3 ubiquitin-protein ligase XIAP | XIAP |
| 347 | X-ray repair cross-complementing protein 6 | XRCC6 |
