# Supplementary material for: A network pharmacology study on mechanism of resveratrol in treating preeclampsia via regulation of AGE-RAGE and HIF-1 signalling pathways
Source: Front Endocrinol (Lausanne). 2023 Jan 5;13:1044775. doi: 10.3389/fendo.2022.1044775 (PMC9849370; doi:10.3389/fendo.2022.1044775)
Supplement: Supplementary file 2 [file Table_2.docx]

**Additional file 2. The PE-related genes**

| **No.** | **Target Name** | **Gene Symbol** |
| --- | --- | --- |
| 1 | Preeclampsia/Eclampsia 1 | PEE1 |
| 2 | Preeclampsia/Eclampsia 2 | PEE2 |
| 3 | Preeclampsia/Eclampsia 3 | PEE3 |
| 4 | Fms Related Receptor Tyrosine Kinase 1 | FLT1 |
| 5 | Storkhead Box 1 | STOX1 |
| 6 | Corin, Serine Peptidase | CORIN |
| 7 | Nitric Oxide Synthase 3 | NOS3 |
| 8 | Coagulation Factor V | F5 |
| 9 | Placental Growth Factor | PGF |
| 10 | Angiotensinogen | AGT |
| 11 | Endoglin | ENG |
| 12 | Methylenetetrahydrofolate Reductase | MTHFR |
| 13 | Nuclear Receptor Binding SET Domain Protein 1 | NSD1 |
| 14 | Vascular Endothelial Growth Factor A | VEGFA |
| 15 | Coagulation Factor II, Thrombin | F2 |
| 16 | Angiotensin I Converting Enzyme | ACE |
| 17 | Serpin Family E Member 1 | SERPINE1 |
| 18 | Complement Factor H | CFH |
| 19 | Tumor Necrosis Factor | TNF |
| 20 | Endothelin 1 | EDN1 |
| 21 | Interleukin 6 | IL6 |
| 22 | Leptin | LEP |
| 23 | Interleukin 10 | IL10 |
| 24 | C-Reactive Protein | CRP |
| 25 | Angiotensin II Receptor Type 1 | AGTR1 |
| 26 | Apolipoprotein H | APOH |
| 27 | Matrix Metallopeptidase 9 | MMP9 |
| 28 | Major Histocompatibility Complex, Class I, G | HLA-G |
| 29 | Pappalysin 1 | PAPPA |
| 30 | Hypoxia Inducible Factor 1 Subunit Alpha | HIF1A |
| 31 | Von Willebrand Factor | VWF |
| 32 | Galectin 13 | LGALS13 |
| 33 | Coagulation Factor III, Tissue Factor | F3 |
| 34 | Adiponectin, C1Q And Collagen Domain Containing | ADIPOQ |
| 35 | Serpin Family C Member 1 | SERPINC1 |
| 36 | Toll Like Receptor 4 | TLR4 |
| 37 | Thrombomodulin | THBD |
| 38 | Activin A Receptor Type 2A | ACVR2A |
| 39 | Selectin P | SELP |
| 40 | Hemoglobin Subunit Alpha 1 | HBA1 |
| 41 | Selectin E | SELE |
| 42 | Complement Factor I | CFI |
| 43 | Vascular Cell Adhesion Molecule 1 | VCAM1 |
| 44 | MicroRNA 210 | MIR210 |
| 45 | Renin | REN |
| 46 | C-X-C Motif Chemokine Ligand 8 | CXCL8 |
| 47 | Kinase Insert Domain Receptor | KDR |
| 48 | Epoxide Hydrolase 1 | EPHX1 |
| 49 | Plasminogen Activator, Tissue Type | PLAT |
| 50 | Peroxisome Proliferator Activated Receptor Gamma | PPARG |
| 51 | CD46 Molecule | CD46 |
| 52 | Insulin Like Growth Factor 1 | IGF1 |
| 53 | Heme Oxygenase 1 | HMOX1 |
| 54 | Interleukin 1 Beta | IL1B |
| 55 | Adrenomedullin | ADM |
| 56 | Inhibin Subunit Alpha | INHA |
| 57 | Hemoglobin Subunit Alpha 2 | HBA2 |
| 58 | Superoxide Dismutase 1 | SOD1 |
| 59 | Paraoxonase 1 | PON1 |
| 60 | Intercellular Adhesion Molecule 1 | ICAM1 |
| 61 | HELLP Associated Long Non-Coding RNA | HELLPAR |
| 62 | Oxidized Low Density Lipoprotein Receptor 1 | OLR1 |
| 63 | Uncharacterized LOC101927179 | LOC101927179 |
| 64 | Endogenous Retrovirus Group W Member 1, Envelope | ERVW-1 |
| 65 | Leptin Receptor | LEPR |
| 66 | Natriuretic Peptide A | NPPA |
| 67 | Pappalysin 2 | PAPPA2 |
| 68 | Interleukin 4 | IL4 |
| 69 | Transforming Growth Factor Beta 3 | TGFB3 |
| 70 | Interleukin 18 | IL18 |
| 71 | Hydroxysteroid 11-Beta Dehydrogenase 2 | HSD11B2 |
| 72 | Annexin A5 | ANXA5 |
| 73 | Selectin L | SELL |
| 74 | Cytochrome P450 Family 11 Subfamily B Member 2 | CYP11B2 |
| 75 | Cystatin C | CST3 |
| 76 | Insulin Like Growth Factor Binding Protein 1 | IGFBP1 |
| 77 | Insulin | INS |
| 78 | Serpin Family B Member 2 | SERPINB2 |
| 79 | Advanced Glycosylation End-Product Specific Receptor | AGER |
| 80 | Alpha Fetoprotein | AFP |
| 81 | Corticotropin Releasing Hormone | CRH |
| 82 | Nitric Oxide Synthase 2 | NOS2 |
| 83 | Tyrosine 3-Monooxygenase/Tryptophan 5-Monooxygenase Activation Protein Epsilon Pseudogene 7 | YWHAEP7 |
| 84 | CEA Cell Adhesion Molecule Pseudogene 8 | CEACAMP8 |
| 85 | FLT1 Pseudogene 1 | FLT1P1 |
| 86 | Hemoglobin Subunit Alpha 2 Recombination Region | LOC106804612 |
| 87 | ADAM Metallopeptidase Domain 12 | ADAM12 |
| 88 | Albumin | ALB |
| 89 | Angiotensin II Receptor Type 2 | AGTR2 |
| 90 | Urotensin 2 | UTS2 |
| 91 | Interleukin 15 | IL15 |
| 92 | KiSS-1 Metastasis Suppressor | KISS1 |
| 93 | Catechol-O-Methyltransferase | COMT |
| 94 | Phosphatidylinositol Glycan Anchor Biosynthesis Class F | PIGF |
| 95 | Integrin Subunit Alpha M | ITGAM |
| 96 | Interleukin 16 | IL16 |
| 97 | Platelet And Endothelial Cell Adhesion Molecule 1 | PECAM1 |
| 98 | E1A Binding Protein P300 | EP300 |
| 99 | Dimethylarginine Dimethylaminohydrolase 2 | DDAH2 |
| 100 | H19 Imprinted Maternally Expressed Transcript | H19 |
| 101 | Endoplasmic Reticulum Aminopeptidase 2 | ERAP2 |
| 102 | Chorionic Gonadotropin Subunit Beta 3 | CGB3 |
| 103 | Leucyl And Cystinyl Aminopeptidase | LNPEP |
| 104 | Fibronectin 1 | FN1 |
| 105 | Oxytocin/Neurophysin I Prepropeptide | OXT |
| 106 | Insulin Like Growth Factor Binding Protein 3 | IGFBP3 |
| 107 | Kininogen 1 | KNG1 |
| 108 | Major Histocompatibility Complex, Class I, C | HLA-C |
| 109 | Bradykinin Receptor B2 | BDKRB2 |
| 110 | MicroRNA 126 | MIR126 |
| 111 | Pro-Platelet Basic Protein | PPBP |
| 112 | Plasminogen Activator, Urokinase | PLAU |
| 113 | Pregnancy Specific Beta-1-Glycoprotein 1 | PSG1 |
| 114 | Tachykinin Precursor 3 | TAC3 |
| 115 | Insulin Like Growth Factor 2 | IGF2 |
| 116 | Metastasis Associated Lung Adenocarcinoma Transcript 1 | MALAT1 |
| 117 | Cellular Communication Network Factor 1 | CCN1 |
| 118 | Placenta Enriched 1 | PLAC1 |
| 119 | WAP Four-Disulfide Core Domain 21, Pseudogene | WFDC21P |
| 120 | Indoleamine 2,3-Dioxygenase 1 | IDO1 |
| 121 | MicroRNA 125b-1 | MIR125B1 |
| 122 | Heparin Binding EGF Like Growth Factor | HBEGF |
| 123 | Glial Cells Missing Transcription Factor 1 | GCM1 |
| 124 | Adhesion G Protein-Coupled Receptor G6 | ADGRG6 |
| 125 | Forkhead Box P3 | FOXP3 |
| 126 | Glutamyl Aminopeptidase | ENPEP |
| 127 | HOX Transcript Antisense RNA | HOTAIR |
| 128 | Killer Cell Immunoglobulin Like Receptor, Two Ig Domains And Long Cytoplasmic Tail 4 | KIR2DL4 |
| 129 | CD63 Molecule | CD63 |
| 130 | Tachykinin Receptor 3 | TACR3 |
| 131 | Serpin Family F Member 2 | SERPINF2 |
| 132 | Cytochrome P450 Family 11 Subfamily B Member 1 | CYP11B1 |
| 133 | Glutamic--Pyruvic Transaminase | GPT |
| 134 | Inhibin Subunit Beta B | INHBB |
| 135 | Decorin | DCN |
| 136 | Nitric Oxide Synthase Trafficking | NOSTRIN |
| 137 | Haptoglobin | HP |
| 138 | MicroRNA 214 | MIR214 |
| 139 | ADAM Metallopeptidase With Thrombospondin Type 1 Motif 13 | ADAMTS13 |
| 140 | Follistatin | FST |
| 141 | Mitogen-Activated Protein Kinase 1 | MAPK1 |
| 142 | ERCC Excision Repair 2, TFIIH Core Complex Helicase Subunit | ERCC2 |
| 143 | Glutathione Peroxidase 3 | GPX3 |
| 144 | Retinol Binding Protein 4 | RBP4 |
| 145 | Angiopoietin 2 | ANGPT2 |
| 146 | Matrix Metallopeptidase 2 | MMP2 |
| 147 | Phosphodiesterase 5A | PDE5A |
| 148 | SPRY4 Intronic Transcript 1 | SPRY4-IT1 |
| 149 | G Protein Subunit Beta 3 | GNB3 |
| 150 | Neuropilin 1 | NRP1 |
| 151 | Chorionic Gonadotropin Subunit Beta 7 | CGB7 |
| 152 | Aminolevulinate Dehydratase | ALAD |
| 153 | Estrogen Receptor 1 | ESR1 |
| 154 | Integrin Subunit Alpha 1 | ITGA1 |
| 155 | Chorionic Somatomammotropin Hormone 1 | CSH1 |
| 156 | Chorionic Somatomammotropin Hormone 2 | CSH2 |
| 157 | Ferredoxin Reductase | FDXR |
| 158 | Apolipoprotein E | APOE |
| 159 | Chromosome 19 Open Reading Frame 33 | C19orf33 |
| 160 | Taurine Up-Regulated 1 | TUG1 |
| 161 | MicroRNA 574 | MIR574 |
| 162 | NLR Family Pyrin Domain Containing 7 | NLRP7 |
| 163 | CD40 Ligand | CD40LG |
| 164 | Lamin B Receptor | LBR |
| 165 | Nuclear Receptor Subfamily 1 Group H Member 4 | NR1H4 |
| 166 | Solute Carrier Family 25 Member 20 | SLC25A20 |
| 167 | ATP Binding Cassette Subfamily B Member 11 | ABCB11 |
| 168 | ATP Binding Cassette Subfamily B Member 4 | ABCB4 |
| 169 | Deoxyhypusine Synthase | DHPS |
| 170 | ATPase Phospholipid Transporting 8B1 | ATP8B1 |
| 171 | Apelin | APLN |
| 172 | AT-Rich Interaction Domain 1A | ARID1A |
| 173 | Angiopoietin 1 | ANGPT1 |
| 174 | Growth Arrest Specific 6 | GAS6 |
| 175 | Transforming Growth Factor Beta 1 | TGFB1 |
| 176 | ERCC Excision Repair 3, TFIIH Core Complex Helicase Subunit | ERCC3 |
| 177 | Tissue Factor Pathway Inhibitor 2 | TFPI2 |
| 178 | TEK Receptor Tyrosine Kinase | TEK |
| 179 | Hemoglobin Subunit Beta | HBB |
| 180 | Hemoglobin Subunit Zeta | HBZ |
| 181 | Glutathione S-Transferase Pi 1 | GSTP1 |
| 182 | Sialic Acid Binding Ig Like Lectin 6 | SIGLEC6 |
| 183 | Sex Hormone Binding Globulin | SHBG |
| 184 | MicroRNA 146a | MIR146A |
| 185 | Hydroxyacyl-CoA Dehydrogenase Trifunctional Multienzyme Complex Subunit Alpha | HADHA |
| 186 | Glucokinase | GCK |
| 187 | General Transcription Factor IIE Subunit 2 | GTF2E2 |
| 188 | General Transcription Factor IIH Subunit 5 | GTF2H5 |
| 189 | Ring Finger Protein 113A | RNF113A |
| 190 | M-Phase Specific PLK1 Interacting Protein | MPLKIP |
| 191 | Threonyl-TRNA Synthetase 1 | TARS1 |
| 192 | Hemoglobin Subunit Alpha 1 Recombination Region | LOC106804613 |
| 193 | HBB Recombination Region | LOC106099062 |
| 194 | Origin Of Replication At HBB | LOC107133510 |
| 195 | Beta-Globin Gene 3' Regulatory Region | LOC110006319 |
| 196 | Solute Carrier Family 17 Member 5 | SLC17A5 |
| 197 | TIMP Metallopeptidase Inhibitor 1 | TIMP1 |
| 198 | Interferon Gamma | IFNG |
| 199 | FGR Proto-Oncogene, Src Family Tyrosine Kinase | FGR |
| 200 | Interferon Alpha 1 | IFNA1 |
| 201 | Pentraxin 3 | PTX3 |
| 202 | Leptin, Serum Levels Of | LEPQTL1 |
| 203 | Glutathione S-Transferase Mu 1 | GSTM1 |
| 204 | Matrix Metallopeptidase 8 | MMP8 |
| 205 | Tumor Protein P53 | TP53 |
| 206 | Complement C3 | C3 |
| 207 | Protein Tyrosine Phosphatase Non-Receptor Type 11 | PTPN11 |
| 208 | BCR Activator Of RhoGEF And GTPase | BCR |
| 209 | HRas Proto-Oncogene, GTPase | HRAS |
| 210 | Actin Alpha 1, Skeletal Muscle | ACTA1 |
| 211 | L1 Cell Adhesion Molecule | L1CAM |
| 212 | Ryanodine Receptor 1 | RYR1 |
| 213 | CRK Like Proto-Oncogene, Adaptor Protein | CRKL |
| 214 | Solute Carrier Family 26 Member 3 | SLC26A3 |
| 215 | Ras Like Without CAAX 1 | RIT1 |
| 216 | 24-Dehydrocholesterol Reductase | DHCR24 |
| 217 | SEC24 Homolog C, COPII Coat Complex Component | SEC24C |
| 218 | Neuraminidase 1 | NEU1 |
| 219 | Histone Cell Cycle Regulator | HIRA |
| 220 | T-Box Transcription Factor 1 | TBX1 |
| 221 | Receptor Associated Protein Of The Synapse | RAPSN |
| 222 | Ras Responsive Element Binding Protein 1 | RREB1 |
| 223 | Glycoprotein Ib Platelet Subunit Beta | GP1BB |
| 224 | Jumonji Domain Containing 1C | JMJD1C |
| 225 | Thrombospondin Type 1 Domain Containing 1 | THSD1 |
| 226 | ARVCF Delta Catenin Family Member | ARVCF |
| 227 | Dynein Axonemal Heavy Chain 9 | DNAH9 |
| 228 | Natural Cytotoxicity Triggering Receptor 1 | NCR1 |
| 229 | Meiotic Double-Stranded Break Formation Protein 1 | MEI1 |
| 230 | Leucine Rich Repeat Containing 56 | LRRC56 |
| 231 | Ubiquitin Recognition Factor In ER Associated Degradation 1 | UFD1 |
| 232 | Chromosome 11 Open Reading Frame 80 | C11orf80 |
| 233 | KH Domain Containing 3 Like, Subcortical Maternal Complex Member | KHDC3L |
| 234 | Uncharacterized LOC100505549 | LOC100505549 |
| 235 | CYP11B1 Recombination Region | LOC106799833 |
| 236 | Sharpr-MPRA Regulatory Region 9902 | LOC112543448 |
| 237 | Chromosome 22q11.2 Deletion Syndrome, Distal | DEL22Q11.2 |
| 238 | Natriuretic Peptide B | NPPB |
| 239 | Glutathione S-Transferase Theta 1 | GSTT1 |
| 240 | Coagulation Factor X | F10 |
| 241 | Superoxide Dismutase 2 | SOD2 |
| 242 | Apelin Receptor Early Endogenous Ligand | APELA |
| 243 | Interleukin 17A | IL17A |
| 244 | Lipoprotein Lipase | LPL |
| 245 | Cadherin 5 | CDH5 |
| 246 | Nuclear Receptor Subfamily 3 Group C Member 2 | NR3C2 |
| 247 | HtrA Serine Peptidase 4 | HTRA4 |
| 248 | Transforming Growth Factor Beta 2 | TGFB2 |
| 249 | Transferrin | TF |
| 250 | Programmed Cell Death 1 | PDCD1 |
| 251 | Nicotinamide Phosphoribosyltransferase | NAMPT |
| 252 | Cytochrome P450 Family 17 Subfamily A Member 1 | CYP17A1 |
| 253 | Mannose Binding Lectin 2 | MBL2 |
| 254 | Carboxypeptidase B2 | CPB2 |
| 255 | High Mobility Group Box 1 | HMGB1 |
| 256 | Platelet Factor 4 | PF4 |
| 257 | Nuclear Factor, Erythroid 2 Like 2 | NFE2L2 |
| 258 | Insulin Receptor Substrate 1 | IRS1 |
| 259 | Lipocalin 2 | LCN2 |
| 260 | FTO Alpha-Ketoglutarate Dependent Dioxygenase | FTO |
| 261 | MicroRNA 21 | MIR21 |
| 262 | Myeloperoxidase | MPO |
| 263 | Interleukin 1 Receptor Antagonist | IL1RN |
| 264 | Growth Arrest And DNA Damage Inducible Alpha | GADD45A |
| 265 | Insulin Receptor | INSR |
| 266 | Interleukin 1 Alpha | IL1A |
| 267 | Growth Differentiation Factor 15 | GDF15 |
| 268 | Fas Cell Surface Death Receptor | FAS |
| 269 | Heat Shock Protein Family A (Hsp70) Member 4 | HSPA4 |
| 270 | Fatty Acid Binding Protein 4 | FABP4 |
| 271 | Complement Factor B | CFB |
| 272 | MicroRNA 155 | MIR155 |
| 273 | Matrix Metallopeptidase 3 | MMP3 |
| 274 | Histidine Rich Glycoprotein | HRG |
| 275 | Cytochrome P450 Family 1 Subfamily A Member 1 | CYP1A1 |
| 276 | Ectonucleotide Pyrophosphatase/Phosphodiesterase 1 | ENPP1 |
| 277 | Ceruloplasmin | CP |
| 278 | Adrenoceptor Beta 3 | ADRB3 |
| 279 | NPHS1 Adhesion Molecule, Nephrin | NPHS1 |
| 280 | Vitamin D Receptor | VDR |
| 281 | Annexin A2 | ANXA2 |
| 282 | Adrenoceptor Beta 1 | ADRB1 |
| 283 | Calpain 10 | CAPN10 |
| 284 | C-C Motif Chemokine Ligand 2 | CCL2 |
| 285 | Resistin | RETN |
| 286 | Arginine Vasopressin | AVP |
| 287 | Cytotoxic T-Lymphocyte Associated Protein 4 | CTLA4 |
| 288 | Interleukin 2 | IL2 |
| 289 | Epidermal Growth Factor Receptor | EGFR |
| 290 | Endoplasmic Reticulum Protein 44 | ERP44 |
| 291 | Lymphotoxin Alpha | LTA |
| 292 | Fibroblast Growth Factor 2 | FGF2 |
| 293 | Suppressor Of Cytokine Signaling 3 | SOCS3 |
| 294 | MicroRNA 517a | MIR517A |
| 295 | Interleukin 2 Receptor Subunit Alpha | IL2RA |
| 296 | Transthyretin | TTR |
| 297 | Integrin Subunit Alpha 2b | ITGA2B |
| 298 | Toll Like Receptor 3 | TLR3 |
| 299 | Coagulation Factor XIII A Chain | F13A1 |
| 300 | Solute Carrier Family 22 Member 5 | SLC22A5 |
| 301 | Prostaglandin-Endoperoxide Synthase 2 | PTGS2 |
| 302 | Interleukin 5 | IL5 |
| 303 | HtrA Serine Peptidase 1 | HTRA1 |
| 304 | Calcitonin Related Polypeptide Alpha | CALCA |
| 305 | Urocortin | UCN |
| 306 | Adducin 1 | ADD1 |
| 307 | Colony Stimulating Factor 2 | CSF2 |
| 308 | MicroRNA 149 | MIR149 |
| 309 | ADAM Metallopeptidase With Thrombospondin Type 1 Motif 12 | ADAMTS12 |
| 310 | Regulator Of G Protein Signaling 2 | RGS2 |
| 311 | Major Histocompatibility Complex, Class II, DR Beta 1 | HLA-DRB1 |
| 312 | Transforming Growth Factor Beta Receptor 1 | TGFBR1 |
| 313 | Caspase 3 | CASP3 |
| 314 | CD14 Molecule | CD14 |
| 315 | Alpha-1-Microglobulin/Bikunin Precursor | AMBP |
| 316 | Plasminogen | PLG |
| 317 | Calcium Voltage-Gated Channel Subunit Alpha1 C | CACNA1C |
| 318 | A-Kinase Anchoring Protein 12 | AKAP12 |
| 319 | REV1 DNA Directed Polymerase | REV1 |
| 320 | Myosin ID | MYO1D |
| 321 | CTD Small Phosphatase Like | CTDSPL |
| 322 | LDL Receptor Related Protein 1B | LRP1B |
| 323 | Docking Protein 6 | DOK6 |
| 324 | Microtubule Associated Scaffold Protein 1 | MTUS1 |
| 325 | Scm Like With Four Mbt Domains 1 | SFMBT1 |
| 326 | Kinesin Family Member 26B | KIF26B |
| 327 | Coiled-Coil Domain Containing 102B | CCDC102B |
| 328 | Achaete-Scute Family BHLH Transcription Factor 3 | ASCL3 |
| 329 | BPI Fold Containing Family A Member 3 | BPIFA3 |
| 330 | Lamin Tail Domain Containing 1 | LMNTD1 |
| 331 | Von Willebrand Factor D And EGF Domains | VWDE |
| 332 | Uncharacterized LOC730100 | LOC730100 |
| 333 | Potassium Voltage-Gated Channel Subfamily E Regulatory Subunit 1 | KCNE1 |
| 334 | Major Histocompatibility Complex, Class II, DQ Beta 1 | HLA-DQB1 |
| 335 | Vascular Endothelial Growth Factor C | VEGFC |
| 336 | 5-Methyltetrahydrofolate-Homocysteine Methyltransferase Reductase | MTRR |
| 337 | Lipase C, Hepatic Type | LIPC |
| 338 | Adiponectin Receptor 1 | ADIPOR1 |
| 339 | S100 Calcium Binding Protein B | S100B |
| 340 | Nitric Oxide Synthase 1 | NOS1 |
| 341 | Toll Like Receptor 2 | TLR2 |
| 342 | Chymase 1 | CMA1 |
| 343 | Major Histocompatibility Complex, Class I, A | HLA-A |
| 344 | TIMP Metallopeptidase Inhibitor 2 | TIMP2 |
| 345 | Nuclear Receptor Subfamily 3 Group C Member 1 | NR3C1 |
| 346 | Alpha Hemoglobin Stabilizing Protein | AHSP |
| 347 | C-X-C Motif Chemokine Ligand 10 | CXCL10 |
| 348 | NLR Family Pyrin Domain Containing 3 | NLRP3 |
| 349 | Tissue Factor Pathway Inhibitor | TFPI |
| 350 | Endoplasmic Reticulum Aminopeptidase 1 | ERAP1 |
| 351 | Hemopexin | HPX |
| 352 | C-X3-C Motif Chemokine Receptor 1 | CX3CR1 |
| 353 | C-X-C Motif Chemokine Ligand 12 | CXCL12 |
| 354 | Syndecan 1 | SDC1 |
| 355 | Nucleotide Binding Oligomerization Domain Containing 2 | NOD2 |
| 356 | Prolylcarboxypeptidase | PRCP |
| 357 | Interleukin 1 Receptor Like 1 | IL1RL1 |
| 358 | Major Histocompatibility Complex, Class I, E | HLA-E |
| 359 | Prokineticin 1 | PROK1 |
| 360 | Interleukin 13 | IL13 |
| 361 | RUNX Family Transcription Factor 1 | RUNX1 |
| 362 | C-Type Lectin Domain Family 4 Member A | CLEC4A |
| 363 | Maternally Expressed 3 | MEG3 |
| 364 | Estrogen Receptor 2 | ESR2 |
| 365 | Laeverin | LVRN |
| 366 | Solute Carrier Family 2 Member 1 | SLC2A1 |
| 367 | Apolipoprotein L1 | APOL1 |
| 368 | Ephrin B2 | EFNB2 |
| 369 | WNK Lysine Deficient Protein Kinase 1 | WNK1 |
| 370 | Inhibin Subunit Beta A | INHBA |
| 371 | Macrophage Migration Inhibitory Factor | MIF |
| 372 | Keratin 18 | KRT18 |
| 373 | MicroRNA 195 | MIR195 |
| 374 | EPH Receptor B4 | EPHB4 |
| 375 | Glutathione Peroxidase 4 | GPX4 |
| 376 | Clusterin | CLU |
| 377 | Protein C Receptor | PROCR |
| 378 | Sulfotransferase Family 1A Member 3 | SULT1A3 |
| 379 | Corticotropin Releasing Hormone Receptor 2 | CRHR2 |
| 380 | Mucin 16, Cell Surface Associated | MUC16 |
| 381 | Coagulation Factor VII | F7 |
| 382 | 5-Methyltetrahydrofolate-Homocysteine Methyltransferase | MTR |
| 383 | Acyl-CoA Dehydrogenase Very Long Chain | ACADVL |
| 384 | Ectonucleotide Pyrophosphatase/Phosphodiesterase 2 | ENPP2 |
| 385 | Superoxide Dismutase 3 | SOD3 |
| 386 | HNF1 Homeobox A | HNF1A |
| 387 | Endothelin Receptor Type B | EDNRB |
| 388 | Arginase 1 | ARG1 |
| 389 | Glutathione Peroxidase 1 | GPX1 |
| 390 | Aldo-Keto Reductase Family 1 Member C3 | AKR1C3 |
| 391 | Glycoprotein Hormones, Alpha Polypeptide | CGA |
| 392 | Follistatin Like 3 | FSTL3 |
| 393 | Androgen Receptor | AR |
| 394 | Fms Related Receptor Tyrosine Kinase 4 | FLT4 |
| 395 | Nuclear Receptor Subfamily 1 Group H Member 2 | NR1H2 |
| 396 | Integrin Subunit Alpha X | ITGAX |
| 397 | Hepatitis A Virus Cellular Receptor 2 | HAVCR2 |
| 398 | Chitinase 3 Like 1 | CHI3L1 |
| 399 | Vitronectin | VTN |
| 400 | Retinoic Acid Receptor Responder 2 | RARRES2 |
| 401 | Galectin 1 | LGALS1 |
| 402 | Glucose-6-Phosphate Dehydrogenase | G6PD |
| 403 | ERCC Excision Repair 1, Endonuclease Non-Catalytic Subunit | ERCC1 |
| 404 | Endothelin Receptor Type A | EDNRA |
| 405 | Epidermal Growth Factor | EGF |
| 406 | Cystathionine Beta-Synthase | CBS |
| 407 | Apolipoprotein A1 | APOA1 |
| 408 | Arginase 2 | ARG2 |
| 409 | Wnt Family Member 2 | WNT2 |
| 410 | Dimethylarginine Dimethylaminohydrolase 1 | DDAH1 |
| 411 | Elastase, Neutrophil Expressed | ELANE |
| 412 | C-C Motif Chemokine Ligand 5 | CCL5 |
| 413 | Major Histocompatibility Complex, Class II, DQ Alpha 1 | HLA-DQA1 |
| 414 | BCL2 Interacting Protein 3 | BNIP3 |
| 415 | C-X-C Motif Chemokine Receptor 4 | CXCR4 |
| 416 | Glucuronidase Beta | GUSB |
| 417 | Transferrin Receptor | TFRC |
| 418 | RELA Proto-Oncogene, NF-KB Subunit | RELA |
| 419 | Mitofusin 2 | MFN2 |
| 420 | Von Hippel-Lindau Tumor Suppressor | VHL |
| 421 | Toll Like Receptor 9 | TLR9 |
| 422 | Cystathionine Gamma-Lyase | CTH |
| 423 | Lipoprotein(A) | LPA |
| 424 | BCL2 Family Apoptosis Regulator BOK | BOK |
| 425 | Corticotropin Releasing Hormone Binding Protein | CRHBP |
| 426 | Fibronectin Type III Domain Containing 5 | FNDC5 |
| 427 | Matrix Metallopeptidase 1 | MMP1 |
| 428 | Thioredoxin | TXN |
| 429 | Coagulation Factor II Thrombin Receptor | F2R |
| 430 | Interleukin 27 | IL27 |
| 431 | Peroxisome Proliferator Activated Receptor Delta | PPARD |
| 432 | Nuclear Receptor Subfamily 1 Group H Member 3 | NR1H3 |
| 433 | V-Set And Immunoglobulin Domain Containing 4 | VSIG4 |
| 434 | CD274 Molecule | CD274 |
| 435 | TNF Receptor Superfamily Member 1A | TNFRSF1A |
| 436 | Brain Derived Neurotrophic Factor | BDNF |
| 437 | NADPH Oxidase 1 | NOX1 |
| 438 | HtrA Serine Peptidase 3 | HTRA3 |
| 439 | MicroRNA 152 | MIR152 |
| 440 | Melatonin Receptor 1B | MTNR1B |
| 441 | Death Domain Associated Protein | DAXX |
| 442 | BCL2 Apoptosis Regulator | BCL2 |
| 443 | Keratin 19 | KRT19 |
| 444 | Serine Protease 8 | PRSS8 |
| 445 | Interleukin 33 | IL33 |
| 446 | MET Proto-Oncogene, Receptor Tyrosine Kinase | MET |
| 447 | Hexokinase 2 Pseudogene 1 | HK2P1 |
| 448 | Peptidylprolyl Isomerase A | PPIA |
| 449 | MicroRNA 148a | MIR148A |
| 450 | Solute Carrier Family 9 Member A3 | SLC9A3 |
| 451 | Heat Shock Protein Family A (Hsp70) Member 1 Like | HSPA1L |
| 452 | CD74 Molecule | CD74 |
| 453 | Notch Receptor 1 | NOTCH1 |
| 454 | Matrix Metallopeptidase 14 | MMP14 |
| 455 | Protein Tyrosine Phosphatase Receptor Type O | PTPRO |
| 456 | Colony Stimulating Factor 1 | CSF1 |
| 457 | MicroRNA 141 | MIR141 |
| 458 | Chorionic Gonadotropin Subunit Beta 5 | CGB5 |
| 459 | Synaptopodin | SYNPO |
| 460 | MicroRNA 34a | MIR34A |
| 461 | Protein Kinase, DNA-Activated, Catalytic Subunit | PRKDC |
| 462 | ERCC Excision Repair 4, Endonuclease Catalytic Subunit | ERCC4 |
| 463 | ERCC Excision Repair 5, Endonuclease | ERCC5 |
| 464 | XPA, DNA Damage Recognition And Repair Factor | XPA |
| 465 | Hepcidin Antimicrobial Peptide | HAMP |
| 466 | Klotho | KL |
| 467 | Transforming Growth Factor Beta Receptor 2 | TGFBR2 |
| 468 | Enolase 2 | ENO2 |
| 469 | Delta Like Non-Canonical Notch Ligand 1 | DLK1 |
| 470 | Bone Morphogenetic Protein 6 | BMP6 |
| 471 | G Protein Subunit Alpha 12 | GNA12 |
| 472 | DNA Methyltransferase 3 Alpha | DNMT3A |
| 473 | Integrin Subunit Beta 2 | ITGB2 |
| 474 | Serpin Family A Member 1 | SERPINA1 |
| 475 | Heme Oxygenase 2 | HMOX2 |
| 476 | N-Acetyl-Alpha-Glucosaminidase | NAGLU |
| 477 | Semaphorin 3B | SEMA3B |
| 478 | MicroRNA 517c | MIR517C |
| 479 | MicroRNA 517b | MIR517B |
| 480 | Sirtuin 1 | SIRT1 |
| 481 | N-Myc Downstream Regulated 1 | NDRG1 |
| 482 | Fas Ligand | FASLG |
| 483 | Phosphatase And Tensin Homolog | PTEN |
| 484 | Integrin Subunit Beta 1 | ITGB1 |
| 485 | Catalase | CAT |
| 486 | Calcitonin Receptor Like Receptor | CALCRL |
| 487 | Natriuretic Peptide Receptor 3 | NPR3 |
| 488 | TNF Superfamily Member 13b | TNFSF13B |
| 489 | Vascular Endothelial Growth Factor B | VEGFB |
| 490 | C-X3-C Motif Chemokine Ligand 1 | CX3CL1 |
| 491 | Oncostatin M | OSM |
| 492 | Galectin 9 | LGALS9 |
| 493 | Dopamine Receptor D2 | DRD2 |
| 494 | Sodium Voltage-Gated Channel Alpha Subunit 5 | SCN5A |
| 495 | NRAS Proto-Oncogene, GTPase | NRAS |
| 496 | Myosin Heavy Chain 7 | MYH7 |
| 497 | Titin | TTN |
| 498 | Prolactin | PRL |
| 499 | MicroRNA 10a | MIR10A |
| 500 | Nuclear Factor Kappa B Subunit 1 | NFKB1 |
| 501 | Signal Transducer And Activator Of Transcription 3 | STAT3 |
| 502 | Matrix Metallopeptidase 7 | MMP7 |
| 503 | GC Vitamin D Binding Protein | GC |
| 504 | Chymotrypsin Like | CTRL |
| 505 | MicroRNA 206 | MIR206 |
| 506 | Dihydrofolate Reductase | DHFR |
| 507 | Iodothyronine Deiodinase 1 | DIO1 |
| 508 | Immediate Early Response 3 | IER3 |
| 509 | Energy Homeostasis Associated | ENHO |
| 510 | Placenta Enriched 4 | PLAC4 |
| 511 | Family With Sequence Similarity 99 Member A | FAM99A |
| 512 | Corticotropin-releasing factor receptor 1 | CRHR1 |
| 513 | CD9 antigen | CD9 |
| 514 | Pregnancy-specific beta-1-glycoprotein 10, pseudogene | PSG10P |
| 515 | Nuclear RNA export factor 5 | NXF5 |
| 516 | Pyruvate dehydrogenase E1 component subunit alpha, somatic form, mitochondrial | PDHA1 |
| 517 | Putative bifunctional UDP-N-acetylglucosamine transferase and deubiquitinase ALG13 | ALG13 |
| 518 | 15-hydroxyprostaglandin dehydrogenase [NAD | HPGD |
| 519 | Low-density lipoprotein receptor-related protein 8 | LRP8 |
| 520 | Syncytin-1 | ERVW1 |
| 521 | Killer cell immunoglobulin-like receptor 2DL5A | KIR2DL5A |
| 522 | Cytochrome P450 3A5 | CYP3A5 |
| 523 | WW domain-binding protein 1 | WBP1 |
| 524 | Catenin alpha-3 | CTNNA3 |
| 525 | INO80 complex subunit B | INO80B |
| 526 | Storkhead-box protein 2 | STOX2 |
