# Supplementary material for: A network pharmacology study on mechanism of resveratrol in treating preeclampsia via regulation of AGE-RAGE and HIF-1 signalling pathways
Source: Front Endocrinol (Lausanne). 2023 Jan 5;13:1044775. doi: 10.3389/fendo.2022.1044775 (PMC9849370; doi:10.3389/fendo.2022.1044775)
Supplement: Supplementary file 3 [file Table_3.docx]

**Additional file 3. The Intersecting Targets of RSV and PE**

| **No.** | **Target Name** | **Gene Symbol** |
| --- | --- | --- |
| 1 | Angiotensin I Converting Enzyme | ACE |
| 2 | Adiponectin Receptor 1 | ADIPOR1 |
| 3 | Angiotensin II Receptor Type 1 | AGTR1 |
| 4 | Aldo-Keto Reductase Family 1 Member C3 | AKR1C3 |
| 5 | Androgen Receptor | AR |
| 6 | BCL2 Apoptosis Regulator | BCL2 |
| 7 | Caspase 3 | CASP3 |
| 8 | Catalase | CAT |
| 9 | C-C Motif Chemokine Ligand 2 | CCL2 |
| 10 | C-Reactive Protein | CRP |
| 11 | C-X-C Motif Chemokine Ligand 12 | CXCL12 |
| 12 | C-X-C Motif Chemokine Ligand 8 | CXCL8 |
| 13 | Cytochrome P450 Family 11 Subfamily B Member 1 | CYP11B1 |
| 14 | Cytochrome P450 Family 11 Subfamily B Member 2 | CYP11B2 |
| 15 | Cytochrome P450 Family 17 Subfamily A Member 1 | CYP17A1 |
| 16 | Cytochrome P450 Family 1 Subfamily A Member 1 | CYP1A1 |
| 17 | Dopamine Receptor D2 | DRD2 |
| 18 | Endothelin 1 | EDN1 |
| 19 | Epidermal Growth Factor Receptor | EGFR |
| 20 | Estrogen Receptor 1 | ESR1 |
| 21 | Estrogen Receptor 2 | ESR2 |
| 22 | Coagulation Factor III, Tissue Factor | F3 |
| 23 | Hypoxia Inducible Factor 1 Subunit Alpha | HIF1A |
| 24 | Intercellular Adhesion Molecule 1 | ICAM1 |
| 25 | Interleukin 10 | IL10 |
| 26 | Interleukin 1 Alpha | IL1A |
| 27 | Interleukin 1 Beta | IL1B |
| 28 | Interleukin 6 | IL6 |
| 29 | Insulin Receptor | INSR |
| 30 | Insulin Receptor Substrate 1 | IRS1 |
| 31 | Integrin Subunit Beta 1 | ITGB1 |
| 32 | Mitogen-Activated Protein Kinase 1 | MAPK1 |
| 33 | Macrophage Migration Inhibitory Factor | MIF |
| 34 | Matrix Metallopeptidase 1 | MMP1 |
| 35 | Matrix Metallopeptidase 2 | MMP2 |
| 36 | Matrix Metallopeptidase 9 | MMP9 |
| 37 | Myeloperoxidase | MPO |
| 38 | Nuclear Factor, Erythroid 2 Like 2 | NFE2L2 |
| 39 | Nitric Oxide Synthase 2 | NOS2 |
| 40 | Nitric Oxide Synthase 3 | NOS3 |
| 41 | Nuclear Receptor Subfamily 1 Group H Member 3 | NR1H3 |
| 42 | Nuclear Receptor Subfamily 1 Group H Member 4 | NR1H4 |
| 43 | Pappalysin 1 | PAPPA |
| 44 | Platelet And Endothelial Cell Adhesion Molecule 1 | PECAM1 |
| 45 | Plasminogen Activator, Tissue Type | PLAT |
| 46 | Plasminogen Activator, Urokinase | PLAU |
| 47 | Paraoxonase 1 | PON1 |
| 48 | Peroxisome Proliferator Activated Receptor Gamma | PPARG |
| 49 | Phosphatase And Tensin Homolog | PTEN |
| 50 | Prostaglandin-Endoperoxide Synthase 2 | PTGS2 |
| 51 | RELA Proto-Oncogene, NF-KB Subunit | RELA |
| 52 | Selectin E | SELE |
| 53 | Serpin Family E Member 1 | SERPINE1 |
| 54 | Sex Hormone Binding Globulin | SHBG |
| 55 | Sirtuin 1 | SIRT1 |
| 56 | Superoxide Dismutase 1 | SOD1 |
| 57 | Superoxide Dismutase 2 | SOD2 |
| 58 | Signal Transducer And Activator Of Transcription 3 | STAT3 |
| 59 | Transforming Growth Factor Beta 1 | TGFB1 |
| 60 | Transforming Growth Factor Beta 2 | TGFB2 |
| 61 | Toll Like Receptor 9 | TLR9 |
| 62 | Tumor Necrosis Factor | TNF |
| 63 | Tumor Protein P53 | TP53 |
| 64 | Transthyretin | TTR |
| 65 | Vascular Cell Adhesion Molecule 1 | VCAM1 |
| 66 | Vascular Endothelial Growth Factor A | VEGFA |
