# Supplementary material for: A network pharmacology study on mechanism of resveratrol in treating preeclampsia via regulation of AGE-RAGE and HIF-1 signalling pathways
Source: Front Endocrinol (Lausanne). 2023 Jan 5;13:1044775. doi: 10.3389/fendo.2022.1044775 (PMC9849370; doi:10.3389/fendo.2022.1044775)
Supplement: Supplementary file 4 [file Table_4.docx]

**Additional file 4. The Results of GO Enrichment Analysis**

| **GO** | **Category** | **Description** | **LogP** |
| --- | --- | --- | --- |
| GO:0010035 | Biological Processes | response to inorganic substance | -29.4041 |
| GO:0000302 | Biological Processes | response to reactive oxygen species | -24.1682 |
| GO:0010038 | Biological Processes | response to metal ion | -22.9674 |
| GO:0007568 | Biological Processes | aging | -22.2417 |
| GO:0006979 | Biological Processes | response to oxidative stress | -21.2642 |
| GO:0042493 | Biological Processes | response to drug | -20.8293 |
| GO:0062197 | Biological Processes | cellular response to chemical stress | -20.6734 |
| GO:0031667 | Biological Processes | response to nutrient levels | -19.3869 |
| GO:0009991 | Biological Processes | response to extracellular stimulus | -18.8446 |
| GO:0034599 | Biological Processes | cellular response to oxidative stress | -18.7837 |
| GO:0034614 | Biological Processes | cellular response to reactive oxygen species | -18.1086 |
| GO:0071248 | Biological Processes | cellular response to metal ion | -11.9435 |
| GO:0071241 | Biological Processes | cellular response to inorganic substance | -11.294 |
| GO:0046686 | Biological Processes | response to cadmium ion | -9.61536 |
| GO:0071276 | Biological Processes | cellular response to cadmium ion | -7.42687 |
| GO:0030335 | Biological Processes | positive regulation of cell migration | -28.217 |
| GO:2000147 | Biological Processes | positive regulation of cell motility | -27.6838 |
| GO:0051272 | Biological Processes | positive regulation of cellular component movement | -27.4461 |
| GO:0040017 | Biological Processes | positive regulation of locomotion | -27.407 |
| GO:0001568 | Biological Processes | blood vessel development | -23.0372 |
| GO:0001525 | Biological Processes | angiogenesis | -21.454 |
| GO:0048514 | Biological Processes | blood vessel morphogenesis | -21.4099 |
| GO:0045765 | Biological Processes | regulation of angiogenesis | -17.4864 |
| GO:1901342 | Biological Processes | regulation of vasculature development | -17.3608 |
| GO:0045766 | Biological Processes | positive regulation of angiogenesis | -15.5031 |
| GO:1904018 | Biological Processes | positive regulation of vasculature development | -15.5031 |
| GO:0010631 | Biological Processes | epithelial cell migration | -14.3717 |
| GO:0090132 | Biological Processes | epithelium migration | -14.3186 |
| GO:0090130 | Biological Processes | tissue migration | -14.2312 |
| GO:0010632 | Biological Processes | regulation of epithelial cell migration | -14.2154 |
| GO:0001667 | Biological Processes | ameboidal-type cell migration | -12.5631 |
| GO:0043542 | Biological Processes | endothelial cell migration | -11.629 |
| GO:0010634 | Biological Processes | positive regulation of epithelial cell migration | -10.9356 |
| GO:0043534 | Biological Processes | blood vessel endothelial cell migration | -10.9356 |
| GO:0043535 | Biological Processes | regulation of blood vessel endothelial cell migration | -10.0594 |
| GO:0010594 | Biological Processes | regulation of endothelial cell migration | -9.75851 |
| GO:0043536 | Biological Processes | positive regulation of blood vessel endothelial cell migration | -9.22798 |
| GO:0010595 | Biological Processes | positive regulation of endothelial cell migration | -9.01346 |
| GO:0030099 | Biological Processes | myeloid cell differentiation | -6.56709 |
| GO:0030225 | Biological Processes | macrophage differentiation | -5.2688 |
| GO:0002573 | Biological Processes | myeloid leukocyte differentiation | -5.01082 |
| GO:0002237 | Biological Processes | response to molecule of bacterial origin | -28.1326 |
| GO:0032496 | Biological Processes | response to lipopolysaccharide | -27.0455 |
| GO:0009617 | Biological Processes | response to bacterium | -21.9894 |
| GO:0071396 | Biological Processes | cellular response to lipid | -21.8711 |
| GO:0071216 | Biological Processes | cellular response to biotic stimulus | -16.8369 |
| GO:0071222 | Biological Processes | cellular response to lipopolysaccharide | -16.2918 |
| GO:0071219 | Biological Processes | cellular response to molecule of bacterial origin | -15.949 |
| GO:0030155 | Biological Processes | regulation of cell adhesion | -15.4788 |
| GO:0019221 | Biological Processes | cytokine-mediated signaling pathway | -15.2379 |
| GO:0007159 | Biological Processes | leukocyte cell-cell adhesion | -12.81 |
| GO:0061756 | Biological Processes | leukocyte adhesion to vascular endothelial cell | -12.0528 |
| GO:0022407 | Biological Processes | regulation of cell-cell adhesion | -11.677 |
| GO:1903037 | Biological Processes | regulation of leukocyte cell-cell adhesion | -10.7229 |
| GO:0045785 | Biological Processes | positive regulation of cell adhesion | -10.6092 |
| GO:0070555 | Biological Processes | response to interleukin-1 | -10.2976 |
| GO:0071347 | Biological Processes | cellular response to interleukin-1 | -9.60652 |
| GO:1904994 | Biological Processes | regulation of leukocyte adhesion to vascular endothelial cell | -9.32471 |
| GO:0022409 | Biological Processes | positive regulation of cell-cell adhesion | -8.9237 |
| GO:1904996 | Biological Processes | positive regulation of leukocyte adhesion to vascular endothelial cell | -8.50799 |
| GO:1903039 | Biological Processes | positive regulation of leukocyte cell-cell adhesion | -8.32087 |
| GO:0001666 | Biological Processes | response to hypoxia | -26.4316 |
| GO:0036293 | Biological Processes | response to decreased oxygen levels | -25.9697 |
| GO:0070482 | Biological Processes | response to oxygen levels | -25.2482 |
| GO:2001233 | Biological Processes | regulation of apoptotic signaling pathway | -21.8162 |
| GO:0097190 | Biological Processes | apoptotic signaling pathway | -21.7524 |
| GO:2001234 | Biological Processes | negative regulation of apoptotic signaling pathway | -20.7055 |
| GO:1902532 | Biological Processes | negative regulation of intracellular signal transduction | -17.0228 |
| GO:0080135 | Biological Processes | regulation of cellular response to stress | -16.5153 |
| GO:0097193 | Biological Processes | intrinsic apoptotic signaling pathway | -12.8903 |
| GO:2001242 | Biological Processes | regulation of intrinsic apoptotic signaling pathway | -12.8189 |
| GO:2001243 | Biological Processes | negative regulation of intrinsic apoptotic signaling pathway | -11.7916 |
| GO:0071456 | Biological Processes | cellular response to hypoxia | -11.6248 |
| GO:0036294 | Biological Processes | cellular response to decreased oxygen levels | -11.3458 |
| GO:0071453 | Biological Processes | cellular response to oxygen levels | -10.9356 |
| GO:0071417 | Biological Processes | cellular response to organonitrogen compound | -23.6955 |
| GO:1901652 | Biological Processes | response to peptide | -23.0912 |
| GO:1901699 | Biological Processes | cellular response to nitrogen compound | -22.8139 |
| GO:0043434 | Biological Processes | response to peptide hormone | -18.9961 |
| GO:0032870 | Biological Processes | cellular response to hormone stimulus | -17.5226 |
| GO:1901653 | Biological Processes | cellular response to peptide | -17.1363 |
| GO:0071375 | Biological Processes | cellular response to peptide hormone stimulus | -14.2359 |
| GO:0042593 | Biological Processes | glucose homeostasis | -10.6368 |
| GO:0033500 | Biological Processes | carbohydrate homeostasis | -10.6189 |
| GO:0072593 | Biological Processes | reactive oxygen species metabolic process | -23.584 |
| GO:2000377 | Biological Processes | regulation of reactive oxygen species metabolic process | -16.318 |
| GO:0006801 | Biological Processes | superoxide metabolic process | -14.8056 |
| GO:2000379 | Biological Processes | positive regulation of reactive oxygen species metabolic process | -12.8174 |
| GO:0090322 | Biological Processes | regulation of superoxide metabolic process | -7.66563 |
| GO:0042554 | Biological Processes | superoxide anion generation | -7.2124 |
| GO:0032930 | Biological Processes | positive regulation of superoxide anion generation | -6.88741 |
| GO:0032928 | Biological Processes | regulation of superoxide anion generation | -6.5494 |
| GO:0009611 | Biological Processes | response to wounding | -22.2722 |
| GO:0042060 | Biological Processes | wound healing | -17.4086 |
| GO:1903034 | Biological Processes | regulation of response to wounding | -12.732 |
| GO:0050878 | Biological Processes | regulation of body fluid levels | -12.6376 |
| GO:0061041 | Biological Processes | regulation of wound healing | -12.1473 |
| GO:0030193 | Biological Processes | regulation of blood coagulation | -9.70887 |
| GO:1900046 | Biological Processes | regulation of hemostasis | -9.61536 |
| GO:0050818 | Biological Processes | regulation of coagulation | -9.48046 |
| GO:0061045 | Biological Processes | negative regulation of wound healing | -9.18794 |
| GO:1903035 | Biological Processes | negative regulation of response to wounding | -8.61248 |
| GO:0007596 | Biological Processes | blood coagulation | -7.35355 |
| GO:0007599 | Biological Processes | hemostasis | -7.27747 |
| GO:0050817 | Biological Processes | coagulation | -7.27747 |
| GO:0030195 | Biological Processes | negative regulation of blood coagulation | -6.97183 |
| GO:0014909 | Biological Processes | smooth muscle cell migration | -6.95927 |
| GO:1900047 | Biological Processes | negative regulation of hemostasis | -6.92686 |
| GO:0050819 | Biological Processes | negative regulation of coagulation | -6.79746 |
| GO:0014812 | Biological Processes | muscle cell migration | -6.63362 |
| GO:0033627 | Biological Processes | cell adhesion mediated by integrin | -6.12432 |
| GO:0014910 | Biological Processes | regulation of smooth muscle cell migration | -5.66538 |
| GO:0016485 | Biological Processes | protein processing | -4.8073 |
| GO:0031639 | Biological Processes | plasminogen activation | -4.61931 |
| GO:0042730 | Biological Processes | fibrinolysis | -4.56452 |
| GO:0051604 | Biological Processes | protein maturation | -4.16357 |
| GO:0033628 | Biological Processes | regulation of cell adhesion mediated by integrin | -3.70503 |
| GO:0031638 | Biological Processes | zymogen activation | -3.39624 |
| GO:0042476 | Biological Processes | odontogenesis | -2.43941 |
| GO:0048545 | Biological Processes | response to steroid hormone | -20.6734 |
| GO:0071407 | Biological Processes | cellular response to organic cyclic compound | -18.9143 |
| GO:0031960 | Biological Processes | response to corticosteroid | -12.732 |
| GO:0051384 | Biological Processes | response to glucocorticoid | -11.7124 |
| GO:1901654 | Biological Processes | response to ketone | -10.5852 |
| GO:0071383 | Biological Processes | cellular response to steroid hormone stimulus | -7.57692 |
| GO:1901655 | Biological Processes | cellular response to ketone | -5.54792 |
| GO:0071384 | Biological Processes | cellular response to corticosteroid stimulus | -4.8865 |
| GO:0071548 | Biological Processes | response to dexamethasone | -3.97554 |
| GO:0071385 | Biological Processes | cellular response to glucocorticoid stimulus | -3.50599 |
| GO:0051592 | Biological Processes | response to calcium ion | -2.27537 |
| GO:0003013 | Biological Processes | circulatory system process | -18.7191 |
| GO:0008015 | Biological Processes | blood circulation | -18.6772 |
| GO:0008217 | Biological Processes | regulation of blood pressure | -15.3478 |
| GO:0003018 | Biological Processes | vascular process in circulatory system | -13.3954 |
| GO:0035296 | Biological Processes | regulation of tube diameter | -11.9241 |
| GO:0097746 | Biological Processes | blood vessel diameter maintenance | -11.9241 |
| GO:0035150 | Biological Processes | regulation of tube size | -11.8932 |
| GO:0003073 | Biological Processes | regulation of systemic arterial blood pressure | -10.1795 |
| GO:0090066 | Biological Processes | regulation of anatomical structure size | -9.69912 |
| GO:0044057 | Biological Processes | regulation of system process | -9.09241 |
| GO:0042310 | Biological Processes | vasoconstriction | -8.9586 |
| GO:0003044 | Biological Processes | regulation of systemic arterial blood pressure mediated by a chemical signal | -8.88351 |
| GO:0019229 | Biological Processes | regulation of vasoconstriction | -7.97113 |
| GO:0001990 | Biological Processes | regulation of systemic arterial blood pressure by hormone | -7.60336 |
| GO:0050886 | Biological Processes | endocrine process | -7.36613 |
| GO:1903522 | Biological Processes | regulation of blood circulation | -6.80419 |
| GO:0050900 | Biological Processes | leukocyte migration | -18.417 |
| GO:0002685 | Biological Processes | regulation of leukocyte migration | -13.0783 |
| GO:0060326 | Biological Processes | cell chemotaxis | -12.4475 |
| GO:0006935 | Biological Processes | chemotaxis | -12.2487 |
| GO:0042330 | Biological Processes | taxis | -12.2162 |
| GO:0097529 | Biological Processes | myeloid leukocyte migration | -11.357 |
| GO:0050920 | Biological Processes | regulation of chemotaxis | -11.3359 |
| GO:0030595 | Biological Processes | leukocyte chemotaxis | -11.1707 |
| GO:0002688 | Biological Processes | regulation of leukocyte chemotaxis | -10.8891 |
| GO:0002687 | Biological Processes | positive regulation of leukocyte migration | -10.523 |
| GO:0050921 | Biological Processes | positive regulation of chemotaxis | -10.3526 |
| GO:0002690 | Biological Processes | positive regulation of leukocyte chemotaxis | -8.61248 |
| GO:0071674 | Biological Processes | mononuclear cell migration | -7.71203 |
| GO:0071675 | Biological Processes | regulation of mononuclear cell migration | -5.13635 |
| GO:0002548 | Biological Processes | monocyte chemotaxis | -4.64861 |
| GO:0071677 | Biological Processes | positive regulation of mononuclear cell migration | -3.31504 |
| GO:0050673 | Biological Processes | epithelial cell proliferation | -18.3577 |
| GO:0050678 | Biological Processes | regulation of epithelial cell proliferation | -15.1769 |
| GO:0001936 | Biological Processes | regulation of endothelial cell proliferation | -12.2425 |
| GO:0001935 | Biological Processes | endothelial cell proliferation | -11.8717 |
| GO:0050679 | Biological Processes | positive regulation of epithelial cell proliferation | -10.2216 |
| GO:0051345 | Biological Processes | positive regulation of hydrolase activity | -10.0862 |
| GO:0001938 | Biological Processes | positive regulation of endothelial cell proliferation | -9.669 |
| GO:0001934 | Biological Processes | positive regulation of protein phosphorylation | -18.2137 |
| GO:0043408 | Biological Processes | regulation of MAPK cascade | -15.1251 |
| GO:0043410 | Biological Processes | positive regulation of MAPK cascade | -15.1025 |
| GO:0000165 | Biological Processes | MAPK cascade | -14.0294 |
| GO:0045859 | Biological Processes | regulation of protein kinase activity | -11.8497 |
| GO:0043549 | Biological Processes | regulation of kinase activity | -11.8459 |
| GO:0051347 | Biological Processes | positive regulation of transferase activity | -11.5458 |
| GO:0071900 | Biological Processes | regulation of protein serine/threonine kinase activity | -10.2933 |
| GO:0033674 | Biological Processes | positive regulation of kinase activity | -10.1053 |
| GO:0045860 | Biological Processes | positive regulation of protein kinase activity | -9.86239 |
| GO:0043406 | Biological Processes | positive regulation of MAP kinase activity | -9.57572 |
| GO:0043405 | Biological Processes | regulation of MAP kinase activity | -9.38283 |
| GO:0071902 | Biological Processes | positive regulation of protein serine/threonine kinase activity | -8.90479 |
| GO:1901222 | Biological Processes | regulation of NIK/NF-kappaB signaling | -7.99774 |
| GO:1901224 | Biological Processes | positive regulation of NIK/NF-kappaB signaling | -7.85314 |
| GO:0038061 | Biological Processes | NIK/NF-kappaB signaling | -7.27944 |
| GO:0032872 | Biological Processes | regulation of stress-activated MAPK cascade | -6.4377 |
| GO:0070302 | Biological Processes | regulation of stress-activated protein kinase signaling cascade | -6.39271 |
| GO:0051403 | Biological Processes | stress-activated MAPK cascade | -5.81831 |
| GO:0031098 | Biological Processes | stress-activated protein kinase signaling cascade | -5.72376 |
| GO:0043122 | Biological Processes | regulation of I-kappaB kinase/NF-kappaB signaling | -5.71217 |
| GO:0007249 | Biological Processes | I-kappaB kinase/NF-kappaB signaling | -5.3659 |
| GO:0032874 | Biological Processes | positive regulation of stress-activated MAPK cascade | -4.94149 |
| GO:0070304 | Biological Processes | positive regulation of stress-activated protein kinase signaling cascade | -4.90803 |
| GO:0046328 | Biological Processes | regulation of JNK cascade | -3.56352 |
| GO:0007254 | Biological Processes | JNK cascade | -3.18987 |
| GO:0032729 | Biological Processes | positive regulation of interferon-gamma production | -3.18486 |
| GO:0030324 | Biological Processes | lung development | -3.09554 |
| GO:0030323 | Biological Processes | respiratory tube development | -3.05943 |
| GO:0046330 | Biological Processes | positive regulation of JNK cascade | -2.93164 |
| GO:0060541 | Biological Processes | respiratory system development | -2.86744 |
| GO:0001819 | Biological Processes | positive regulation of cytokine production | -18.0574 |
| GO:0001817 | Biological Processes | regulation of cytokine production | -16.1734 |
| GO:0032677 | Biological Processes | regulation of interleukin-8 production | -15.133 |
| GO:0032637 | Biological Processes | interleukin-8 production | -15.0848 |
| GO:0032757 | Biological Processes | positive regulation of interleukin-8 production | -13.6528 |
| GO:0002252 | Biological Processes | immune effector process | -10.608 |
| GO:0097696 | Biological Processes | receptor signaling pathway via STAT | -9.40419 |
| GO:0032755 | Biological Processes | positive regulation of interleukin-6 production | -8.6453 |
| GO:0002718 | Biological Processes | regulation of cytokine production involved in immune response | -8.51622 |
| GO:0002367 | Biological Processes | cytokine production involved in immune response | -8.48484 |
| GO:0032635 | Biological Processes | interleukin-6 production | -8.31827 |
| GO:0032675 | Biological Processes | regulation of interleukin-6 production | -8.31827 |
| GO:0002700 | Biological Processes | regulation of production of molecular mediator of immune response | -8.27683 |
| GO:0002697 | Biological Processes | regulation of immune effector process | -8.16264 |
| GO:0002699 | Biological Processes | positive regulation of immune effector process | -7.07379 |
| GO:1904894 | Biological Processes | positive regulation of receptor signaling pathway via STAT | -6.97183 |
| GO:0032642 | Biological Processes | regulation of chemokine production | -6.95927 |
| GO:0032602 | Biological Processes | chemokine production | -6.93262 |
| GO:0007259 | Biological Processes | receptor signaling pathway via JAK-STAT | -6.8443 |
| GO:1904892 | Biological Processes | regulation of receptor signaling pathway via STAT | -6.58716 |
| GO:0034113 | Biological Processes | heterotypic cell-cell adhesion | -6.45138 |
| GO:0002702 | Biological Processes | positive regulation of production of molecular mediator of immune response | -6.43121 |
| GO:0002719 | Biological Processes | negative regulation of cytokine production involved in immune response | -6.33379 |
| GO:0002440 | Biological Processes | production of molecular mediator of immune response | -6.19736 |
| GO:0032722 | Biological Processes | positive regulation of chemokine production | -6.18572 |
| GO:0002637 | Biological Processes | regulation of immunoglobulin production | -6.06469 |
| GO:0002443 | Biological Processes | leukocyte mediated immunity | -6.04853 |
| GO:0002703 | Biological Processes | regulation of leukocyte mediated immunity | -5.97946 |
| GO:0042742 | Biological Processes | defense response to bacterium | -5.75584 |
| GO:0002460 | Biological Processes | adaptive immune response based on somatic recombination of immune receptors built from immunoglobulin superfamily domains | -5.72862 |
| GO:0002701 | Biological Processes | negative regulation of production of molecular mediator of immune response | -5.6721 |
| GO:0046427 | Biological Processes | positive regulation of receptor signaling pathway via JAK-STAT | -5.45832 |
| GO:0002819 | Biological Processes | regulation of adaptive immune response | -5.3409 |
| GO:0042116 | Biological Processes | macrophage activation | -5.31124 |
| GO:0002639 | Biological Processes | positive regulation of immunoglobulin production | -5.19865 |
| GO:0002821 | Biological Processes | positive regulation of adaptive immune response | -5.17391 |
| GO:0032609 | Biological Processes | interferon-gamma production | -5.17391 |
| GO:0032649 | Biological Processes | regulation of interferon-gamma production | -5.17391 |
| GO:0034116 | Biological Processes | positive regulation of heterotypic cell-cell adhesion | -5.1715 |
| GO:0045599 | Biological Processes | negative regulation of fat cell differentiation | -5.03522 |
| GO:0002544 | Biological Processes | chronic inflammatory response | -5.00946 |
| GO:0033619 | Biological Processes | membrane protein proteolysis | -5.00439 |
| GO:0043030 | Biological Processes | regulation of macrophage activation | -4.91519 |
| GO:0002274 | Biological Processes | myeloid leukocyte activation | -4.82903 |
| GO:0002705 | Biological Processes | positive regulation of leukocyte mediated immunity | -4.77956 |
| GO:0002449 | Biological Processes | lymphocyte mediated immunity | -4.73893 |
| GO:0001818 | Biological Processes | negative regulation of cytokine production | -4.67621 |
| GO:0051043 | Biological Processes | regulation of membrane protein ectodomain proteolysis | -4.61931 |
| GO:0034114 | Biological Processes | regulation of heterotypic cell-cell adhesion | -4.46158 |
| GO:0002706 | Biological Processes | regulation of lymphocyte mediated immunity | -4.32405 |
| GO:0002822 | Biological Processes | regulation of adaptive immune response based on somatic recombination of immune receptors built from immunoglobulin superfamily domains | -4.32405 |
| GO:0002250 | Biological Processes | adaptive immune response | -4.30301 |
| GO:0007435 | Biological Processes | salivary gland morphogenesis | -4.23586 |
| GO:0050829 | Biological Processes | defense response to Gram-negative bacterium | -4.2 |
| GO:0007431 | Biological Processes | salivary gland development | -4.15581 |
| GO:0046425 | Biological Processes | regulation of receptor signaling pathway via JAK-STAT | -4.0236 |
| GO:0002824 | Biological Processes | positive regulation of adaptive immune response based on somatic recombination of immune receptors built from immunoglobulin superfamily domains | -3.92645 |
| GO:0002698 | Biological Processes | negative regulation of immune effector process | -3.88002 |
| GO:0002708 | Biological Processes | positive regulation of lymphocyte mediated immunity | -3.83492 |
| GO:0002377 | Biological Processes | immunoglobulin production | -3.80065 |
| GO:0035272 | Biological Processes | exocrine system development | -3.78881 |
| GO:0006509 | Biological Processes | membrane protein ectodomain proteolysis | -3.76025 |
| GO:0002712 | Biological Processes | regulation of B cell mediated immunity | -3.41741 |
| GO:0002889 | Biological Processes | regulation of immunoglobulin mediated immune response | -3.41741 |
| GO:0032715 | Biological Processes | negative regulation of interleukin-6 production | -3.31504 |
| GO:0002437 | Biological Processes | inflammatory response to antigenic stimulus | -3.15011 |
| GO:0002312 | Biological Processes | B cell activation involved in immune response | -3.02041 |
| GO:0016064 | Biological Processes | immunoglobulin mediated immune response | -2.84416 |
| GO:0019724 | Biological Processes | B cell mediated immunity | -2.82126 |
| GO:1902107 | Biological Processes | positive regulation of leukocyte differentiation | -2.20543 |
| GO:1903708 | Biological Processes | positive regulation of hemopoiesis | -2.20543 |
| GO:0051091 | Biological Processes | positive regulation of DNA-binding transcription factor activity | -18.003 |
| GO:0051090 | Biological Processes | regulation of DNA-binding transcription factor activity | -15.7424 |
| GO:0051092 | Biological Processes | positive regulation of NF-kappaB transcription factor activity | -10.0339 |
| GO:0062012 | Biological Processes | regulation of small molecule metabolic process | -17.7228 |
| GO:0062013 | Biological Processes | positive regulation of small molecule metabolic process | -16.857 |
| GO:0032787 | Biological Processes | monocarboxylic acid metabolic process | -12.3804 |
| GO:0019216 | Biological Processes | regulation of lipid metabolic process | -12.1552 |
| GO:0042180 | Biological Processes | cellular ketone metabolic process | -11.6859 |
| GO:0044283 | Biological Processes | small molecule biosynthetic process | -10.4749 |
| GO:0008610 | Biological Processes | lipid biosynthetic process | -10.2746 |
| GO:0045834 | Biological Processes | positive regulation of lipid metabolic process | -10.1371 |
| GO:0001516 | Biological Processes | prostaglandin biosynthetic process | -10.0347 |
| GO:0046457 | Biological Processes | prostanoid biosynthetic process | -10.0347 |
| GO:0070542 | Biological Processes | response to fatty acid | -9.80545 |
| GO:0010565 | Biological Processes | regulation of cellular ketone metabolic process | -9.03939 |
| GO:0006692 | Biological Processes | prostanoid metabolic process | -8.77038 |
| GO:0006693 | Biological Processes | prostaglandin metabolic process | -8.77038 |
| GO:0006631 | Biological Processes | fatty acid metabolic process | -8.77034 |
| GO:0072330 | Biological Processes | monocarboxylic acid biosynthetic process | -8.7407 |
| GO:0006636 | Biological Processes | unsaturated fatty acid biosynthetic process | -8.66209 |
| GO:0046456 | Biological Processes | icosanoid biosynthetic process | -8.4101 |
| GO:0006633 | Biological Processes | fatty acid biosynthetic process | -8.33919 |
| GO:0033559 | Biological Processes | unsaturated fatty acid metabolic process | -7.97151 |
| GO:0006690 | Biological Processes | icosanoid metabolic process | -7.79431 |
| GO:0045923 | Biological Processes | positive regulation of fatty acid metabolic process | -7.48407 |
| GO:0046394 | Biological Processes | carboxylic acid biosynthetic process | -7.28595 |
| GO:0016053 | Biological Processes | organic acid biosynthetic process | -7.26248 |
| GO:0019217 | Biological Processes | regulation of fatty acid metabolic process | -7.06883 |
| GO:0071398 | Biological Processes | cellular response to fatty acid | -5.71833 |
| GO:0010817 | Biological Processes | regulation of hormone levels | -17.4103 |
| GO:0050727 | Biological Processes | regulation of inflammatory response | -16.6331 |
| GO:0031347 | Biological Processes | regulation of defense response | -14.467 |
| GO:0050708 | Biological Processes | regulation of protein secretion | -13.2904 |
| GO:0032103 | Biological Processes | positive regulation of response to external stimulus | -13.2716 |
| GO:1903530 | Biological Processes | regulation of secretion by cell | -12.7225 |
| GO:0051223 | Biological Processes | regulation of protein transport | -12.1102 |
| GO:0051046 | Biological Processes | regulation of secretion | -12.0775 |
| GO:0070201 | Biological Processes | regulation of establishment of protein localization | -11.8153 |
| GO:0009306 | Biological Processes | protein secretion | -11.6767 |
| GO:0035592 | Biological Processes | establishment of protein localization to extracellular region | -11.6614 |
| GO:0071692 | Biological Processes | protein localization to extracellular region | -11.5562 |
| GO:0042063 | Biological Processes | gliogenesis | -11.2792 |
| GO:0009914 | Biological Processes | hormone transport | -11.1462 |
| GO:0001503 | Biological Processes | ossification | -10.9406 |
| GO:0046883 | Biological Processes | regulation of hormone secretion | -10.7829 |
| GO:0032940 | Biological Processes | secretion by cell | -10.4925 |
| GO:0050767 | Biological Processes | regulation of neurogenesis | -10.3207 |
| GO:0033135 | Biological Processes | regulation of peptidyl-serine phosphorylation | -10.2703 |
| GO:0010720 | Biological Processes | positive regulation of cell development | -10.0029 |
| GO:0046879 | Biological Processes | hormone secretion | -10.0029 |
| GO:0050769 | Biological Processes | positive regulation of neurogenesis | -9.907 |
| GO:0060284 | Biological Processes | regulation of cell development | -9.86607 |
| GO:0033138 | Biological Processes | positive regulation of peptidyl-serine phosphorylation | -9.76499 |
| GO:0030072 | Biological Processes | peptide hormone secretion | -9.70422 |
| GO:0002790 | Biological Processes | peptide secretion | -9.59781 |
| GO:0051960 | Biological Processes | regulation of nervous system development | -9.34412 |
| GO:0015833 | Biological Processes | peptide transport | -9.23039 |
| GO:0023061 | Biological Processes | signal release | -9.10552 |
| GO:0051962 | Biological Processes | positive regulation of nervous system development | -9.10483 |
| GO:0090276 | Biological Processes | regulation of peptide hormone secretion | -9.07673 |
| GO:0002791 | Biological Processes | regulation of peptide secretion | -8.9993 |
| GO:0090087 | Biological Processes | regulation of peptide transport | -8.9612 |
| GO:0050729 | Biological Processes | positive regulation of inflammatory response | -8.81306 |
| GO:0042886 | Biological Processes | amide transport | -8.63932 |
| GO:0010001 | Biological Processes | glial cell differentiation | -8.54986 |
| GO:0018105 | Biological Processes | peptidyl-serine phosphorylation | -8.49135 |
| GO:0050796 | Biological Processes | regulation of insulin secretion | -8.29748 |
| GO:0018209 | Biological Processes | peptidyl-serine modification | -8.19916 |
| GO:0014015 | Biological Processes | positive regulation of gliogenesis | -7.97113 |
| GO:0030073 | Biological Processes | insulin secretion | -7.72933 |
| GO:0060252 | Biological Processes | positive regulation of glial cell proliferation | -6.88741 |
| GO:0014013 | Biological Processes | regulation of gliogenesis | -6.80357 |
| GO:0031349 | Biological Processes | positive regulation of defense response | -6.55629 |
| GO:0060251 | Biological Processes | regulation of glial cell proliferation | -5.81479 |
| GO:0014009 | Biological Processes | glial cell proliferation | -5.19865 |
| GO:0021782 | Biological Processes | glial cell development | -5.09947 |
| GO:0048143 | Biological Processes | astrocyte activation | -4.56452 |
| GO:0014002 | Biological Processes | astrocyte development | -3.84799 |
| GO:0070848 | Biological Processes | response to growth factor | -17.082 |
| GO:0071363 | Biological Processes | cellular response to growth factor stimulus | -16.1944 |
| GO:0007507 | Biological Processes | heart development | -10.067 |
| GO:0048598 | Biological Processes | embryonic morphogenesis | -8.96737 |
| GO:0048863 | Biological Processes | stem cell differentiation | -8.86768 |
| GO:0048568 | Biological Processes | embryonic organ development | -8.29824 |
| GO:0043009 | Biological Processes | chordate embryonic development | -6.76264 |
| GO:0009792 | Biological Processes | embryo development ending in birth or egg hatching | -6.62377 |
| GO:0003007 | Biological Processes | heart morphogenesis | -5.68915 |
| GO:0001701 | Biological Processes | in utero embryonic development | -5.62221 |
| GO:0009314 | Biological Processes | response to radiation | -16.8345 |
| GO:0009416 | Biological Processes | response to light stimulus | -13.6694 |
| GO:0009411 | Biological Processes | response to UV | -13.2485 |
| GO:0032963 | Biological Processes | collagen metabolic process | -8.30336 |
| GO:0071214 | Biological Processes | cellular response to abiotic stimulus | -8.26082 |
| GO:0104004 | Biological Processes | cellular response to environmental stimulus | -8.26082 |
| GO:0070141 | Biological Processes | response to UV-A | -7.56768 |
| GO:0034644 | Biological Processes | cellular response to UV | -7.1255 |
| GO:0022617 | Biological Processes | extracellular matrix disassembly | -6.34772 |
| GO:0071482 | Biological Processes | cellular response to light stimulus | -6.32565 |
| GO:0030198 | Biological Processes | extracellular matrix organization | -6.27232 |
| GO:0043062 | Biological Processes | extracellular structure organization | -6.2615 |
| GO:0045229 | Biological Processes | external encapsulating structure organization | -6.23997 |
| GO:0022411 | Biological Processes | cellular component disassembly | -6.00012 |
| GO:0071492 | Biological Processes | cellular response to UV-A | -5.69856 |
| GO:0030574 | Biological Processes | collagen catabolic process | -5.54059 |
| GO:0071478 | Biological Processes | cellular response to radiation | -5.28695 |
| GO:1904645 | Biological Processes | response to amyloid-beta | -4.94439 |
| GO:0007369 | Biological Processes | gastrulation | -4.11456 |
| GO:0001704 | Biological Processes | formation of primary germ layer | -2.53649 |
| GO:0033002 | Biological Processes | muscle cell proliferation | -16.7575 |
| GO:0008285 | Biological Processes | negative regulation of cell population proliferation | -16.394 |
| GO:0043068 | Biological Processes | positive regulation of programmed cell death | -14.083 |
| GO:0048660 | Biological Processes | regulation of smooth muscle cell proliferation | -13.9314 |
| GO:0048659 | Biological Processes | smooth muscle cell proliferation | -13.8163 |
| GO:0010942 | Biological Processes | positive regulation of cell death | -13.3238 |
| GO:0043065 | Biological Processes | positive regulation of apoptotic process | -13.0352 |
| GO:0048661 | Biological Processes | positive regulation of smooth muscle cell proliferation | -11.5544 |
| GO:1904705 | Biological Processes | regulation of vascular associated smooth muscle cell proliferation | -10.4078 |
| GO:1990874 | Biological Processes | vascular associated smooth muscle cell proliferation | -10.3687 |
| GO:1904707 | Biological Processes | positive regulation of vascular associated smooth muscle cell proliferation | -6.97183 |
| GO:0040008 | Biological Processes | regulation of growth | -16.5406 |
| GO:0016049 | Biological Processes | cell growth | -10.0499 |
| GO:0001558 | Biological Processes | regulation of cell growth | -9.64325 |
| GO:0035051 | Biological Processes | cardiocyte differentiation | -8.46766 |
| GO:0045926 | Biological Processes | negative regulation of growth | -8.16591 |
| GO:0030308 | Biological Processes | negative regulation of cell growth | -6.52996 |
| GO:0051149 | Biological Processes | positive regulation of muscle cell differentiation | -5.86863 |
| GO:0048738 | Biological Processes | cardiac muscle tissue development | -5.85465 |
| GO:0061061 | Biological Processes | muscle structure development | -5.66235 |
| GO:0014706 | Biological Processes | striated muscle tissue development | -5.49444 |
| GO:0042692 | Biological Processes | muscle cell differentiation | -5.49444 |
| GO:0060537 | Biological Processes | muscle tissue development | -5.3403 |
| GO:0051147 | Biological Processes | regulation of muscle cell differentiation | -4.46434 |
| GO:0051146 | Biological Processes | striated muscle cell differentiation | -4.25441 |
| GO:0055007 | Biological Processes | cardiac muscle cell differentiation | -3.66648 |
| GO:0051155 | Biological Processes | positive regulation of striated muscle cell differentiation | -3.48324 |
| GO:0055001 | Biological Processes | muscle cell development | -3.05054 |
| GO:0051153 | Biological Processes | regulation of striated muscle cell differentiation | -2.75959 |
| GO:0061448 | Biological Processes | connective tissue development | -2.51584 |
| GO:0048871 | Biological Processes | multicellular organismal homeostasis | -15.9841 |
| GO:0060249 | Biological Processes | anatomical structure homeostasis | -9.76139 |
| GO:0001894 | Biological Processes | tissue homeostasis | -9.16711 |
| GO:0035633 | Biological Processes | maintenance of blood-brain barrier | -7.72979 |
| GO:0002040 | Biological Processes | sprouting angiogenesis | -4.09264 |
| GO:0002042 | Biological Processes | cell migration involved in sprouting angiogenesis | -2.84909 |
| GO:0048732 | Biological Processes | gland development | -15.7424 |
| GO:0030855 | Biological Processes | epithelial cell differentiation | -10.73 |
| GO:0048729 | Biological Processes | tissue morphogenesis | -9.8514 |
| GO:0030522 | Biological Processes | intracellular receptor signaling pathway | -9.19862 |
| GO:0002009 | Biological Processes | morphogenesis of an epithelium | -8.66315 |
| GO:0002064 | Biological Processes | epithelial cell development | -8.54986 |
| GO:0060562 | Biological Processes | epithelial tube morphogenesis | -8.29829 |
| GO:0022612 | Biological Processes | gland morphogenesis | -7.86891 |
| GO:0001763 | Biological Processes | morphogenesis of a branching structure | -7.69482 |
| GO:0060749 | Biological Processes | mammary gland alveolus development | -7.08546 |
| GO:0061377 | Biological Processes | mammary gland lobule development | -7.08546 |
| GO:0061138 | Biological Processes | morphogenesis of a branching epithelium | -6.60928 |
| GO:0060736 | Biological Processes | prostate gland growth | -5.83614 |
| GO:0048754 | Biological Processes | branching morphogenesis of an epithelial tube | -5.80738 |
| GO:0009755 | Biological Processes | hormone-mediated signaling pathway | -5.24729 |
| GO:0060688 | Biological Processes | regulation of morphogenesis of a branching structure | -5.23335 |
| GO:0060135 | Biological Processes | maternal process involved in female pregnancy | -4.8583 |
| GO:1905330 | Biological Processes | regulation of morphogenesis of an epithelium | -4.77649 |
| GO:0030879 | Biological Processes | mammary gland development | -4.70333 |
| GO:0061180 | Biological Processes | mammary gland epithelium development | -4.64861 |
| GO:0030518 | Biological Processes | intracellular steroid hormone receptor signaling pathway | -3.79108 |
| GO:0030850 | Biological Processes | prostate gland development | -3.78881 |
| GO:2000027 | Biological Processes | regulation of animal organ morphogenesis | -3.66648 |
| GO:0030520 | Biological Processes | intracellular estrogen receptor signaling pathway | -3.55281 |
| GO:0043401 | Biological Processes | steroid hormone mediated signaling pathway | -3.52662 |
| GO:2000241 | Biological Processes | regulation of reproductive process | -3.16094 |
| GO:0043627 | Biological Processes | response to estrogen | -3.13311 |
| GO:0007169 | Biological Processes | transmembrane receptor protein tyrosine kinase signaling pathway | -15.6717 |
| GO:1902895 | Biological Processes | positive regulation of pri-miRNA transcription by RNA polymerase II | -15.2949 |
| GO:0061614 | Biological Processes | pri-miRNA transcription by RNA polymerase II | -14.1511 |
| GO:1902893 | Biological Processes | regulation of pri-miRNA transcription by RNA polymerase II | -14.1511 |
| GO:0060965 | Biological Processes | negative regulation of gene silencing by miRNA | -13.3334 |
| GO:0060149 | Biological Processes | negative regulation of posttranscriptional gene silencing | -13.0276 |
| GO:0060967 | Biological Processes | negative regulation of gene silencing by RNA | -13.0276 |
| GO:1903798 | Biological Processes | regulation of production of miRNAs involved in gene silencing by miRNA | -12.8857 |
| GO:0070920 | Biological Processes | regulation of production of small RNA involved in gene silencing by RNA | -12.7503 |
| GO:0060964 | Biological Processes | regulation of gene silencing by miRNA | -12.6104 |
| GO:0060147 | Biological Processes | regulation of posttranscriptional gene silencing | -12.3904 |
| GO:0060966 | Biological Processes | regulation of gene silencing by RNA | -12.3202 |
| GO:1903799 | Biological Processes | negative regulation of production of miRNAs involved in gene silencing by miRNA | -12.1896 |
| GO:0060969 | Biological Processes | negative regulation of gene silencing | -11.4769 |
| GO:0060968 | Biological Processes | regulation of gene silencing | -10.7389 |
| GO:0035196 | Biological Processes | production of miRNAs involved in gene silencing by miRNA | -10.4627 |
| GO:0031050 | Biological Processes | dsRNA processing | -10.3425 |
| GO:0070918 | Biological Processes | production of small RNA involved in gene silencing by RNA | -10.3425 |
| GO:2000637 | Biological Processes | positive regulation of gene silencing by miRNA | -8.08342 |
| GO:0060148 | Biological Processes | positive regulation of posttranscriptional gene silencing | -8.00782 |
| GO:1903800 | Biological Processes | positive regulation of production of miRNAs involved in gene silencing by miRNA | -7.87198 |
| GO:0002532 | Biological Processes | production of molecular mediator involved in inflammatory response | -6.93262 |
| GO:1900017 | Biological Processes | positive regulation of cytokine production involved in inflammatory response | -6.40266 |
| GO:0002360 | Biological Processes | T cell lineage commitment | -6.20391 |
| GO:0002534 | Biological Processes | cytokine production involved in inflammatory response | -6.12432 |
| GO:1900015 | Biological Processes | regulation of cytokine production involved in inflammatory response | -6.12432 |
| GO:0048708 | Biological Processes | astrocyte differentiation | -5.86863 |
| GO:0071354 | Biological Processes | cellular response to interleukin-6 | -5.41866 |
| GO:0070741 | Biological Processes | response to interleukin-6 | -5.2688 |
| GO:0002285 | Biological Processes | lymphocyte activation involved in immune response | -5.19546 |
| GO:0002363 | Biological Processes | alpha-beta T cell lineage commitment | -4.67657 |
| GO:0043369 | Biological Processes | CD4-positive or CD8-positive, alpha-beta T cell lineage commitment | -4.67657 |
| GO:0051052 | Biological Processes | regulation of DNA metabolic process | -4.66077 |
| GO:0002366 | Biological Processes | leukocyte activation involved in immune response | -4.33165 |
| GO:0002263 | Biological Processes | cell activation involved in immune response | -4.29698 |
| GO:0034103 | Biological Processes | regulation of tissue remodeling | -4.27639 |
| GO:0016458 | Biological Processes | gene silencing | -4.09111 |
| GO:0043368 | Biological Processes | positive T cell selection | -4.04456 |
| GO:0045639 | Biological Processes | positive regulation of myeloid cell differentiation | -3.97428 |
| GO:0051054 | Biological Processes | positive regulation of DNA metabolic process | -3.94599 |
| GO:0032733 | Biological Processes | positive regulation of interleukin-10 production | -3.91013 |
| GO:0035195 | Biological Processes | gene silencing by miRNA | -3.87891 |
| GO:0035194 | Biological Processes | post-transcriptional gene silencing by RNA | -3.84691 |
| GO:0002286 | Biological Processes | T cell activation involved in immune response | -3.83492 |
| GO:0016441 | Biological Processes | posttranscriptional gene silencing | -3.82881 |
| GO:0031047 | Biological Processes | gene silencing by RNA | -3.72286 |
| GO:0045058 | Biological Processes | T cell selection | -3.65217 |
| GO:0032612 | Biological Processes | interleukin-1 production | -3.61414 |
| GO:0032652 | Biological Processes | regulation of interleukin-1 production | -3.61414 |
| GO:0032613 | Biological Processes | interleukin-10 production | -3.37542 |
| GO:0032653 | Biological Processes | regulation of interleukin-10 production | -3.37542 |
| GO:0032731 | Biological Processes | positive regulation of interleukin-1 beta production | -3.37542 |
| GO:0032732 | Biological Processes | positive regulation of interleukin-1 production | -3.16736 |
| GO:0046849 | Biological Processes | bone remodeling | -2.88964 |
| GO:0045637 | Biological Processes | regulation of myeloid cell differentiation | -2.81371 |
| GO:0032611 | Biological Processes | interleukin-1 beta production | -2.64264 |
| GO:0032651 | Biological Processes | regulation of interleukin-1 beta production | -2.64264 |
| GO:0046632 | Biological Processes | alpha-beta T cell differentiation | -2.64264 |
| GO:0045727 | Biological Processes | positive regulation of translation | -2.39385 |
| GO:0046631 | Biological Processes | alpha-beta T cell activation | -2.23597 |
| GO:0043043 | Biological Processes | peptide biosynthetic process | -2.0563 |
| GO:0034612 | Biological Processes | response to tumor necrosis factor | -15.0997 |
| GO:0071356 | Biological Processes | cellular response to tumor necrosis factor | -11.2319 |
| GO:0071496 | Biological Processes | cellular response to external stimulus | -11.0008 |
| GO:0031668 | Biological Processes | cellular response to extracellular stimulus | -8.24254 |
| GO:0031669 | Biological Processes | cellular response to nutrient levels | -7.43152 |
| GO:0009267 | Biological Processes | cellular response to starvation | -5.74175 |
| GO:0042594 | Biological Processes | response to starvation | -5.15733 |
| GO:0034976 | Biological Processes | response to endoplasmic reticulum stress | -4.51369 |
| GO:0045596 | Biological Processes | negative regulation of cell differentiation | -15.0205 |
| GO:0048511 | Biological Processes | rhythmic process | -9.95657 |
| GO:0045936 | Biological Processes | negative regulation of phosphate metabolic process | -8.21604 |
| GO:0010563 | Biological Processes | negative regulation of phosphorus metabolic process | -8.20588 |
| GO:0051129 | Biological Processes | negative regulation of cellular component organization | -7.78167 |
| GO:0043086 | Biological Processes | negative regulation of catalytic activity | -7.50973 |
| GO:0007623 | Biological Processes | circadian rhythm | -7.44734 |
| GO:0042326 | Biological Processes | negative regulation of phosphorylation | -5.48612 |
| GO:0031400 | Biological Processes | negative regulation of protein modification process | -5.48366 |
| GO:0071901 | Biological Processes | negative regulation of protein serine/threonine kinase activity | -5.02771 |
| GO:0001933 | Biological Processes | negative regulation of protein phosphorylation | -4.79513 |
| GO:0051348 | Biological Processes | negative regulation of transferase activity | -4.40267 |
| GO:0006469 | Biological Processes | negative regulation of protein kinase activity | -3.83831 |
| GO:0033673 | Biological Processes | negative regulation of kinase activity | -3.63159 |
| GO:0043409 | Biological Processes | negative regulation of MAPK cascade | -2.09126 |
| GO:0048608 | Biological Processes | reproductive structure development | -14.6131 |
| GO:0061458 | Biological Processes | reproductive system development | -14.5656 |
| GO:0007548 | Biological Processes | sex differentiation | -10.3418 |
| GO:0008406 | Biological Processes | gonad development | -9.98337 |
| GO:0045137 | Biological Processes | development of primary sexual characteristics | -9.86936 |
| GO:0008585 | Biological Processes | female gonad development | -8.58004 |
| GO:0046545 | Biological Processes | development of primary female sexual characteristics | -8.42307 |
| GO:0046660 | Biological Processes | female sex differentiation | -8.02422 |
| GO:0001541 | Biological Processes | ovarian follicle development | -6.79746 |
| GO:0042698 | Biological Processes | ovulation cycle | -6.21712 |
| GO:0008584 | Biological Processes | male gonad development | -5.99784 |
| GO:0046546 | Biological Processes | development of primary male sexual characteristics | -5.97988 |
| GO:0046661 | Biological Processes | male sex differentiation | -5.6004 |
| GO:0022602 | Biological Processes | ovulation cycle process | -5.41866 |
| GO:0044706 | Biological Processes | multi-multicellular organism process | -14.3965 |
| GO:0007565 | Biological Processes | female pregnancy | -12.041 |
| GO:0007566 | Biological Processes | embryo implantation | -8.55825 |
| GO:1901615 | Biological Processes | organic hydroxy compound metabolic process | -14.3701 |
| GO:0042445 | Biological Processes | hormone metabolic process | -8.67012 |
| GO:0006066 | Biological Processes | alcohol metabolic process | -8.06691 |
| GO:0034754 | Biological Processes | cellular hormone metabolic process | -7.46993 |
| GO:0120254 | Biological Processes | olefinic compound metabolic process | -6.38843 |
| GO:0008203 | Biological Processes | cholesterol metabolic process | -6.05253 |
| GO:1902652 | Biological Processes | secondary alcohol metabolic process | -5.87489 |
| GO:0008207 | Biological Processes | C21-steroid hormone metabolic process | -5.81479 |
| GO:0016125 | Biological Processes | sterol metabolic process | -5.7908 |
| GO:0035865 | Biological Processes | cellular response to potassium ion | -5.69856 |
| GO:0034308 | Biological Processes | primary alcohol metabolic process | -5.45861 |
| GO:0006704 | Biological Processes | glucocorticoid biosynthetic process | -5.26095 |
| GO:0035864 | Biological Processes | response to potassium ion | -5.1715 |
| GO:0006081 | Biological Processes | cellular aldehyde metabolic process | -4.91519 |
| GO:1902644 | Biological Processes | tertiary alcohol metabolic process | -4.79948 |
| GO:0042446 | Biological Processes | hormone biosynthetic process | -4.7501 |
| GO:0008211 | Biological Processes | glucocorticoid metabolic process | -4.73654 |
| GO:0120255 | Biological Processes | olefinic compound biosynthetic process | -4.61931 |
| GO:0042181 | Biological Processes | ketone biosynthetic process | -3.84799 |
| GO:0120178 | Biological Processes | steroid hormone biosynthetic process | -3.84799 |
| GO:0046165 | Biological Processes | alcohol biosynthetic process | -2.35868 |
| GO:0002521 | Biological Processes | leukocyte differentiation | -14.2511 |
| GO:0070661 | Biological Processes | leukocyte proliferation | -12.3079 |
| GO:0032943 | Biological Processes | mononuclear cell proliferation | -11.4161 |
| GO:0070663 | Biological Processes | regulation of leukocyte proliferation | -10.8957 |
| GO:1903131 | Biological Processes | mononuclear cell differentiation | -10.7468 |
| GO:0046649 | Biological Processes | lymphocyte activation | -10.6897 |
| GO:0032944 | Biological Processes | regulation of mononuclear cell proliferation | -9.85067 |
| GO:0050865 | Biological Processes | regulation of cell activation | -9.56562 |
| GO:0030098 | Biological Processes | lymphocyte differentiation | -8.97277 |
| GO:0042110 | Biological Processes | T cell activation | -8.90912 |
| GO:0002694 | Biological Processes | regulation of leukocyte activation | -8.87137 |
| GO:0046651 | Biological Processes | lymphocyte proliferation | -8.83619 |
| GO:0070665 | Biological Processes | positive regulation of leukocyte proliferation | -8.62428 |
| GO:0051249 | Biological Processes | regulation of lymphocyte activation | -8.60608 |
| GO:0050670 | Biological Processes | regulation of lymphocyte proliferation | -8.533 |
| GO:0042113 | Biological Processes | B cell activation | -8.24841 |
| GO:0051251 | Biological Processes | positive regulation of lymphocyte activation | -7.91631 |
| GO:0050671 | Biological Processes | positive regulation of lymphocyte proliferation | -7.46993 |
| GO:0032946 | Biological Processes | positive regulation of mononuclear cell proliferation | -7.44812 |
| GO:0002696 | Biological Processes | positive regulation of leukocyte activation | -7.41703 |
| GO:0030183 | Biological Processes | B cell differentiation | -7.38368 |
| GO:0050867 | Biological Processes | positive regulation of cell activation | -7.30921 |
| GO:0032640 | Biological Processes | tumor necrosis factor production | -6.65799 |
| GO:0032680 | Biological Processes | regulation of tumor necrosis factor production | -6.65799 |
| GO:0071706 | Biological Processes | tumor necrosis factor superfamily cytokine production | -6.56141 |
| GO:1903555 | Biological Processes | regulation of tumor necrosis factor superfamily cytokine production | -6.56141 |
| GO:0030888 | Biological Processes | regulation of B cell proliferation | -6.3817 |
| GO:0050864 | Biological Processes | regulation of B cell activation | -6.37787 |
| GO:0042098 | Biological Processes | T cell proliferation | -6.33385 |
| GO:0050863 | Biological Processes | regulation of T cell activation | -5.98313 |
| GO:0050871 | Biological Processes | positive regulation of B cell activation | -5.80738 |
| GO:0042129 | Biological Processes | regulation of T cell proliferation | -5.49604 |
| GO:0042100 | Biological Processes | B cell proliferation | -5.43689 |
| GO:0032760 | Biological Processes | positive regulation of tumor necrosis factor production | -5.37308 |
| GO:1903557 | Biological Processes | positive regulation of tumor necrosis factor superfamily cytokine production | -5.29103 |
| GO:0042102 | Biological Processes | positive regulation of T cell proliferation | -4.0236 |
| GO:0030890 | Biological Processes | positive regulation of B cell proliferation | -3.87867 |
| GO:0050870 | Biological Processes | positive regulation of T cell activation | -3.81 |
| GO:0032102 | Biological Processes | negative regulation of response to external stimulus | -13.391 |
| GO:0030336 | Biological Processes | negative regulation of cell migration | -11.9263 |
| GO:2000146 | Biological Processes | negative regulation of cell motility | -11.6919 |
| GO:0051271 | Biological Processes | negative regulation of cellular component movement | -11.5711 |
| GO:0040013 | Biological Processes | negative regulation of locomotion | -11.2251 |
| GO:0002683 | Biological Processes | negative regulation of immune system process | -8.29824 |
| GO:0043491 | Biological Processes | protein kinase B signaling | -13.1029 |
| GO:0051896 | Biological Processes | regulation of protein kinase B signaling | -10.7449 |
| GO:0050804 | Biological Processes | modulation of chemical synaptic transmission | -8.23644 |
| GO:0099177 | Biological Processes | regulation of trans-synaptic signaling | -8.22623 |
| GO:0070371 | Biological Processes | ERK1 and ERK2 cascade | -8.21141 |
| GO:0051897 | Biological Processes | positive regulation of protein kinase B signaling | -7.86891 |
| GO:0099536 | Biological Processes | synaptic signaling | -7.75441 |
| GO:0070374 | Biological Processes | positive regulation of ERK1 and ERK2 cascade | -7.30768 |
| GO:0070372 | Biological Processes | regulation of ERK1 and ERK2 cascade | -7.28595 |
| GO:0007268 | Biological Processes | chemical synaptic transmission | -7.00359 |
| GO:0098916 | Biological Processes | anterograde trans-synaptic signaling | -7.00359 |
| GO:0099537 | Biological Processes | trans-synaptic signaling | -6.95182 |
| GO:0050806 | Biological Processes | positive regulation of synaptic transmission | -6.9333 |
| GO:0051966 | Biological Processes | regulation of synaptic transmission, glutamatergic | -6.21712 |
| GO:0035249 | Biological Processes | synaptic transmission, glutamatergic | -5.59409 |
| GO:1903076 | Biological Processes | regulation of protein localization to plasma membrane | -5.33164 |
| GO:1904375 | Biological Processes | regulation of protein localization to cell periphery | -4.94149 |
| GO:1903827 | Biological Processes | regulation of cellular protein localization | -4.5001 |
| GO:1903829 | Biological Processes | positive regulation of cellular protein localization | -4.33165 |
| GO:0051968 | Biological Processes | positive regulation of synaptic transmission, glutamatergic | -4.32136 |
| GO:1905475 | Biological Processes | regulation of protein localization to membrane | -4.23983 |
| GO:1903078 | Biological Processes | positive regulation of protein localization to plasma membrane | -3.37542 |
| GO:0072659 | Biological Processes | protein localization to plasma membrane | -3.25006 |
| GO:1904377 | Biological Processes | positive regulation of protein localization to cell periphery | -3.23894 |
| GO:1990778 | Biological Processes | protein localization to cell periphery | -2.94258 |
| GO:1905477 | Biological Processes | positive regulation of protein localization to membrane | -2.69964 |
| GO:0002526 | Biological Processes | acute inflammatory response | -12.9391 |
| GO:0006953 | Biological Processes | acute-phase response | -10.6527 |
| GO:0010575 | Biological Processes | positive regulation of vascular endothelial growth factor production | -10.3233 |
| GO:0010573 | Biological Processes | vascular endothelial growth factor production | -9.90531 |
| GO:0150076 | Biological Processes | neuroinflammatory response | -9.06329 |
| GO:0060627 | Biological Processes | regulation of vesicle-mediated transport | -8.46653 |
| GO:0010574 | Biological Processes | regulation of vascular endothelial growth factor production | -8.31596 |
| GO:0051341 | Biological Processes | regulation of oxidoreductase activity | -8.21673 |
| GO:0001660 | Biological Processes | fever generation | -8.04731 |
| GO:0051222 | Biological Processes | positive regulation of protein transport | -7.44252 |
| GO:0150077 | Biological Processes | regulation of neuroinflammatory response | -7.42687 |
| GO:0006909 | Biological Processes | phagocytosis | -7.35733 |
| GO:1904951 | Biological Processes | positive regulation of establishment of protein localization | -7.25081 |
| GO:0050999 | Biological Processes | regulation of nitric-oxide synthase activity | -7.2124 |
| GO:0034330 | Biological Processes | cell junction organization | -7.16968 |
| GO:0031649 | Biological Processes | heat generation | -7.08546 |
| GO:0061028 | Biological Processes | establishment of endothelial barrier | -6.88283 |
| GO:0032768 | Biological Processes | regulation of monooxygenase activity | -6.56047 |
| GO:0051353 | Biological Processes | positive regulation of oxidoreductase activity | -6.56047 |
| GO:0031663 | Biological Processes | lipopolysaccharide-mediated signaling pathway | -6.52347 |
| GO:0031622 | Biological Processes | positive regulation of fever generation | -6.36906 |
| GO:0045216 | Biological Processes | cell-cell junction organization | -6.33385 |
| GO:0002675 | Biological Processes | positive regulation of acute inflammatory response | -6.2676 |
| GO:0001885 | Biological Processes | endothelial cell development | -6.21712 |
| GO:0033077 | Biological Processes | T cell differentiation in thymus | -6.03551 |
| GO:1901889 | Biological Processes | negative regulation of cell junction assembly | -6.02607 |
| GO:0031620 | Biological Processes | regulation of fever generation | -5.99031 |
| GO:1901099 | Biological Processes | negative regulation of signal transduction in absence of ligand | -5.97072 |
| GO:2001240 | Biological Processes | negative regulation of extrinsic apoptotic signaling pathway in absence of ligand | -5.97072 |
| GO:0046889 | Biological Processes | positive regulation of lipid biosynthetic process | -5.84209 |
| GO:0031652 | Biological Processes | positive regulation of heat generation | -5.69856 |
| GO:0030217 | Biological Processes | T cell differentiation | -5.63245 |
| GO:0050764 | Biological Processes | regulation of phagocytosis | -5.50278 |
| GO:2001239 | Biological Processes | regulation of extrinsic apoptotic signaling pathway in absence of ligand | -5.49895 |
| GO:0031392 | Biological Processes | regulation of prostaglandin biosynthetic process | -5.46114 |
| GO:0010721 | Biological Processes | negative regulation of cell development | -5.3822 |
| GO:0031650 | Biological Processes | regulation of heat generation | -5.35713 |
| GO:0002673 | Biological Processes | regulation of acute inflammatory response | -5.30502 |
| GO:0042304 | Biological Processes | regulation of fatty acid biosynthetic process | -5.2688 |
| GO:2001279 | Biological Processes | regulation of unsaturated fatty acid biosynthetic process | -5.26095 |
| GO:0034329 | Biological Processes | cell junction assembly | -5.20155 |
| GO:0150078 | Biological Processes | positive regulation of neuroinflammatory response | -5.00946 |
| GO:1901550 | Biological Processes | regulation of endothelial cell development | -5.00946 |
| GO:1903140 | Biological Processes | regulation of establishment of endothelial barrier | -5.00946 |
| GO:0045446 | Biological Processes | endothelial cell differentiation | -4.97553 |
| GO:0038034 | Biological Processes | signal transduction in absence of ligand | -4.77649 |
| GO:0097192 | Biological Processes | extrinsic apoptotic signaling pathway in absence of ligand | -4.77649 |
| GO:0050766 | Biological Processes | positive regulation of phagocytosis | -4.7501 |
| GO:0050768 | Biological Processes | negative regulation of neurogenesis | -4.70333 |
| GO:0003158 | Biological Processes | endothelium development | -4.68843 |
| GO:0045723 | Biological Processes | positive regulation of fatty acid biosynthetic process | -4.67657 |
| GO:0051961 | Biological Processes | negative regulation of nervous system development | -4.62995 |
| GO:0032770 | Biological Processes | positive regulation of monooxygenase activity | -4.15581 |
| GO:1901888 | Biological Processes | regulation of cell junction assembly | -3.92594 |
| GO:0045601 | Biological Processes | regulation of endothelial cell differentiation | -3.70503 |
| GO:0007043 | Biological Processes | cell-cell junction assembly | -3.39841 |
| GO:0045732 | Biological Processes | positive regulation of protein catabolic process | -2.66367 |
| GO:0097191 | Biological Processes | extrinsic apoptotic signaling pathway | -12.91 |
| GO:2001237 | Biological Processes | negative regulation of extrinsic apoptotic signaling pathway | -11.8327 |
| GO:2001236 | Biological Processes | regulation of extrinsic apoptotic signaling pathway | -11.6248 |
| GO:0008625 | Biological Processes | extrinsic apoptotic signaling pathway via death domain receptors | -7.39793 |
| GO:1902041 | Biological Processes | regulation of extrinsic apoptotic signaling pathway via death domain receptors | -5.2688 |
| GO:1902042 | Biological Processes | negative regulation of extrinsic apoptotic signaling pathway via death domain receptors | -4.27788 |
| GO:0035094 | Biological Processes | response to nicotine | -12.8457 |
| GO:0001101 | Biological Processes | response to acid chemical | -7.51406 |
| GO:0043200 | Biological Processes | response to amino acid | -6.49683 |
| GO:0006970 | Biological Processes | response to osmotic stress | -5.76448 |
| GO:0034250 | Biological Processes | positive regulation of cellular amide metabolic process | -5.64658 |
| GO:0034248 | Biological Processes | regulation of cellular amide metabolic process | -5.38978 |
| GO:0046677 | Biological Processes | response to antibiotic | -5.34205 |
| GO:0034349 | Biological Processes | glial cell apoptotic process | -5.08791 |
| GO:0043525 | Biological Processes | positive regulation of neuron apoptotic process | -4.97412 |
| GO:1902004 | Biological Processes | positive regulation of amyloid-beta formation | -4.8657 |
| GO:0050435 | Biological Processes | amyloid-beta metabolic process | -4.72413 |
| GO:1902993 | Biological Processes | positive regulation of amyloid precursor protein catabolic process | -4.56452 |
| GO:1901216 | Biological Processes | positive regulation of neuron death | -4.09184 |
| GO:1902003 | Biological Processes | regulation of amyloid-beta formation | -3.70503 |
| GO:1902991 | Biological Processes | regulation of amyloid precursor protein catabolic process | -3.52918 |
| GO:0034205 | Biological Processes | amyloid-beta formation | -3.48324 |
| GO:0042987 | Biological Processes | amyloid precursor protein catabolic process | -3.25752 |
| GO:0097194 | Biological Processes | execution phase of apoptosis | -2.97519 |
| GO:0042982 | Biological Processes | amyloid precursor protein metabolic process | -2.83588 |
| GO:0033209 | Biological Processes | tumor necrosis factor-mediated signaling pathway | -2.78447 |
| GO:0006809 | Biological Processes | nitric oxide biosynthetic process | -12.8174 |
| GO:0046209 | Biological Processes | nitric oxide metabolic process | -12.5586 |
| GO:2001057 | Biological Processes | reactive nitrogen species metabolic process | -12.5089 |
| GO:0045428 | Biological Processes | regulation of nitric oxide biosynthetic process | -9.90531 |
| GO:0080164 | Biological Processes | regulation of nitric oxide metabolic process | -9.80545 |
| GO:0045429 | Biological Processes | positive regulation of nitric oxide biosynthetic process | -9.32471 |
| GO:1904407 | Biological Processes | positive regulation of nitric oxide metabolic process | -9.25679 |
| GO:0010638 | Biological Processes | positive regulation of organelle organization | -7.98145 |
| GO:0035690 | Biological Processes | cellular response to drug | -7.93118 |
| GO:0045787 | Biological Processes | positive regulation of cell cycle | -7.27419 |
| GO:0032309 | Biological Processes | icosanoid secretion | -7.16203 |
| GO:0071715 | Biological Processes | icosanoid transport | -6.79746 |
| GO:0090068 | Biological Processes | positive regulation of cell cycle process | -5.86687 |
| GO:0015908 | Biological Processes | fatty acid transport | -5.73925 |
| GO:0045840 | Biological Processes | positive regulation of mitotic nuclear division | -5.49895 |
| GO:0140353 | Biological Processes | lipid export from cell | -5.49895 |
| GO:0032310 | Biological Processes | prostaglandin secretion | -5.26095 |
| GO:0015718 | Biological Processes | monocarboxylic acid transport | -5.19297 |
| GO:0015732 | Biological Processes | prostaglandin transport | -5.00946 |
| GO:0051785 | Biological Processes | positive regulation of nuclear division | -5.00439 |
| GO:0046942 | Biological Processes | carboxylic acid transport | -4.34919 |
| GO:0043270 | Biological Processes | positive regulation of ion transport | -4.3404 |
| GO:0015849 | Biological Processes | organic acid transport | -4.09199 |
| GO:0032892 | Biological Processes | positive regulation of organic acid transport | -3.9424 |
| GO:0007088 | Biological Processes | regulation of mitotic nuclear division | -3.88002 |
| GO:1903793 | Biological Processes | positive regulation of anion transport | -3.84799 |
| GO:0015711 | Biological Processes | organic anion transport | -3.73295 |
| GO:0051783 | Biological Processes | regulation of nuclear division | -3.49058 |
| GO:0032890 | Biological Processes | regulation of organic acid transport | -3.27639 |
| GO:0044070 | Biological Processes | regulation of anion transport | -2.94598 |
| GO:0006820 | Biological Processes | anion transport | -2.91012 |
| GO:0043500 | Biological Processes | muscle adaptation | -2.53649 |
| GO:0140014 | Biological Processes | mitotic nuclear division | -2.31236 |
| GO:0048285 | Biological Processes | organelle fission | -2.22568 |
| GO:0007584 | Biological Processes | response to nutrient | -12.5906 |
| GO:0009612 | Biological Processes | response to mechanical stimulus | -7.3845 |
| GO:0033273 | Biological Processes | response to vitamin | -7.1255 |
| GO:0045907 | Biological Processes | positive regulation of vasoconstriction | -4.11761 |
| GO:0071260 | Biological Processes | cellular response to mechanical stimulus | -3.03587 |
| GO:0010469 | Biological Processes | regulation of signaling receptor activity | -12.5628 |
| GO:0014823 | Biological Processes | response to activity | -9.26857 |
| GO:2000273 | Biological Processes | positive regulation of signaling receptor activity | -7.16203 |
| GO:0031644 | Biological Processes | regulation of nervous system process | -3.43233 |
| GO:0008202 | Biological Processes | steroid metabolic process | -12.3251 |
| GO:0006694 | Biological Processes | steroid biosynthetic process | -9.53503 |
| GO:0046890 | Biological Processes | regulation of lipid biosynthetic process | -8.1755 |
| GO:1901617 | Biological Processes | organic hydroxy compound biosynthetic process | -7.07379 |
| GO:0019218 | Biological Processes | regulation of steroid metabolic process | -6.88018 |
| GO:0050810 | Biological Processes | regulation of steroid biosynthetic process | -6.18572 |
| GO:0045833 | Biological Processes | negative regulation of lipid metabolic process | -5.4154 |
| GO:0062014 | Biological Processes | negative regulation of small molecule metabolic process | -5.37308 |
| GO:2001235 | Biological Processes | positive regulation of apoptotic signaling pathway | -4.92469 |
| GO:2001244 | Biological Processes | positive regulation of intrinsic apoptotic signaling pathway | -3.4609 |
| GO:0051055 | Biological Processes | negative regulation of lipid biosynthetic process | -3.43896 |
| GO:0009636 | Biological Processes | response to toxic substance | -11.9868 |
| GO:0042743 | Biological Processes | hydrogen peroxide metabolic process | -10.1158 |
| GO:0019430 | Biological Processes | removal of superoxide radicals | -8.70479 |
| GO:0071450 | Biological Processes | cellular response to oxygen radical | -8.50799 |
| GO:0071451 | Biological Processes | cellular response to superoxide | -8.50799 |
| GO:0098869 | Biological Processes | cellular oxidant detoxification | -8.39267 |
| GO:0000303 | Biological Processes | response to superoxide | -8.24322 |
| GO:0000305 | Biological Processes | response to oxygen radical | -8.16182 |
| GO:1990748 | Biological Processes | cellular detoxification | -7.97151 |
| GO:0097237 | Biological Processes | cellular response to toxic substance | -7.76987 |
| GO:0098754 | Biological Processes | detoxification | -7.15925 |
| GO:0050665 | Biological Processes | hydrogen peroxide biosynthetic process | -7.08546 |
| GO:1903409 | Biological Processes | reactive oxygen species biosynthetic process | -6.3817 |
| GO:0042311 | Biological Processes | vasodilation | -5.34205 |
| GO:0032355 | Biological Processes | response to estradiol | -11.9241 |
| GO:0009895 | Biological Processes | negative regulation of catabolic process | -7.20451 |
| GO:0071392 | Biological Processes | cellular response to estradiol stimulus | -5.71833 |
| GO:0042176 | Biological Processes | regulation of protein catabolic process | -5.43669 |
| GO:0031099 | Biological Processes | regeneration | -5.14475 |
| GO:0001889 | Biological Processes | liver development | -4.60135 |
| GO:0061008 | Biological Processes | hepaticobiliary system development | -4.55923 |
| GO:0031100 | Biological Processes | animal organ regeneration | -4.50729 |
| GO:0097421 | Biological Processes | liver regeneration | -4.11761 |
| GO:0042177 | Biological Processes | negative regulation of protein catabolic process | -3.72063 |
| GO:0042542 | Biological Processes | response to hydrogen peroxide | -11.7718 |
| GO:1901214 | Biological Processes | regulation of neuron death | -10.9849 |
| GO:0070997 | Biological Processes | neuron death | -10.3622 |
| GO:0043523 | Biological Processes | regulation of neuron apoptotic process | -10.1607 |
| GO:0051402 | Biological Processes | neuron apoptotic process | -9.52843 |
| GO:0008631 | Biological Processes | intrinsic apoptotic signaling pathway in response to oxidative stress | -9.00193 |
| GO:0036473 | Biological Processes | cell death in response to oxidative stress | -8.54796 |
| GO:1902175 | Biological Processes | regulation of oxidative stress-induced intrinsic apoptotic signaling pathway | -8.16182 |
| GO:1903201 | Biological Processes | regulation of oxidative stress-induced cell death | -7.66809 |
| GO:1901215 | Biological Processes | negative regulation of neuron death | -7.51147 |
| GO:1900407 | Biological Processes | regulation of cellular response to oxidative stress | -7.18349 |
| GO:1902882 | Biological Processes | regulation of response to oxidative stress | -6.90626 |
| GO:1902176 | Biological Processes | negative regulation of oxidative stress-induced intrinsic apoptotic signaling pathway | -6.88741 |
| GO:1903202 | Biological Processes | negative regulation of oxidative stress-induced cell death | -6.79746 |
| GO:0043524 | Biological Processes | negative regulation of neuron apoptotic process | -5.92681 |
| GO:0070301 | Biological Processes | cellular response to hydrogen peroxide | -5.45861 |
| GO:0007346 | Biological Processes | regulation of mitotic cell cycle | -11.5616 |
| GO:0045786 | Biological Processes | negative regulation of cell cycle | -7.6637 |
| GO:0010564 | Biological Processes | regulation of cell cycle process | -7.47171 |
| GO:0045930 | Biological Processes | negative regulation of mitotic cell cycle | -7.10209 |
| GO:0000082 | Biological Processes | G1/S transition of mitotic cell cycle | -6.15087 |
| GO:1903047 | Biological Processes | mitotic cell cycle process | -6.03736 |
| GO:2000045 | Biological Processes | regulation of G1/S transition of mitotic cell cycle | -5.97988 |
| GO:0044843 | Biological Processes | cell cycle G1/S phase transition | -5.80631 |
| GO:1902806 | Biological Processes | regulation of cell cycle G1/S phase transition | -5.5551 |
| GO:0002244 | Biological Processes | hematopoietic progenitor cell differentiation | -5.13635 |
| GO:2000134 | Biological Processes | negative regulation of G1/S transition of mitotic cell cycle | -4.33617 |
| GO:0060218 | Biological Processes | hematopoietic stem cell differentiation | -4.32136 |
| GO:0044772 | Biological Processes | mitotic cell cycle phase transition | -4.21818 |
| GO:1902807 | Biological Processes | negative regulation of cell cycle G1/S phase transition | -4.16313 |
| GO:1901990 | Biological Processes | regulation of mitotic cell cycle phase transition | -4.13945 |
| GO:0042149 | Biological Processes | cellular response to glucose starvation | -3.70503 |
| GO:0044770 | Biological Processes | cell cycle phase transition | -3.6804 |
| GO:0010665 | Biological Processes | regulation of cardiac muscle cell apoptotic process | -3.60149 |
| GO:0010662 | Biological Processes | regulation of striated muscle cell apoptotic process | -3.55281 |
| GO:1901987 | Biological Processes | regulation of cell cycle phase transition | -3.51396 |
| GO:0010659 | Biological Processes | cardiac muscle cell apoptotic process | -3.50599 |
| GO:0010658 | Biological Processes | striated muscle cell apoptotic process | -3.4609 |
| GO:1901991 | Biological Processes | negative regulation of mitotic cell cycle phase transition | -3.07738 |
| GO:1901988 | Biological Processes | negative regulation of cell cycle phase transition | -2.55292 |
| GO:0010948 | Biological Processes | negative regulation of cell cycle process | -2.29671 |
| GO:0045123 | Biological Processes | cellular extravasation | -11.2552 |
| GO:0050901 | Biological Processes | leukocyte tethering or rolling | -7.86424 |
| GO:0098742 | Biological Processes | cell-cell adhesion via plasma-membrane adhesion molecules | -5.42745 |
| GO:0002691 | Biological Processes | regulation of cellular extravasation | -4.04456 |
| GO:0007157 | Biological Processes | heterophilic cell-cell adhesion via plasma membrane cell adhesion molecules | -3.76025 |
| GO:0030029 | Biological Processes | actin filament-based process | -2.58942 |
| GO:0030036 | Biological Processes | actin cytoskeleton organization | -2.2005 |
| GO:0097435 | Biological Processes | supramolecular fiber organization | -2.09656 |
| GO:0048589 | Biological Processes | developmental growth | -10.884 |
| GO:0048638 | Biological Processes | regulation of developmental growth | -9.53273 |
| GO:0045927 | Biological Processes | positive regulation of growth | -8.01747 |
| GO:0048639 | Biological Processes | positive regulation of developmental growth | -6.75806 |
| GO:0050927 | Biological Processes | positive regulation of positive chemotaxis | -6.47444 |
| GO:0050926 | Biological Processes | regulation of positive chemotaxis | -6.40266 |
| GO:0050918 | Biological Processes | positive chemotaxis | -6.31429 |
| GO:0030307 | Biological Processes | positive regulation of cell growth | -5.5852 |
| GO:0050930 | Biological Processes | induction of positive chemotaxis | -5.35713 |
| GO:0060322 | Biological Processes | head development | -4.80873 |
| GO:0000904 | Biological Processes | cell morphogenesis involved in differentiation | -4.47306 |
| GO:0061564 | Biological Processes | axon development | -3.95679 |
| GO:0048667 | Biological Processes | cell morphogenesis involved in neuron differentiation | -3.55138 |
| GO:0032989 | Biological Processes | cellular component morphogenesis | -3.4725 |
| GO:0007420 | Biological Processes | brain development | -3.43383 |
| GO:0007409 | Biological Processes | axonogenesis | -3.3543 |
| GO:0001764 | Biological Processes | neuron migration | -3.30099 |
| GO:0048812 | Biological Processes | neuron projection morphogenesis | -3.23722 |
| GO:0120039 | Biological Processes | plasma membrane bounded cell projection morphogenesis | -3.17543 |
| GO:0048858 | Biological Processes | cell projection morphogenesis | -3.15925 |
| GO:0032990 | Biological Processes | cell part morphogenesis | -3.08794 |
| GO:0008361 | Biological Processes | regulation of cell size | -3.05054 |
| GO:0032535 | Biological Processes | regulation of cellular component size | -2.69243 |
| GO:0048588 | Biological Processes | developmental cell growth | -2.65017 |
| GO:0060560 | Biological Processes | developmental growth involved in morphogenesis | -2.64347 |
| GO:0048675 | Biological Processes | axon extension | -2.54667 |
| GO:0050770 | Biological Processes | regulation of axonogenesis | -2.24373 |
| GO:1990138 | Biological Processes | neuron projection extension | -2.11176 |
| GO:0030162 | Biological Processes | regulation of proteolysis | -10.8508 |
| GO:0045861 | Biological Processes | negative regulation of proteolysis | -6.88643 |
| GO:0061042 | Biological Processes | vascular wound healing | -4.73654 |
| GO:0030856 | Biological Processes | regulation of epithelial cell differentiation | -4.49098 |
| GO:0043154 | Biological Processes | negative regulation of cysteine-type endopeptidase activity involved in apoptotic process | -4.46278 |
| GO:2000117 | Biological Processes | negative regulation of cysteine-type endopeptidase activity | -4.29607 |
| GO:0060055 | Biological Processes | angiogenesis involved in wound healing | -4.27788 |
| GO:0051346 | Biological Processes | negative regulation of hydrolase activity | -3.5803 |
| GO:0010951 | Biological Processes | negative regulation of endopeptidase activity | -3.50045 |
| GO:0010466 | Biological Processes | negative regulation of peptidase activity | -3.42308 |
| GO:0042531 | Biological Processes | positive regulation of tyrosine phosphorylation of STAT protein | -3.23894 |
| GO:0071229 | Biological Processes | cellular response to acid chemical | -3.05154 |
| GO:0042509 | Biological Processes | regulation of tyrosine phosphorylation of STAT protein | -2.9605 |
| GO:0007260 | Biological Processes | tyrosine phosphorylation of STAT protein | -2.91747 |
| GO:1905952 | Biological Processes | regulation of lipid localization | -10.8391 |
| GO:0010876 | Biological Processes | lipid localization | -10.4754 |
| GO:0071346 | Biological Processes | cellular response to interferon-gamma | -7.91975 |
| GO:0010742 | Biological Processes | macrophage derived foam cell differentiation | -7.54288 |
| GO:0090077 | Biological Processes | foam cell differentiation | -7.54288 |
| GO:0006869 | Biological Processes | lipid transport | -7.52807 |
| GO:0034341 | Biological Processes | response to interferon-gamma | -7.38368 |
| GO:0032370 | Biological Processes | positive regulation of lipid transport | -7.33474 |
| GO:0032368 | Biological Processes | regulation of lipid transport | -7.2187 |
| GO:1905954 | Biological Processes | positive regulation of lipid localization | -6.63362 |
| GO:0010875 | Biological Processes | positive regulation of cholesterol efflux | -6.40266 |
| GO:0010743 | Biological Processes | regulation of macrophage derived foam cell differentiation | -6.02607 |
| GO:0032373 | Biological Processes | positive regulation of sterol transport | -5.71833 |
| GO:0032376 | Biological Processes | positive regulation of cholesterol transport | -5.71833 |
| GO:0010745 | Biological Processes | negative regulation of macrophage derived foam cell differentiation | -5.46114 |
| GO:0015850 | Biological Processes | organic hydroxy compound transport | -5.3457 |
| GO:0010874 | Biological Processes | regulation of cholesterol efflux | -5.19865 |
| GO:0033344 | Biological Processes | cholesterol efflux | -4.6734 |
| GO:0001959 | Biological Processes | regulation of cytokine-mediated signaling pathway | -4.55923 |
| GO:0031348 | Biological Processes | negative regulation of defense response | -4.4948 |
| GO:0032371 | Biological Processes | regulation of sterol transport | -4.46278 |
| GO:0032374 | Biological Processes | regulation of cholesterol transport | -4.46278 |
| GO:0060333 | Biological Processes | interferon-gamma-mediated signaling pathway | -4.46158 |
| GO:0060759 | Biological Processes | regulation of response to cytokine stimulus | -4.39931 |
| GO:0050728 | Biological Processes | negative regulation of inflammatory response | -4.22809 |
| GO:0055090 | Biological Processes | acylglycerol homeostasis | -3.87867 |
| GO:0070328 | Biological Processes | triglyceride homeostasis | -3.87867 |
| GO:0030301 | Biological Processes | cholesterol transport | -3.77673 |
| GO:0042752 | Biological Processes | regulation of circadian rhythm | -3.72063 |
| GO:0043124 | Biological Processes | negative regulation of I-kappaB kinase/NF-kappaB signaling | -3.62657 |
| GO:0015918 | Biological Processes | sterol transport | -3.60133 |
| GO:0046486 | Biological Processes | glycerolipid metabolic process | -3.50213 |
| GO:0045824 | Biological Processes | negative regulation of innate immune response | -3.20262 |
| GO:0055088 | Biological Processes | lipid homeostasis | -3.18987 |
| GO:0001960 | Biological Processes | negative regulation of cytokine-mediated signaling pathway | -3.03587 |
| GO:1905897 | Biological Processes | regulation of response to endoplasmic reticulum stress | -3.02041 |
| GO:0060761 | Biological Processes | negative regulation of response to cytokine stimulus | -2.9605 |
| GO:0050777 | Biological Processes | negative regulation of immune response | -2.94783 |
| GO:0006641 | Biological Processes | triglyceride metabolic process | -2.77197 |
| GO:0002832 | Biological Processes | negative regulation of response to biotic stimulus | -2.6765 |
| GO:0032411 | Biological Processes | positive regulation of transporter activity | -2.54667 |
| GO:0006639 | Biological Processes | acylglycerol metabolic process | -2.46767 |
| GO:0006638 | Biological Processes | neutral lipid metabolic process | -2.45817 |
| GO:0006650 | Biological Processes | glycerophospholipid metabolic process | -2.23592 |
| GO:0032868 | Biological Processes | response to insulin | -10.5304 |
| GO:0032869 | Biological Processes | cellular response to insulin stimulus | -7.56043 |
| GO:0008286 | Biological Processes | insulin receptor signaling pathway | -6.45289 |
| GO:1900076 | Biological Processes | regulation of cellular response to insulin stimulus | -6.34772 |
| GO:0046626 | Biological Processes | regulation of insulin receptor signaling pathway | -6.28139 |
| GO:0046888 | Biological Processes | negative regulation of hormone secretion | -4.80331 |
| GO:1903531 | Biological Processes | negative regulation of secretion by cell | -4.60135 |
| GO:0050709 | Biological Processes | negative regulation of protein secretion | -4.60013 |
| GO:0051048 | Biological Processes | negative regulation of secretion | -4.2875 |
| GO:0046627 | Biological Processes | negative regulation of insulin receptor signaling pathway | -4.08055 |
| GO:1900077 | Biological Processes | negative regulation of cellular response to insulin stimulus | -4.00957 |
| GO:0051224 | Biological Processes | negative regulation of protein transport | -3.64009 |
| GO:1904950 | Biological Processes | negative regulation of establishment of protein localization | -3.58863 |
| GO:0050994 | Biological Processes | regulation of lipid catabolic process | -3.39624 |
| GO:0016042 | Biological Processes | lipid catabolic process | -2.16369 |
| GO:0014065 | Biological Processes | phosphatidylinositol 3-kinase signaling | -10.325 |
| GO:0048015 | Biological Processes | phosphatidylinositol-mediated signaling | -9.49089 |
| GO:0048017 | Biological Processes | inositol lipid-mediated signaling | -9.40419 |
| GO:0014066 | Biological Processes | regulation of phosphatidylinositol 3-kinase signaling | -8.1604 |
| GO:0006109 | Biological Processes | regulation of carbohydrate metabolic process | -8.03881 |
| GO:0010827 | Biological Processes | regulation of glucose transmembrane transport | -7.56331 |
| GO:1904659 | Biological Processes | glucose transmembrane transport | -6.61028 |
| GO:0008645 | Biological Processes | hexose transmembrane transport | -6.54158 |
| GO:0015749 | Biological Processes | monosaccharide transmembrane transport | -6.49683 |
| GO:0034219 | Biological Processes | carbohydrate transmembrane transport | -6.45289 |
| GO:0031331 | Biological Processes | positive regulation of cellular catabolic process | -6.16436 |
| GO:0005975 | Biological Processes | carbohydrate metabolic process | -5.9345 |
| GO:0014068 | Biological Processes | positive regulation of phosphatidylinositol 3-kinase signaling | -5.92276 |
| GO:0010675 | Biological Processes | regulation of cellular carbohydrate metabolic process | -5.90938 |
| GO:0008643 | Biological Processes | carbohydrate transport | -5.82408 |
| GO:0009896 | Biological Processes | positive regulation of catabolic process | -5.65863 |
| GO:0044262 | Biological Processes | cellular carbohydrate metabolic process | -5.33565 |
| GO:0006006 | Biological Processes | glucose metabolic process | -5.16997 |
| GO:0010906 | Biological Processes | regulation of glucose metabolic process | -5.04541 |
| GO:0046324 | Biological Processes | regulation of glucose import | -4.94439 |
| GO:0046628 | Biological Processes | positive regulation of insulin receptor signaling pathway | -4.73654 |
| GO:0019318 | Biological Processes | hexose metabolic process | -4.70166 |
| GO:1900078 | Biological Processes | positive regulation of cellular response to insulin stimulus | -4.56452 |
| GO:0046323 | Biological Processes | glucose import | -4.55304 |
| GO:0045913 | Biological Processes | positive regulation of carbohydrate metabolic process | -4.53 |
| GO:0005996 | Biological Processes | monosaccharide metabolic process | -4.50422 |
| GO:0043255 | Biological Processes | regulation of carbohydrate biosynthetic process | -4.09184 |
| GO:0032885 | Biological Processes | regulation of polysaccharide biosynthetic process | -4.04456 |
| GO:0046326 | Biological Processes | positive regulation of glucose import | -3.97554 |
| GO:0010907 | Biological Processes | positive regulation of glucose metabolic process | -3.91013 |
| GO:0032881 | Biological Processes | regulation of polysaccharide metabolic process | -3.81805 |
| GO:0034764 | Biological Processes | positive regulation of transmembrane transport | -3.78211 |
| GO:0010828 | Biological Processes | positive regulation of glucose transmembrane transport | -3.76025 |
| GO:0010676 | Biological Processes | positive regulation of cellular carbohydrate metabolic process | -3.52918 |
| GO:0000271 | Biological Processes | polysaccharide biosynthetic process | -3.16736 |
| GO:0016051 | Biological Processes | carbohydrate biosynthetic process | -2.87528 |
| GO:0005976 | Biological Processes | polysaccharide metabolic process | -2.6765 |
| GO:0007610 | Biological Processes | behavior | -10.2028 |
| GO:0050890 | Biological Processes | cognition | -8.75059 |
| GO:0007611 | Biological Processes | learning or memory | -8.0761 |
| GO:0050805 | Biological Processes | negative regulation of synaptic transmission | -4.6734 |
| GO:0007612 | Biological Processes | learning | -4.64441 |
| GO:0051954 | Biological Processes | positive regulation of amine transport | -4.04456 |
| GO:0008542 | Biological Processes | visual learning | -3.81805 |
| GO:0007613 | Biological Processes | memory | -3.76252 |
| GO:0048512 | Biological Processes | circadian behavior | -3.73233 |
| GO:0007622 | Biological Processes | rhythmic behavior | -3.67832 |
| GO:0007632 | Biological Processes | visual behavior | -3.65217 |
| GO:0051898 | Biological Processes | negative regulation of protein kinase B signaling | -3.52918 |
| GO:0021537 | Biological Processes | telencephalon development | -3.50837 |
| GO:0008306 | Biological Processes | associative learning | -3.0835 |
| GO:0007626 | Biological Processes | locomotory behavior | -3.06838 |
| GO:0048167 | Biological Processes | regulation of synaptic plasticity | -2.92324 |
| GO:0051952 | Biological Processes | regulation of amine transport | -2.88964 |
| GO:0000079 | Biological Processes | regulation of cyclin-dependent protein serine/threonine kinase activity | -2.80989 |
| GO:0015837 | Biological Processes | amine transport | -2.78447 |
| GO:0060291 | Biological Processes | long-term synaptic potentiation | -2.78447 |
| GO:1904029 | Biological Processes | regulation of cyclin-dependent protein kinase activity | -2.75959 |
| GO:0030900 | Biological Processes | forebrain development | -2.69737 |
| GO:0021761 | Biological Processes | limbic system development | -2.68801 |
| GO:0043266 | Biological Processes | regulation of potassium ion transport | -2.6651 |
| GO:0030534 | Biological Processes | adult behavior | -2.44875 |
| GO:0021543 | Biological Processes | pallium development | -2.12565 |
| GO:0060485 | Biological Processes | mesenchyme development | -10.097 |
| GO:0048762 | Biological Processes | mesenchymal cell differentiation | -9.66844 |
| GO:0048771 | Biological Processes | tissue remodeling | -9.46902 |
| GO:0051781 | Biological Processes | positive regulation of cell division | -8.78039 |
| GO:1903532 | Biological Processes | positive regulation of secretion by cell | -7.69818 |
| GO:0051047 | Biological Processes | positive regulation of secretion | -7.34533 |
| GO:0001837 | Biological Processes | epithelial to mesenchymal transition | -7.02524 |
| GO:0010717 | Biological Processes | regulation of epithelial to mesenchymal transition | -6.90626 |
| GO:0051302 | Biological Processes | regulation of cell division | -6.70757 |
| GO:0002248 | Biological Processes | connective tissue replacement involved in inflammatory response wound healing | -6.61137 |
| GO:0002246 | Biological Processes | wound healing involved in inflammatory response | -6.16567 |
| GO:0090594 | Biological Processes | inflammatory response to wounding | -5.26095 |
| GO:0010718 | Biological Processes | positive regulation of epithelial to mesenchymal transition | -5.16465 |
| GO:0010934 | Biological Processes | macrophage cytokine production | -5.08791 |
| GO:0010935 | Biological Processes | regulation of macrophage cytokine production | -5.08791 |
| GO:0097709 | Biological Processes | connective tissue replacement | -4.93555 |
| GO:0050714 | Biological Processes | positive regulation of protein secretion | -4.76408 |
| GO:0016331 | Biological Processes | morphogenesis of embryonic epithelium | -4.58721 |
| GO:0035148 | Biological Processes | tube formation | -4.57317 |
| GO:0061082 | Biological Processes | myeloid leukocyte cytokine production | -4.36639 |
| GO:0070168 | Biological Processes | negative regulation of biomineral tissue development | -4.32136 |
| GO:0110150 | Biological Processes | negative regulation of biomineralization | -4.27788 |
| GO:0014020 | Biological Processes | primary neural tube formation | -4.12709 |
| GO:1905207 | Biological Processes | regulation of cardiocyte differentiation | -4.11761 |
| GO:0001841 | Biological Processes | neural tube formation | -3.99055 |
| GO:0003203 | Biological Processes | endocardial cushion morphogenesis | -3.97554 |
| GO:0071634 | Biological Processes | regulation of transforming growth factor beta production | -3.91013 |
| GO:1905314 | Biological Processes | semi-lunar valve development | -3.91013 |
| GO:0071604 | Biological Processes | transforming growth factor beta production | -3.84799 |
| GO:0001974 | Biological Processes | blood vessel remodeling | -3.76025 |
| GO:0003197 | Biological Processes | endocardial cushion development | -3.73233 |
| GO:0003206 | Biological Processes | cardiac chamber morphogenesis | -3.72063 |
| GO:0001838 | Biological Processes | embryonic epithelial tube formation | -3.70691 |
| GO:0010862 | Biological Processes | positive regulation of pathway-restricted SMAD protein phosphorylation | -3.67832 |
| GO:0072175 | Biological Processes | epithelial tube formation | -3.56352 |
| GO:0003179 | Biological Processes | heart valve morphogenesis | -3.52918 |
| GO:0072132 | Biological Processes | mesenchyme morphogenesis | -3.52918 |
| GO:0060393 | Biological Processes | regulation of pathway-restricted SMAD protein phosphorylation | -3.37542 |
| GO:0021915 | Biological Processes | neural tube development | -3.33278 |
| GO:0003170 | Biological Processes | heart valve development | -3.31504 |
| GO:0060389 | Biological Processes | pathway-restricted SMAD protein phosphorylation | -3.31504 |
| GO:0003205 | Biological Processes | cardiac chamber development | -3.24946 |
| GO:0051301 | Biological Processes | cell division | -3.2414 |
| GO:0003208 | Biological Processes | cardiac ventricle morphogenesis | -3.18486 |
| GO:0031214 | Biological Processes | biomineral tissue development | -3.17052 |
| GO:0110148 | Biological Processes | biomineralization | -3.15142 |
| GO:0003151 | Biological Processes | outflow tract morphogenesis | -3.13311 |
| GO:0060395 | Biological Processes | SMAD protein signal transduction | -3.00514 |
| GO:0001843 | Biological Processes | neural tube closure | -2.91747 |
| GO:0060606 | Biological Processes | tube closure | -2.90348 |
| GO:0070167 | Biological Processes | regulation of biomineral tissue development | -2.80989 |
| GO:0110149 | Biological Processes | regulation of biomineralization | -2.78447 |
| GO:0090100 | Biological Processes | positive regulation of transmembrane receptor protein serine/threonine kinase signaling pathway | -2.60975 |
| GO:0030282 | Biological Processes | bone mineralization | -2.55694 |
| GO:0003231 | Biological Processes | cardiac ventricle development | -2.5164 |
| GO:0030509 | Biological Processes | BMP signaling pathway | -2.26738 |
| GO:0071772 | Biological Processes | response to BMP | -2.16844 |
| GO:0071773 | Biological Processes | cellular response to BMP stimulus | -2.16844 |
| GO:0007517 | Biological Processes | muscle organ development | -2.13589 |
| GO:1904035 | Biological Processes | regulation of epithelial cell apoptotic process | -10.0355 |
| GO:2000351 | Biological Processes | regulation of endothelial cell apoptotic process | -9.90531 |
| GO:0072577 | Biological Processes | endothelial cell apoptotic process | -9.66174 |
| GO:1904019 | Biological Processes | epithelial cell apoptotic process | -9.28257 |
| GO:2000352 | Biological Processes | negative regulation of endothelial cell apoptotic process | -5.81479 |
| GO:1904036 | Biological Processes | negative regulation of epithelial cell apoptotic process | -5.16465 |
| GO:0090303 | Biological Processes | positive regulation of wound healing | -4.94439 |
| GO:0030194 | Biological Processes | positive regulation of blood coagulation | -4.67657 |
| GO:1900048 | Biological Processes | positive regulation of hemostasis | -4.67657 |
| GO:0050820 | Biological Processes | positive regulation of coagulation | -4.61931 |
| GO:1903036 | Biological Processes | positive regulation of response to wounding | -4.60013 |
| GO:0071887 | Biological Processes | leukocyte apoptotic process | -9.86386 |
| GO:2000106 | Biological Processes | regulation of leukocyte apoptotic process | -7.43013 |
| GO:0045165 | Biological Processes | cell fate commitment | -6.7657 |
| GO:0010212 | Biological Processes | response to ionizing radiation | -5.85783 |
| GO:2000378 | Biological Processes | negative regulation of reactive oxygen species metabolic process | -5.16465 |
| GO:0070228 | Biological Processes | regulation of lymphocyte apoptotic process | -5.09867 |
| GO:0010506 | Biological Processes | regulation of autophagy | -4.99787 |
| GO:0001836 | Biological Processes | release of cytochrome c from mitochondria | -4.94439 |
| GO:0007005 | Biological Processes | mitochondrion organization | -4.60729 |
| GO:0070227 | Biological Processes | lymphocyte apoptotic process | -4.60013 |
| GO:0006914 | Biological Processes | autophagy | -4.49427 |
| GO:0061919 | Biological Processes | process utilizing autophagic mechanism | -4.49427 |
| GO:0010507 | Biological Processes | negative regulation of autophagy | -4.27639 |
| GO:0031647 | Biological Processes | regulation of protein stability | -4.13147 |
| GO:0008637 | Biological Processes | apoptotic mitochondrial changes | -3.92645 |
| GO:0016241 | Biological Processes | regulation of macroautophagy | -3.46701 |
| GO:0046902 | Biological Processes | regulation of mitochondrial membrane permeability | -3.35496 |
| GO:0090559 | Biological Processes | regulation of membrane permeability | -3.0835 |
| GO:0007006 | Biological Processes | mitochondrial membrane organization | -2.58833 |
| GO:0031330 | Biological Processes | negative regulation of cellular catabolic process | -2.46204 |
| GO:0016236 | Biological Processes | macroautophagy | -2.29154 |
| GO:0030902 | Biological Processes | hindbrain development | -2.25944 |
| GO:0001659 | Biological Processes | temperature homeostasis | -9.53503 |
| GO:0106106 | Biological Processes | cold-induced thermogenesis | -2.3246 |
| GO:0120161 | Biological Processes | regulation of cold-induced thermogenesis | -2.3246 |
| GO:1990845 | Biological Processes | adaptive thermogenesis | -2.22059 |
| GO:0097305 | Biological Processes | response to alcohol | -9.42657 |
| GO:0045471 | Biological Processes | response to ethanol | -8.91172 |
| GO:0035264 | Biological Processes | multicellular organism growth | -6.16581 |
| GO:0040014 | Biological Processes | regulation of multicellular organism growth | -4.8865 |
| GO:0048678 | Biological Processes | response to axon injury | -4.35659 |
| GO:0042391 | Biological Processes | regulation of membrane potential | -3.26848 |
| GO:0001655 | Biological Processes | urogenital system development | -9.40927 |
| GO:0001822 | Biological Processes | kidney development | -6.36033 |
| GO:0072001 | Biological Processes | renal system development | -6.2615 |
| GO:0007160 | Biological Processes | cell-matrix adhesion | -5.89149 |
| GO:0048008 | Biological Processes | platelet-derived growth factor receptor signaling pathway | -5.03522 |
| GO:0001952 | Biological Processes | regulation of cell-matrix adhesion | -4.89151 |
| GO:0031589 | Biological Processes | cell-substrate adhesion | -4.63779 |
| GO:0010810 | Biological Processes | regulation of cell-substrate adhesion | -3.76375 |
| GO:0072073 | Biological Processes | kidney epithelium development | -3.52662 |
| GO:0048041 | Biological Processes | focal adhesion assembly | -2.94598 |
| GO:0001657 | Biological Processes | ureteric bud development | -2.88964 |
| GO:0072163 | Biological Processes | mesonephric epithelium development | -2.87597 |
| GO:0072164 | Biological Processes | mesonephric tubule development | -2.87597 |
| GO:0007044 | Biological Processes | cell-substrate junction assembly | -2.83588 |
| GO:0001823 | Biological Processes | mesonephros development | -2.82281 |
| GO:0150115 | Biological Processes | cell-substrate junction organization | -2.75959 |
| GO:0072009 | Biological Processes | nephron epithelium development | -2.6651 |
| GO:0072006 | Biological Processes | nephron development | -2.33303 |
| GO:0061419 | Biological Processes | positive regulation of transcription from RNA polymerase II promoter in response to hypoxia | -9.3859 |
| GO:1901522 | Biological Processes | positive regulation of transcription from RNA polymerase II promoter involved in cellular response to chemical stimulus | -8.81046 |
| GO:0061418 | Biological Processes | regulation of transcription from RNA polymerase II promoter in response to hypoxia | -7.71304 |
| GO:0007423 | Biological Processes | sensory organ development | -7.19595 |
| GO:0043620 | Biological Processes | regulation of DNA-templated transcription in response to stress | -6.79746 |
| GO:0048872 | Biological Processes | homeostasis of number of cells | -6.60429 |
| GO:0036003 | Biological Processes | positive regulation of transcription from RNA polymerase II promoter in response to stress | -6.5494 |
| GO:0010039 | Biological Processes | response to iron ion | -6.02607 |
| GO:1902692 | Biological Processes | regulation of neuroblast proliferation | -5.97072 |
| GO:0002262 | Biological Processes | myeloid cell homeostasis | -5.7096 |
| GO:0090596 | Biological Processes | sensory organ morphogenesis | -5.62125 |
| GO:0007589 | Biological Processes | body fluid secretion | -5.52522 |
| GO:0021700 | Biological Processes | developmental maturation | -5.37605 |
| GO:0043618 | Biological Processes | regulation of transcription from RNA polymerase II promoter in response to stress | -5.34205 |
| GO:0007405 | Biological Processes | neuroblast proliferation | -4.97412 |
| GO:0071695 | Biological Processes | anatomical structure maturation | -4.78577 |
| GO:0002052 | Biological Processes | positive regulation of neuroblast proliferation | -4.67657 |
| GO:0001654 | Biological Processes | eye development | -4.57754 |
| GO:0150063 | Biological Processes | visual system development | -4.54795 |
| GO:0048880 | Biological Processes | sensory system development | -4.50421 |
| GO:0001782 | Biological Processes | B cell homeostasis | -4.27788 |
| GO:0001776 | Biological Processes | leukocyte homeostasis | -4.27639 |
| GO:2000177 | Biological Processes | regulation of neural precursor cell proliferation | -4.27639 |
| GO:0001501 | Biological Processes | skeletal system development | -3.73205 |
| GO:0030218 | Biological Processes | erythrocyte differentiation | -3.72063 |
| GO:0034101 | Biological Processes | erythrocyte homeostasis | -3.60133 |
| GO:0046887 | Biological Processes | positive regulation of hormone secretion | -3.56352 |
| GO:2000179 | Biological Processes | positive regulation of neural precursor cell proliferation | -3.55281 |
| GO:0048010 | Biological Processes | vascular endothelial growth factor receptor signaling pathway | -3.43896 |
| GO:0010821 | Biological Processes | regulation of mitochondrion organization | -3.43233 |
| GO:0061351 | Biological Processes | neural precursor cell proliferation | -3.40964 |
| GO:0002260 | Biological Processes | lymphocyte homeostasis | -3.37542 |
| GO:0048592 | Biological Processes | eye morphogenesis | -3.34352 |
| GO:0048469 | Biological Processes | cell maturation | -3.15142 |
| GO:0010822 | Biological Processes | positive regulation of mitochondrion organization | -3.15011 |
| GO:0043010 | Biological Processes | camera-type eye development | -3.01294 |
| GO:0090277 | Biological Processes | positive regulation of peptide hormone secretion | -2.73524 |
| GO:0002793 | Biological Processes | positive regulation of peptide secretion | -2.69964 |
| GO:0050730 | Biological Processes | regulation of peptidyl-tyrosine phosphorylation | -9.23039 |
| GO:0050731 | Biological Processes | positive regulation of peptidyl-tyrosine phosphorylation | -9.13591 |
| GO:0018108 | Biological Processes | peptidyl-tyrosine phosphorylation | -8.93633 |
| GO:0018212 | Biological Processes | peptidyl-tyrosine modification | -8.90021 |
| GO:0031334 | Biological Processes | positive regulation of protein-containing complex assembly | -5.80631 |
| GO:0043254 | Biological Processes | regulation of protein-containing complex assembly | -4.16733 |
| GO:0044089 | Biological Processes | positive regulation of cellular component biogenesis | -3.66007 |
| GO:0007569 | Biological Processes | cell aging | -9.06554 |
| GO:0010660 | Biological Processes | regulation of muscle cell apoptotic process | -8.886 |
| GO:0010657 | Biological Processes | muscle cell apoptotic process | -8.71205 |
| GO:0008630 | Biological Processes | intrinsic apoptotic signaling pathway in response to DNA damage | -6.90626 |
| GO:0071560 | Biological Processes | cellular response to transforming growth factor beta stimulus | -6.84316 |
| GO:0071559 | Biological Processes | response to transforming growth factor beta | -6.7657 |
| GO:0007179 | Biological Processes | transforming growth factor beta receptor signaling pathway | -6.31933 |
| GO:0033028 | Biological Processes | myeloid cell apoptotic process | -6.2676 |
| GO:1902230 | Biological Processes | negative regulation of intrinsic apoptotic signaling pathway in response to DNA damage | -6.20391 |
| GO:1902253 | Biological Processes | regulation of intrinsic apoptotic signaling pathway by p53 class mediator | -6.20391 |
| GO:0034390 | Biological Processes | smooth muscle cell apoptotic process | -6.14253 |
| GO:0034391 | Biological Processes | regulation of smooth muscle cell apoptotic process | -6.14253 |
| GO:1902229 | Biological Processes | regulation of intrinsic apoptotic signaling pathway in response to DNA damage | -5.81479 |
| GO:0042771 | Biological Processes | intrinsic apoptotic signaling pathway in response to DNA damage by p53 class mediator | -5.49895 |
| GO:1902166 | Biological Processes | negative regulation of intrinsic apoptotic signaling pathway in response to DNA damage by p53 class mediator | -5.35713 |
| GO:1902165 | Biological Processes | regulation of intrinsic apoptotic signaling pathway in response to DNA damage by p53 class mediator | -5.1715 |
| GO:0090342 | Biological Processes | regulation of cell aging | -4.8865 |
| GO:2001020 | Biological Processes | regulation of response to DNA damage stimulus | -4.8843 |
| GO:1902254 | Biological Processes | negative regulation of intrinsic apoptotic signaling pathway by p53 class mediator | -4.79948 |
| GO:0007178 | Biological Processes | transmembrane receptor protein serine/threonine kinase signaling pathway | -4.67621 |
| GO:0033032 | Biological Processes | regulation of myeloid cell apoptotic process | -4.61931 |
| GO:2000108 | Biological Processes | positive regulation of leukocyte apoptotic process | -4.512 |
| GO:0072332 | Biological Processes | intrinsic apoptotic signaling pathway by p53 class mediator | -4.50729 |
| GO:0090344 | Biological Processes | negative regulation of cell aging | -4.41309 |
| GO:2001021 | Biological Processes | negative regulation of response to DNA damage stimulus | -4.39824 |
| GO:1901797 | Biological Processes | negative regulation of signal transduction by p53 class mediator | -4.27788 |
| GO:2001252 | Biological Processes | positive regulation of chromosome organization | -4.27547 |
| GO:0031058 | Biological Processes | positive regulation of histone modification | -4.18146 |
| GO:1901796 | Biological Processes | regulation of signal transduction by p53 class mediator | -4.14501 |
| GO:0043516 | Biological Processes | regulation of DNA damage response, signal transduction by p53 class mediator | -4.11761 |
| GO:0090287 | Biological Processes | regulation of cellular response to growth factor stimulus | -4.09983 |
| GO:1905269 | Biological Processes | positive regulation of chromatin organization | -3.94223 |
| GO:0033574 | Biological Processes | response to testosterone | -3.87867 |
| GO:0017015 | Biological Processes | regulation of transforming growth factor beta receptor signaling pathway | -3.61414 |
| GO:1903844 | Biological Processes | regulation of cellular response to transforming growth factor beta stimulus | -3.57602 |
| GO:0090092 | Biological Processes | regulation of transmembrane receptor protein serine/threonine kinase signaling pathway | -3.46135 |
| GO:0070059 | Biological Processes | intrinsic apoptotic signaling pathway in response to endoplasmic reticulum stress | -3.35496 |
| GO:0031056 | Biological Processes | regulation of histone modification | -3.30099 |
| GO:0033044 | Biological Processes | regulation of chromosome organization | -3.29175 |
| GO:0072331 | Biological Processes | signal transduction by p53 class mediator | -3.20947 |
| GO:0030330 | Biological Processes | DNA damage response, signal transduction by p53 class mediator | -3.16736 |
| GO:0051145 | Biological Processes | smooth muscle cell differentiation | -3.16736 |
| GO:0048145 | Biological Processes | regulation of fibroblast proliferation | -3.05154 |
| GO:0030512 | Biological Processes | negative regulation of transforming growth factor beta receptor signaling pathway | -3.03587 |
| GO:0048144 | Biological Processes | fibroblast proliferation | -3.03587 |
| GO:0016575 | Biological Processes | histone deacetylation | -3.00514 |
| GO:1902275 | Biological Processes | regulation of chromatin organization | -2.89109 |
| GO:0006476 | Biological Processes | protein deacetylation | -2.73524 |
| GO:0035601 | Biological Processes | protein deacylation | -2.60975 |
| GO:0098732 | Biological Processes | macromolecule deacylation | -2.56731 |
| GO:0090101 | Biological Processes | negative regulation of transmembrane receptor protein serine/threonine kinase signaling pathway | -2.44875 |
| GO:0016570 | Biological Processes | histone modification | -2.32004 |
| GO:0016569 | Biological Processes | covalent chromatin modification | -2.26647 |
| GO:0042770 | Biological Processes | signal transduction in response to DNA damage | -2.10488 |
| GO:0071897 | Biological Processes | DNA biosynthetic process | -2.03201 |
| GO:0051051 | Biological Processes | negative regulation of transport | -9.02178 |
| GO:0034762 | Biological Processes | regulation of transmembrane transport | -9.01165 |
| GO:0032409 | Biological Processes | regulation of transporter activity | -8.55791 |
| GO:0043269 | Biological Processes | regulation of ion transport | -7.16968 |
| GO:0043271 | Biological Processes | negative regulation of ion transport | -6.9881 |
| GO:0010959 | Biological Processes | regulation of metal ion transport | -6.35511 |
| GO:0051926 | Biological Processes | negative regulation of calcium ion transport | -6.21712 |
| GO:0034763 | Biological Processes | negative regulation of transmembrane transport | -5.94437 |
| GO:0051924 | Biological Processes | regulation of calcium ion transport | -5.64369 |
| GO:0022898 | Biological Processes | regulation of transmembrane transporter activity | -5.39649 |
| GO:0032412 | Biological Processes | regulation of ion transmembrane transporter activity | -4.41171 |
| GO:0032413 | Biological Processes | negative regulation of ion transmembrane transporter activity | -4.27639 |
| GO:0006816 | Biological Processes | calcium ion transport | -4.23107 |
| GO:1904063 | Biological Processes | negative regulation of cation transmembrane transport | -3.99055 |
| GO:0032410 | Biological Processes | negative regulation of transporter activity | -3.88002 |
| GO:0034766 | Biological Processes | negative regulation of ion transmembrane transport | -3.88002 |
| GO:1904062 | Biological Processes | regulation of cation transmembrane transport | -3.71979 |
| GO:2001257 | Biological Processes | regulation of cation channel activity | -3.03291 |
| GO:0034765 | Biological Processes | regulation of ion transmembrane transport | -2.9902 |
| GO:0019915 | Biological Processes | lipid storage | -8.85037 |
| GO:0010888 | Biological Processes | negative regulation of lipid storage | -8.70479 |
| GO:0010883 | Biological Processes | regulation of lipid storage | -8.36259 |
| GO:0051235 | Biological Processes | maintenance of location | -7.17019 |
| GO:0050680 | Biological Processes | negative regulation of epithelial cell proliferation | -6.91527 |
| GO:1905953 | Biological Processes | negative regulation of lipid localization | -6.31429 |
| GO:0043281 | Biological Processes | regulation of cysteine-type endopeptidase activity involved in apoptotic process | -6.17823 |
| GO:0052548 | Biological Processes | regulation of endopeptidase activity | -6.10593 |
| GO:0045598 | Biological Processes | regulation of fat cell differentiation | -6.03416 |
| GO:0052547 | Biological Processes | regulation of peptidase activity | -5.87449 |
| GO:2000116 | Biological Processes | regulation of cysteine-type endopeptidase activity | -5.84248 |
| GO:0045862 | Biological Processes | positive regulation of proteolysis | -5.56188 |
| GO:0010633 | Biological Processes | negative regulation of epithelial cell migration | -5.17391 |
| GO:0030730 | Biological Processes | sequestering of triglyceride | -5.00946 |
| GO:0045444 | Biological Processes | fat cell differentiation | -4.77508 |
| GO:2000272 | Biological Processes | negative regulation of signaling receptor activity | -4.7501 |
| GO:0010950 | Biological Processes | positive regulation of endopeptidase activity | -4.15916 |
| GO:0010952 | Biological Processes | positive regulation of peptidase activity | -3.96625 |
| GO:0043280 | Biological Processes | positive regulation of cysteine-type endopeptidase activity involved in apoptotic process | -3.57602 |
| GO:0016525 | Biological Processes | negative regulation of angiogenesis | -3.37622 |
| GO:2000181 | Biological Processes | negative regulation of blood vessel morphogenesis | -3.35434 |
| GO:2001056 | Biological Processes | positive regulation of cysteine-type endopeptidase activity | -3.35434 |
| GO:1901343 | Biological Processes | negative regulation of vasculature development | -3.34352 |
| GO:0001937 | Biological Processes | negative regulation of endothelial cell proliferation | -3.09981 |
| GO:0043537 | Biological Processes | negative regulation of blood vessel endothelial cell migration | -3.0835 |
| GO:0006919 | Biological Processes | activation of cysteine-type endopeptidase activity involved in apoptotic process | -3.02041 |
| GO:0010596 | Biological Processes | negative regulation of endothelial cell migration | -2.80989 |
| GO:0050830 | Biological Processes | defense response to Gram-positive bacterium | -2.72325 |
| GO:0001649 | Biological Processes | osteoblast differentiation | -2.67729 |
| GO:0045667 | Biological Processes | regulation of osteoblast differentiation | -2.43015 |
| GO:0044321 | Biological Processes | response to leptin | -8.60412 |
| GO:0044320 | Biological Processes | cellular response to leptin stimulus | -6.98356 |
| GO:0033210 | Biological Processes | leptin-mediated signaling pathway | -5.69856 |
| GO:0030258 | Biological Processes | lipid modification | -3.84785 |
| GO:0019932 | Biological Processes | second-messenger-mediated signaling | -8.53115 |
| GO:0019722 | Biological Processes | calcium-mediated signaling | -6.31933 |
| GO:0060193 | Biological Processes | positive regulation of lipase activity | -6.21712 |
| GO:0060191 | Biological Processes | regulation of lipase activity | -5.59409 |
| GO:0010518 | Biological Processes | positive regulation of phospholipase activity | -5.03522 |
| GO:0010517 | Biological Processes | regulation of phospholipase activity | -4.69857 |
| GO:0010863 | Biological Processes | positive regulation of phospholipase C activity | -3.9424 |
| GO:1900274 | Biological Processes | regulation of phospholipase C activity | -3.84799 |
| GO:0016032 | Biological Processes | viral process | -8.47853 |
| GO:0019058 | Biological Processes | viral life cycle | -6.10366 |
| GO:0044403 | Biological Processes | biological process involved in symbiotic interaction | -5.23732 |
| GO:0051701 | Biological Processes | biological process involved in interaction with host | -5.07073 |
| GO:0044409 | Biological Processes | entry into host | -4.53164 |
| GO:0052126 | Biological Processes | movement in host environment | -4.22809 |
| GO:0046718 | Biological Processes | viral entry into host cell | -3.42094 |
| GO:0007265 | Biological Processes | Ras protein signal transduction | -2.08644 |
| GO:0035265 | Biological Processes | organ growth | -8.03881 |
| GO:0014855 | Biological Processes | striated muscle cell proliferation | -4.50729 |
| GO:0014031 | Biological Processes | mesenchymal cell development | -4.27639 |
| GO:0055017 | Biological Processes | cardiac muscle tissue growth | -4.10937 |
| GO:0010719 | Biological Processes | negative regulation of epithelial to mesenchymal transition | -4.00957 |
| GO:0060419 | Biological Processes | heart growth | -3.97428 |
| GO:0046620 | Biological Processes | regulation of organ growth | -3.94223 |
| GO:0043583 | Biological Processes | ear development | -3.83831 |
| GO:0007009 | Biological Processes | plasma membrane organization | -3.4438 |
| GO:0060038 | Biological Processes | cardiac muscle cell proliferation | -3.41741 |
| GO:0055021 | Biological Processes | regulation of cardiac muscle tissue growth | -3.18486 |
| GO:0060420 | Biological Processes | regulation of heart growth | -3.06741 |
| GO:0014032 | Biological Processes | neural crest cell development | -3.02041 |
| GO:0048864 | Biological Processes | stem cell development | -2.94598 |
| GO:0006885 | Biological Processes | regulation of pH | -2.88964 |
| GO:0014033 | Biological Processes | neural crest cell differentiation | -2.87597 |
| GO:0042471 | Biological Processes | ear morphogenesis | -2.59899 |
| GO:0048562 | Biological Processes | embryonic organ morphogenesis | -2.31762 |
| GO:0010256 | Biological Processes | endomembrane system organization | -2.07299 |
| GO:0048565 | Biological Processes | digestive tract development | -7.69779 |
| GO:0055123 | Biological Processes | digestive system development | -7.46993 |
| GO:0042303 | Biological Processes | molting cycle | -6.70502 |
| GO:0042633 | Biological Processes | hair cycle | -6.70502 |
| GO:0008544 | Biological Processes | epidermis development | -6.04279 |
| GO:0001942 | Biological Processes | hair follicle development | -5.86863 |
| GO:0022404 | Biological Processes | molting cycle process | -5.79002 |
| GO:0022405 | Biological Processes | hair cycle process | -5.79002 |
| GO:0098773 | Biological Processes | skin epidermis development | -5.76448 |
| GO:0043588 | Biological Processes | skin development | -5.55506 |
| GO:0006959 | Biological Processes | humoral immune response | -5.00668 |
| GO:0048566 | Biological Processes | embryonic digestive tract development | -4.27788 |
| GO:0019079 | Biological Processes | viral genome replication | -3.58863 |
| GO:0045069 | Biological Processes | regulation of viral genome replication | -2.97519 |
| GO:1903900 | Biological Processes | regulation of viral life cycle | -2.29154 |
| GO:0050792 | Biological Processes | regulation of viral process | -2.16844 |
| GO:0046688 | Biological Processes | response to copper ion | -7.31691 |
| GO:0001975 | Biological Processes | response to amphetamine | -4.1952 |
| GO:0043279 | Biological Processes | response to alkaloid | -3.80556 |
| GO:0014075 | Biological Processes | response to amine | -3.67832 |
| GO:0050954 | Biological Processes | sensory perception of mechanical stimulus | -3.11394 |
| GO:0007605 | Biological Processes | sensory perception of sound | -2.24373 |
| GO:0045981 | Biological Processes | positive regulation of nucleotide metabolic process | -7.26401 |
| GO:1900544 | Biological Processes | positive regulation of purine nucleotide metabolic process | -7.26401 |
| GO:0007263 | Biological Processes | nitric oxide mediated signal transduction | -6.33379 |
| GO:1900542 | Biological Processes | regulation of purine nucleotide metabolic process | -5.79002 |
| GO:0006140 | Biological Processes | regulation of nucleotide metabolic process | -5.73925 |
| GO:0044282 | Biological Processes | small molecule catabolic process | -5.54487 |
| GO:0045776 | Biological Processes | negative regulation of blood pressure | -5.49895 |
| GO:0030810 | Biological Processes | positive regulation of nucleotide biosynthetic process | -4.61931 |
| GO:1900373 | Biological Processes | positive regulation of purine nucleotide biosynthetic process | -4.61931 |
| GO:0072521 | Biological Processes | purine-containing compound metabolic process | -4.27013 |
| GO:0055086 | Biological Processes | nucleobase-containing small molecule metabolic process | -4.26018 |
| GO:0045454 | Biological Processes | cell redox homeostasis | -4.15581 |
| GO:0034405 | Biological Processes | response to fluid shear stress | -4.11761 |
| GO:1900371 | Biological Processes | regulation of purine nucleotide biosynthetic process | -3.91013 |
| GO:0030808 | Biological Processes | regulation of nucleotide biosynthetic process | -3.87867 |
| GO:0009117 | Biological Processes | nucleotide metabolic process | -3.82804 |
| GO:0006753 | Biological Processes | nucleoside phosphate metabolic process | -3.78488 |
| GO:0045747 | Biological Processes | positive regulation of Notch signaling pathway | -3.73233 |
| GO:0051339 | Biological Processes | regulation of lyase activity | -3.62657 |
| GO:0031279 | Biological Processes | regulation of cyclase activity | -3.57691 |
| GO:0090407 | Biological Processes | organophosphate biosynthetic process | -3.5274 |
| GO:0009165 | Biological Processes | nucleotide biosynthetic process | -3.48471 |
| GO:0006163 | Biological Processes | purine nucleotide metabolic process | -3.47284 |
| GO:1901293 | Biological Processes | nucleoside phosphate biosynthetic process | -3.4691 |
| GO:2000736 | Biological Processes | regulation of stem cell differentiation | -3.43896 |
| GO:0009064 | Biological Processes | glutamine family amino acid metabolic process | -3.06741 |
| GO:0006164 | Biological Processes | purine nucleotide biosynthetic process | -2.97284 |
| GO:0072522 | Biological Processes | purine-containing compound biosynthetic process | -2.89906 |
| GO:0008593 | Biological Processes | regulation of Notch signaling pathway | -2.83588 |
| GO:0046395 | Biological Processes | carboxylic acid catabolic process | -2.63017 |
| GO:0016054 | Biological Processes | organic acid catabolic process | -2.60394 |
| GO:0007219 | Biological Processes | Notch signaling pathway | -2.11176 |
| GO:0034284 | Biological Processes | response to monosaccharide | -7.23269 |
| GO:0009743 | Biological Processes | response to carbohydrate | -6.84316 |
| GO:0009746 | Biological Processes | response to hexose | -4.8843 |
| GO:0009749 | Biological Processes | response to glucose | -3.83831 |
| GO:0001678 | Biological Processes | cellular glucose homeostasis | -3.13256 |
| GO:0071333 | Biological Processes | cellular response to glucose stimulus | -2.25944 |
| GO:0071331 | Biological Processes | cellular response to hexose stimulus | -2.24373 |
| GO:0071326 | Biological Processes | cellular response to monosaccharide stimulus | -2.23597 |
| GO:0071322 | Biological Processes | cellular response to carbohydrate stimulus | -2.16844 |
| GO:0055067 | Biological Processes | monovalent inorganic cation homeostasis | -7.17893 |
| GO:0055080 | Biological Processes | cation homeostasis | -6.93257 |
| GO:0098771 | Biological Processes | inorganic ion homeostasis | -6.8439 |
| GO:0055078 | Biological Processes | sodium ion homeostasis | -6.6756 |
| GO:0003014 | Biological Processes | renal system process | -6.63362 |
| GO:0050801 | Biological Processes | ion homeostasis | -6.55953 |
| GO:0055065 | Biological Processes | metal ion homeostasis | -6.45046 |
| GO:0030003 | Biological Processes | cellular cation homeostasis | -5.57824 |
| GO:0006873 | Biological Processes | cellular ion homeostasis | -5.47884 |
| GO:0006875 | Biological Processes | cellular metal ion homeostasis | -5.07599 |
| GO:0035813 | Biological Processes | regulation of renal sodium excretion | -4.79948 |
| GO:0035812 | Biological Processes | renal sodium excretion | -4.67657 |
| GO:0044062 | Biological Processes | regulation of excretion | -4.61931 |
| GO:0051482 | Biological Processes | positive regulation of cytosolic calcium ion concentration involved in phospholipase C-activating G protein-coupled signaling pathway | -4.15581 |
| GO:0098801 | Biological Processes | regulation of renal system process | -4.11761 |
| GO:0007200 | Biological Processes | phospholipase C-activating G protein-coupled receptor signaling pathway | -3.97428 |
| GO:0072507 | Biological Processes | divalent inorganic cation homeostasis | -3.73205 |
| GO:0007588 | Biological Processes | excretion | -3.37542 |
| GO:0006874 | Biological Processes | cellular calcium ion homeostasis | -3.19129 |
| GO:0055074 | Biological Processes | calcium ion homeostasis | -3.13166 |
| GO:0007204 | Biological Processes | positive regulation of cytosolic calcium ion concentration | -3.03705 |
| GO:0072503 | Biological Processes | cellular divalent inorganic cation homeostasis | -3.00847 |
| GO:0051480 | Biological Processes | regulation of cytosolic calcium ion concentration | -2.83687 |
| GO:0050778 | Biological Processes | positive regulation of immune response | -7.03346 |
| GO:0030278 | Biological Processes | regulation of ossification | -5.09947 |
| GO:0046824 | Biological Processes | positive regulation of nucleocytoplasmic transport | -4.8583 |
| GO:0006606 | Biological Processes | protein import into nucleus | -4.50444 |
| GO:0051170 | Biological Processes | import into nucleus | -4.45116 |
| GO:0006913 | Biological Processes | nucleocytoplasmic transport | -4.13147 |
| GO:0051169 | Biological Processes | nuclear transport | -4.13147 |
| GO:0046822 | Biological Processes | regulation of nucleocytoplasmic transport | -3.94223 |
| GO:0017038 | Biological Processes | protein import | -3.916 |
| GO:0042307 | Biological Processes | positive regulation of protein import into nucleus | -3.91013 |
| GO:1904591 | Biological Processes | positive regulation of protein import | -3.84799 |
| GO:0048538 | Biological Processes | thymus development | -3.78881 |
| GO:0045912 | Biological Processes | negative regulation of carbohydrate metabolic process | -3.70503 |
| GO:0042306 | Biological Processes | regulation of protein import into nucleus | -3.41741 |
| GO:1904589 | Biological Processes | regulation of protein import | -3.35496 |
| GO:0090316 | Biological Processes | positive regulation of intracellular protein transport | -3.25963 |
| GO:0034504 | Biological Processes | protein localization to nucleus | -3.22958 |
| GO:1900182 | Biological Processes | positive regulation of protein localization to nucleus | -2.94598 |
| GO:0032388 | Biological Processes | positive regulation of intracellular transport | -2.88316 |
| GO:0030004 | Biological Processes | cellular monovalent inorganic cation homeostasis | -2.73524 |
| GO:0002253 | Biological Processes | activation of immune response | -2.72233 |
| GO:0033157 | Biological Processes | regulation of intracellular protein transport | -2.68415 |
| GO:0018107 | Biological Processes | peptidyl-threonine phosphorylation | -2.57777 |
| GO:0072594 | Biological Processes | establishment of protein localization to organelle | -2.51056 |
| GO:0018210 | Biological Processes | peptidyl-threonine modification | -2.48691 |
| GO:1900180 | Biological Processes | regulation of protein localization to nucleus | -2.39385 |
| GO:0032386 | Biological Processes | regulation of intracellular transport | -2.09086 |
| GO:0043112 | Biological Processes | receptor metabolic process | -6.87956 |
| GO:0048260 | Biological Processes | positive regulation of receptor-mediated endocytosis | -6.79746 |
| GO:0031623 | Biological Processes | receptor internalization | -6.54158 |
| GO:0002092 | Biological Processes | positive regulation of receptor internalization | -6.33379 |
| GO:0006898 | Biological Processes | receptor-mediated endocytosis | -5.71217 |
| GO:0006897 | Biological Processes | endocytosis | -5.66235 |
| GO:0045807 | Biological Processes | positive regulation of endocytosis | -5.39413 |
| GO:0048259 | Biological Processes | regulation of receptor-mediated endocytosis | -5.13635 |
| GO:0002090 | Biological Processes | regulation of receptor internalization | -5.00439 |
| GO:0030100 | Biological Processes | regulation of endocytosis | -4.95244 |
| GO:0032147 | Biological Processes | activation of protein kinase activity | -3.38728 |
| GO:0019233 | Biological Processes | sensory perception of pain | -6.80357 |
| GO:0001890 | Biological Processes | placenta development | -5.92681 |
| GO:0001892 | Biological Processes | embryonic placenta development | -3.00514 |
| GO:0009266 | Biological Processes | response to temperature stimulus | -6.67442 |
| GO:0009408 | Biological Processes | response to heat | -6.61028 |
| GO:0034605 | Biological Processes | cellular response to heat | -3.22064 |
| GO:0007276 | Biological Processes | gamete generation | -2.83181 |
| GO:0007292 | Biological Processes | female gamete generation | -2.35868 |
| GO:1990776 | Biological Processes | response to angiotensin | -6.2676 |
| GO:1904385 | Biological Processes | cellular response to angiotensin | -4.56452 |
| GO:0043467 | Biological Processes | regulation of generation of precursor metabolites and energy | -6.20479 |
| GO:0043470 | Biological Processes | regulation of carbohydrate catabolic process | -5.03522 |
| GO:1903578 | Biological Processes | regulation of ATP metabolic process | -4.27639 |
| GO:1903580 | Biological Processes | positive regulation of ATP metabolic process | -4.00957 |
| GO:0015980 | Biological Processes | energy derivation by oxidation of organic compounds | -3.99264 |
| GO:0006091 | Biological Processes | generation of precursor metabolites and energy | -3.82804 |
| GO:0006110 | Biological Processes | regulation of glycolytic process | -3.78881 |
| GO:0016052 | Biological Processes | carbohydrate catabolic process | -3.32211 |
| GO:0046034 | Biological Processes | ATP metabolic process | -3.31297 |
| GO:0006096 | Biological Processes | glycolytic process | -3.03587 |
| GO:0006757 | Biological Processes | ATP generation from ADP | -3.02041 |
| GO:0046031 | Biological Processes | ADP metabolic process | -2.90348 |
| GO:0006165 | Biological Processes | nucleoside diphosphate phosphorylation | -2.78447 |
| GO:0046939 | Biological Processes | nucleotide phosphorylation | -2.75959 |
| GO:0009135 | Biological Processes | purine nucleoside diphosphate metabolic process | -2.73524 |
| GO:0009179 | Biological Processes | purine ribonucleoside diphosphate metabolic process | -2.73524 |
| GO:0006090 | Biological Processes | pyruvate metabolic process | -2.69964 |
| GO:0009185 | Biological Processes | ribonucleoside diphosphate metabolic process | -2.69964 |
| GO:0035821 | Biological Processes | modulation of process of other organism | -2.6765 |
| GO:0009132 | Biological Processes | nucleoside diphosphate metabolic process | -2.50648 |
| GO:0034368 | Biological Processes | protein-lipid complex remodeling | -6.0833 |
| GO:0034369 | Biological Processes | plasma lipoprotein particle remodeling | -6.0833 |
| GO:0034367 | Biological Processes | protein-containing complex remodeling | -5.97072 |
| GO:0071827 | Biological Processes | plasma lipoprotein particle organization | -5.34205 |
| GO:0071825 | Biological Processes | protein-lipid complex subunit organization | -5.19865 |
| GO:0097006 | Biological Processes | regulation of plasma lipoprotein particle levels | -4.29607 |
| GO:0010661 | Biological Processes | positive regulation of muscle cell apoptotic process | -5.91712 |
| GO:1904706 | Biological Processes | negative regulation of vascular associated smooth muscle cell proliferation | -5.49895 |
| GO:0048662 | Biological Processes | negative regulation of smooth muscle cell proliferation | -4.53 |
| GO:0010043 | Biological Processes | response to zinc ion | -3.4609 |
| GO:0097306 | Biological Processes | cellular response to alcohol | -2.87597 |
| GO:0097530 | Biological Processes | granulocyte migration | -5.85783 |
| GO:0070098 | Biological Processes | chemokine-mediated signaling pathway | -5.6897 |
| GO:1990868 | Biological Processes | response to chemokine | -5.48057 |
| GO:1990869 | Biological Processes | cellular response to chemokine | -5.48057 |
| GO:1990266 | Biological Processes | neutrophil migration | -4.97553 |
| GO:0071621 | Biological Processes | granulocyte chemotaxis | -4.94149 |
| GO:0051767 | Biological Processes | nitric-oxide synthase biosynthetic process | -4.79948 |
| GO:0051769 | Biological Processes | regulation of nitric-oxide synthase biosynthetic process | -4.79948 |
| GO:0030593 | Biological Processes | neutrophil chemotaxis | -3.99055 |
| GO:0043507 | Biological Processes | positive regulation of JUN kinase activity | -3.87867 |
| GO:0071622 | Biological Processes | regulation of granulocyte chemotaxis | -3.62657 |
| GO:0043506 | Biological Processes | regulation of JUN kinase activity | -3.4609 |
| GO:0008277 | Biological Processes | regulation of G protein-coupled receptor signaling pathway | -2.40281 |
| GO:0032570 | Biological Processes | response to progesterone | -5.76587 |
| GO:1902894 | Biological Processes | negative regulation of pri-miRNA transcription by RNA polymerase II | -5.08791 |
| GO:0032740 | Biological Processes | positive regulation of interleukin-17 production | -4.67657 |
| GO:0032620 | Biological Processes | interleukin-17 production | -3.81805 |
| GO:0032660 | Biological Processes | regulation of interleukin-17 production | -3.81805 |
| GO:0010712 | Biological Processes | regulation of collagen metabolic process | -3.73233 |
| GO:0045685 | Biological Processes | regulation of glial cell differentiation | -3.11634 |
| GO:0014854 | Biological Processes | response to inactivity | -5.69856 |
| GO:0009308 | Biological Processes | amine metabolic process | -5.04541 |
| GO:0016311 | Biological Processes | dephosphorylation | -4.24402 |
| GO:0035303 | Biological Processes | regulation of dephosphorylation | -3.62706 |
| GO:0006470 | Biological Processes | protein dephosphorylation | -3.24321 |
| GO:0010921 | Biological Processes | regulation of phosphatase activity | -2.99007 |
| GO:0035304 | Biological Processes | regulation of protein dephosphorylation | -2.90348 |
| GO:0050807 | Biological Processes | regulation of synapse organization | -2.81371 |
| GO:0050803 | Biological Processes | regulation of synapse structure or activity | -2.76192 |
| GO:0018958 | Biological Processes | phenol-containing compound metabolic process | -2.65381 |
| GO:0044106 | Biological Processes | cellular amine metabolic process | -2.60975 |
| GO:0050808 | Biological Processes | synapse organization | -2.48868 |
| GO:0009615 | Biological Processes | response to virus | -5.62221 |
| GO:1903706 | Biological Processes | regulation of hemopoiesis | -4.59246 |
| GO:0032088 | Biological Processes | negative regulation of NF-kappaB transcription factor activity | -4.16313 |
| GO:0043433 | Biological Processes | negative regulation of DNA-binding transcription factor activity | -4.12561 |
| GO:0032615 | Biological Processes | interleukin-12 production | -3.37542 |
| GO:0032655 | Biological Processes | regulation of interleukin-12 production | -3.37542 |
| GO:1902105 | Biological Processes | regulation of leukocyte differentiation | -3.28473 |
| GO:0051607 | Biological Processes | defense response to virus | -2.44457 |
| GO:0140546 | Biological Processes | defense response to symbiont | -2.44457 |
| GO:0045619 | Biological Processes | regulation of lymphocyte differentiation | -2.09805 |
| GO:0043123 | Biological Processes | positive regulation of I-kappaB kinase/NF-kappaB signaling | -2.01928 |
| GO:0007162 | Biological Processes | negative regulation of cell adhesion | -5.15189 |
| GO:0022408 | Biological Processes | negative regulation of cell-cell adhesion | -4.00744 |
| GO:0032945 | Biological Processes | negative regulation of mononuclear cell proliferation | -2.99007 |
| GO:0070664 | Biological Processes | negative regulation of leukocyte proliferation | -2.90348 |
| GO:0050866 | Biological Processes | negative regulation of cell activation | -2.82126 |
| GO:1903038 | Biological Processes | negative regulation of leukocyte cell-cell adhesion | -2.35007 |
| GO:0002695 | Biological Processes | negative regulation of leukocyte activation | -2.01298 |
| GO:0019748 | Biological Processes | secondary metabolic process | -4.8865 |
| GO:0016101 | Biological Processes | diterpenoid metabolic process | -4.27639 |
| GO:0006721 | Biological Processes | terpenoid metabolic process | -4.09184 |
| GO:0006720 | Biological Processes | isoprenoid metabolic process | -3.79108 |
| GO:0042572 | Biological Processes | retinol metabolic process | -3.65217 |
| GO:0019369 | Biological Processes | arachidonic acid metabolic process | -3.43896 |
| GO:0001523 | Biological Processes | retinoid metabolic process | -3.03587 |
| GO:0006766 | Biological Processes | vitamin metabolic process | -2.69964 |
| GO:0001676 | Biological Processes | long-chain fatty acid metabolic process | -2.63157 |
| GO:0038083 | Biological Processes | peptidyl-tyrosine autophosphorylation | -4.79948 |
| GO:0046777 | Biological Processes | protein autophosphorylation | -2.69798 |
| GO:0070498 | Biological Processes | interleukin-1-mediated signaling pathway | -4.32136 |
| GO:1904037 | Biological Processes | positive regulation of epithelial cell apoptotic process | -3.87867 |
| GO:0002720 | Biological Processes | positive regulation of cytokine production involved in immune response | -3.29556 |
| GO:0002456 | Biological Processes | T cell mediated immunity | -2.64264 |
| GO:0035270 | Biological Processes | endocrine system development | -2.47725 |
| GO:0051099 | Biological Processes | positive regulation of binding | -4.26351 |
| GO:0038127 | Biological Processes | ERBB signaling pathway | -3.70691 |
| GO:0051098 | Biological Processes | regulation of binding | -3.67438 |
| GO:0043388 | Biological Processes | positive regulation of DNA binding | -3.50599 |
| GO:0007173 | Biological Processes | epidermal growth factor receptor signaling pathway | -2.6651 |
| GO:0051101 | Biological Processes | regulation of DNA binding | -2.56731 |
| GO:0002221 | Biological Processes | pattern recognition receptor signaling pathway | -4.22809 |
| GO:0002764 | Biological Processes | immune response-regulating signaling pathway | -3.92819 |
| GO:0002224 | Biological Processes | toll-like receptor signaling pathway | -3.67984 |
| GO:0034121 | Biological Processes | regulation of toll-like receptor signaling pathway | -3.11634 |
| GO:0062207 | Biological Processes | regulation of pattern recognition receptor signaling pathway | -2.69964 |
| GO:0033198 | Biological Processes | response to ATP | -4.1952 |
| GO:0006939 | Biological Processes | smooth muscle contraction | -3.88002 |
| GO:0060047 | Biological Processes | heart contraction | -3.58129 |
| GO:0046683 | Biological Processes | response to organophosphorus | -3.57602 |
| GO:0003015 | Biological Processes | heart process | -3.50045 |
| GO:0014074 | Biological Processes | response to purine-containing compound | -3.37622 |
| GO:0006940 | Biological Processes | regulation of smooth muscle contraction | -3.33483 |
| GO:0003012 | Biological Processes | muscle system process | -3.17621 |
| GO:0090257 | Biological Processes | regulation of muscle system process | -2.53425 |
| GO:0006937 | Biological Processes | regulation of muscle contraction | -2.13267 |
| GO:0006936 | Biological Processes | muscle contraction | -2.04736 |
| GO:0031341 | Biological Processes | regulation of cell killing | -4.00699 |
| GO:0001906 | Biological Processes | cell killing | -2.96446 |
| GO:0048246 | Biological Processes | macrophage chemotaxis | -3.9424 |
| GO:0002686 | Biological Processes | negative regulation of leukocyte migration | -3.73233 |
| GO:1905517 | Biological Processes | macrophage migration | -3.48324 |
| GO:0072678 | Biological Processes | T cell migration | -3.29556 |
| GO:0072676 | Biological Processes | lymphocyte migration | -2.57777 |
| GO:0008360 | Biological Processes | regulation of cell shape | -2.24373 |
| GO:0043087 | Biological Processes | regulation of GTPase activity | -2.03884 |
| GO:0101023 | Biological Processes | vascular endothelial cell proliferation | -3.67832 |
| GO:1905562 | Biological Processes | regulation of vascular endothelial cell proliferation | -3.67832 |
| GO:0015909 | Biological Processes | long-chain fatty acid transport | -3.31504 |
| GO:0035924 | Biological Processes | cellular response to vascular endothelial growth factor stimulus | -3.16736 |
| GO:0045121 | Cellular Components | membrane raft | -10.7079 |
| GO:0098857 | Cellular Components | membrane microdomain | -10.7079 |
| GO:0005901 | Cellular Components | caveola | -7.30374 |
| GO:0044853 | Cellular Components | plasma membrane raft | -6.47476 |
| GO:0031983 | Cellular Components | vesicle lumen | -8.33614 |
| GO:0034774 | Cellular Components | secretory granule lumen | -7.20451 |
| GO:0060205 | Cellular Components | cytoplasmic vesicle lumen | -7.17019 |
| GO:0031091 | Cellular Components | platelet alpha granule | -5.61758 |
| GO:0031093 | Cellular Components | platelet alpha granule lumen | -4.72413 |
| GO:0031012 | Cellular Components | extracellular matrix | -7.07921 |
| GO:0030312 | Cellular Components | external encapsulating structure | -7.07155 |
| GO:0062023 | Cellular Components | collagen-containing extracellular matrix | -6.18125 |
| GO:0009897 | Cellular Components | external side of plasma membrane | -6.27573 |
| GO:0098552 | Cellular Components | side of membrane | -5.69905 |
| GO:0043235 | Cellular Components | receptor complex | -4.48845 |
| GO:0098797 | Cellular Components | plasma membrane protein complex | -2.93769 |
| GO:0098802 | Cellular Components | plasma membrane signaling receptor complex | -2.23592 |
| GO:0031256 | Cellular Components | leading edge membrane | -2.05139 |
| GO:0031253 | Cellular Components | cell projection membrane | -2.04736 |
| GO:0000323 | Cellular Components | lytic vacuole | -4.3076 |
| GO:0005764 | Cellular Components | lysosome | -4.3076 |
| GO:0035578 | Cellular Components | azurophil granule lumen | -2.88964 |
| GO:0005766 | Cellular Components | primary lysosome | -2.22059 |
| GO:0042582 | Cellular Components | azurophil granule | -2.22059 |
| GO:0005775 | Cellular Components | vacuolar lumen | -2.09805 |
| GO:0043025 | Cellular Components | neuronal cell body | -3.73727 |
| GO:0044297 | Cellular Components | cell body | -3.39317 |
| GO:0030424 | Cellular Components | axon | -3.08794 |
| GO:0120111 | Cellular Components | neuron projection cytoplasm | -2.86245 |
| GO:0030425 | Cellular Components | dendrite | -2.414 |
| GO:0097447 | Cellular Components | dendritic tree | -2.40741 |
| GO:0043209 | Cellular Components | myelin sheath | -3.70503 |
| GO:0098685 | Cellular Components | Schaffer collateral - CA1 synapse | -3.0835 |
| GO:0098978 | Cellular Components | glutamatergic synapse | -2.92544 |
| GO:0043197 | Cellular Components | dendritic spine | -2.03201 |
| GO:0044309 | Cellular Components | neuron spine | -2.02563 |
| GO:1904813 | Cellular Components | ficolin-1-rich granule lumen | -3.67984 |
| GO:0005925 | Cellular Components | focal adhesion | -3.33792 |
| GO:0030055 | Cellular Components | cell-substrate junction | -3.30023 |
| GO:0101002 | Cellular Components | ficolin-1-rich granule | -3.02418 |
| GO:0005667 | Cellular Components | transcription regulator complex | -3.38189 |
| GO:0090575 | Cellular Components | RNA polymerase II transcription regulator complex | -3.18016 |
| GO:0030139 | Cellular Components | endocytic vesicle | -2.93685 |
| GO:0045177 | Cellular Components | apical part of cell | -2.4416 |
| GO:0016605 | Cellular Components | PML body | -2.72325 |
| GO:0019866 | Cellular Components | organelle inner membrane | -2.7199 |
| GO:0005740 | Cellular Components | mitochondrial envelope | -2.61696 |
| GO:0005777 | Cellular Components | peroxisome | -2.31624 |
| GO:0042579 | Cellular Components | microbody | -2.31624 |
| GO:0005635 | Cellular Components | nuclear envelope | -2.31615 |
| GO:0031965 | Cellular Components | nuclear membrane | -2.28125 |
| GO:0005788 | Cellular Components | endoplasmic reticulum lumen | -2.20172 |
| GO:0005126 | Molecular Functions | cytokine receptor binding | -16.1806 |
| GO:0005125 | Molecular Functions | cytokine activity | -14.0254 |
| GO:0030545 | Molecular Functions | signaling receptor regulator activity | -11.7809 |
| GO:0048018 | Molecular Functions | receptor ligand activity | -11.2297 |
| GO:0030546 | Molecular Functions | signaling receptor activator activity | -11.1352 |
| GO:0008083 | Molecular Functions | growth factor activity | -5.64658 |
| GO:0004879 | Molecular Functions | nuclear receptor activity | -10.4627 |
| GO:0098531 | Molecular Functions | ligand-activated transcription factor activity | -10.4627 |
| GO:0140297 | Molecular Functions | DNA-binding transcription factor binding | -8.7241 |
| GO:0061629 | Molecular Functions | RNA polymerase II-specific DNA-binding transcription factor binding | -8.70849 |
| GO:0019904 | Molecular Functions | protein domain specific binding | -7.11581 |
| GO:0008134 | Molecular Functions | transcription factor binding | -6.98076 |
| GO:0005496 | Molecular Functions | steroid binding | -6.88018 |
| GO:0001223 | Molecular Functions | transcription coactivator binding | -6.02607 |
| GO:0001228 | Molecular Functions | DNA-binding transcription activator activity, RNA polymerase II-specific | -6.00012 |
| GO:0001216 | Molecular Functions | DNA-binding transcription activator activity | -5.95244 |
| GO:0001221 | Molecular Functions | transcription coregulator binding | -5.95038 |
| GO:0016922 | Molecular Functions | nuclear receptor binding | -5.94437 |
| GO:0140296 | Molecular Functions | general transcription initiation factor binding | -5.30502 |
| GO:0003682 | Molecular Functions | chromatin binding | -5.13623 |
| GO:0001091 | Molecular Functions | RNA polymerase II general transcription initiation factor binding | -4.8657 |
| GO:0001098 | Molecular Functions | basal transcription machinery binding | -3.22064 |
| GO:0001099 | Molecular Functions | basal RNA polymerase II transcription machinery binding | -3.22064 |
| GO:0051117 | Molecular Functions | ATPase binding | -2.91747 |
| GO:0020037 | Molecular Functions | heme binding | -10.4086 |
| GO:0046906 | Molecular Functions | tetrapyrrole binding | -10.1371 |
| GO:0004497 | Molecular Functions | monooxygenase activity | -8.33282 |
| GO:0016705 | Molecular Functions | oxidoreductase activity, acting on paired donors, with incorporation or reduction of molecular oxygen | -7.98197 |
| GO:0016491 | Molecular Functions | oxidoreductase activity | -7.8091 |
| GO:0008395 | Molecular Functions | steroid hydroxylase activity | -5.71833 |
| GO:0019825 | Molecular Functions | oxygen binding | -3.97554 |
| GO:0005506 | Molecular Functions | iron ion binding | -3.35434 |
| GO:0002020 | Molecular Functions | protease binding | -8.98772 |
| GO:1990782 | Molecular Functions | protein tyrosine kinase binding | -3.97428 |
| GO:0019901 | Molecular Functions | protein kinase binding | -8.28705 |
| GO:0019900 | Molecular Functions | kinase binding | -7.70709 |
| GO:0002039 | Molecular Functions | p53 binding | -3.29556 |
| GO:0031625 | Molecular Functions | ubiquitin protein ligase binding | -3.17611 |
| GO:0044389 | Molecular Functions | ubiquitin-like protein ligase binding | -3.05537 |
| GO:0046982 | Molecular Functions | protein heterodimerization activity | -2.98327 |
| GO:0042826 | Molecular Functions | histone deacetylase binding | -2.47725 |
| GO:0070851 | Molecular Functions | growth factor receptor binding | -7.38368 |
| GO:0005149 | Molecular Functions | interleukin-1 receptor binding | -5.08791 |
| GO:0042562 | Molecular Functions | hormone binding | -7.27313 |
| GO:0008236 | Molecular Functions | serine-type peptidase activity | -6.34844 |
| GO:0017171 | Molecular Functions | serine hydrolase activity | -6.29052 |
| GO:0004175 | Molecular Functions | endopeptidase activity | -6.04041 |
| GO:0004252 | Molecular Functions | serine-type endopeptidase activity | -5.3409 |
| GO:0008233 | Molecular Functions | peptidase activity | -4.76458 |
| GO:0008237 | Molecular Functions | metallopeptidase activity | -3.99706 |
| GO:0004222 | Molecular Functions | metalloendopeptidase activity | -3.82017 |
| GO:0042803 | Molecular Functions | protein homodimerization activity | -6.31602 |
| GO:0005178 | Molecular Functions | integrin binding | -5.94437 |
| GO:0001618 | Molecular Functions | virus receptor activity | -3.0835 |
| GO:0140272 | Molecular Functions | exogenous protein binding | -3.06741 |
| GO:0050839 | Molecular Functions | cell adhesion molecule binding | -2.76363 |
| GO:0016209 | Molecular Functions | antioxidant activity | -5.71432 |
| GO:0004601 | Molecular Functions | peroxidase activity | -3.62657 |
| GO:0016684 | Molecular Functions | oxidoreductase activity, acting on peroxide as acceptor | -3.52918 |
| GO:0016709 | Molecular Functions | oxidoreductase activity, acting on paired donors, with incorporation or reduction of molecular oxygen, NAD(P)H as one donor, and incorporation of one atom of oxygen | -5.49895 |
| GO:0031406 | Molecular Functions | carboxylic acid binding | -5.30032 |
| GO:0050661 | Molecular Functions | NADP binding | -3.57691 |
| GO:0043177 | Molecular Functions | organic acid binding | -3.43233 |
| GO:0033293 | Molecular Functions | monocarboxylic acid binding | -3.03587 |
| GO:0001664 | Molecular Functions | G protein-coupled receptor binding | -5.21809 |
| GO:0042379 | Molecular Functions | chemokine receptor binding | -4.60013 |
| GO:0008009 | Molecular Functions | chemokine activity | -3.67832 |
| GO:0019902 | Molecular Functions | phosphatase binding | -5.16997 |
| GO:0019903 | Molecular Functions | protein phosphatase binding | -4.55923 |
| GO:0005507 | Molecular Functions | copper ion binding | -3.39624 |
| GO:0042277 | Molecular Functions | peptide binding | -2.96571 |
| GO:0033218 | Molecular Functions | amide binding | -2.55073 |
| GO:0051219 | Molecular Functions | phosphoprotein binding | -2.93164 |
