# Supplementary material for: A network pharmacology study on mechanism of resveratrol in treating preeclampsia via regulation of AGE-RAGE and HIF-1 signalling pathways
Source: Front Endocrinol (Lausanne). 2023 Jan 5;13:1044775. doi: 10.3389/fendo.2022.1044775 (PMC9849370; doi:10.3389/fendo.2022.1044775)
Supplement: Supplementary file 5 [file Table_5.docx]

**Additional file 5. The Results of KEGG Pathway Enrichment Analysis**

| **GO** | **Category** | **Description** | **LogP** |
| --- | --- | --- | --- |
| hsa04933 | KEGG Pathway | AGE-RAGE signaling pathway in diabetic complications | -39.2807 |
| hsa05418 | KEGG Pathway | Fluid shear stress and atherosclerosis | -25.75 |
| hsa05200 | KEGG Pathway | Pathways in cancer | -27.2152 |
| hsa05205 | KEGG Pathway | Proteoglycans in cancer | -19.2369 |
| hsa05206 | KEGG Pathway | MicroRNAs in cancer | -13.9358 |
| hsa05144 | KEGG Pathway | Malaria | -22.6093 |
| hsa05142 | KEGG Pathway | Chagas disease (American trypanosomiasis) | -20.449 |
| hsa04668 | KEGG Pathway | TNF signaling pathway | -18.1278 |
| hsa05323 | KEGG Pathway | Rheumatoid arthritis | -17.1232 |
| hsa05140 | KEGG Pathway | Leishmania infection | -16.81 |
| hsa05133 | KEGG Pathway | Pertussis | -16.2838 |
| hsa04657 | KEGG Pathway | IL-17 signaling pathway | -15.4858 |
| hsa05152 | KEGG Pathway | Tuberculosis | -15.1672 |
| hsa05145 | KEGG Pathway | Toxoplasmosis | -14.4993 |
| hsa05321 | KEGG Pathway | Inflammatory bowel disease (IBD) | -13.333 |
| hsa04064 | KEGG Pathway | NF-kappa B signaling pathway | -13.1422 |
| hsa05146 | KEGG Pathway | Amoebiasis | -13.1422 |
| hsa05143 | KEGG Pathway | African trypanosomiasis | -13.012 |
| hsa04060 | KEGG Pathway | Cytokine-cytokine receptor interaction | -11.8329 |
| hsa05162 | KEGG Pathway | Measles | -9.90878 |
| hsa04010 | KEGG Pathway | MAPK signaling pathway | -9.3766 |
| hsa05164 | KEGG Pathway | Influenza A | -9.3616 |
| hsa04380 | KEGG Pathway | Osteoclast differentiation | -8.91172 |
| hsa05132 | KEGG Pathway | Salmonella infection | -8.58004 |
| hsa04620 | KEGG Pathway | Toll-like receptor signaling pathway | -8.30336 |
| hsa05134 | KEGG Pathway | Legionellosis | -8.27017 |
| hsa04621 | KEGG Pathway | NOD-like receptor signaling pathway | -8.19551 |
| hsa05168 | KEGG Pathway | Herpes simplex infection | -7.90772 |
| hsa05020 | KEGG Pathway | Prion diseases | -7.72979 |
| hsa05166 | KEGG Pathway | HTLV-I infection | -6.81713 |
| hsa01523 | KEGG Pathway | Antifolate resistance | -6.0833 |
| hsa05332 | KEGG Pathway | Graft-versus-host disease | -5.58329 |
| hsa04640 | KEGG Pathway | Hematopoietic cell lineage | -4.09184 |
| hsa04940 | KEGG Pathway | Type I diabetes mellitus | -3.84799 |
| hsa04623 | KEGG Pathway | Cytosolic DNA-sensing pathway | -3.35496 |
| hsa04066 | KEGG Pathway | HIF-1 signaling pathway | -19.7712 |
| hsa05219 | KEGG Pathway | Bladder cancer | -13.278 |
| hsa01522 | KEGG Pathway | Endocrine resistance | -10.1795 |
| hsa04151 | KEGG Pathway | PI3K-Akt signaling pathway | -10.1056 |
| hsa01521 | KEGG Pathway | EGFR tyrosine kinase inhibitor resistance | -9.14844 |
| hsa05215 | KEGG Pathway | Prostate cancer | -8.85037 |
| hsa04915 | KEGG Pathway | Estrogen signaling pathway | -8.74098 |
| hsa04015 | KEGG Pathway | Rap1 signaling pathway | -5.96672 |
| hsa05230 | KEGG Pathway | Central carbon metabolism in cancer | -5.95038 |
| hsa05224 | KEGG Pathway | Breast cancer | -5.64658 |
| hsa05213 | KEGG Pathway | Endometrial cancer | -5.23335 |
| hsa04510 | KEGG Pathway | Focal adhesion | -5.13225 |
| hsa05223 | KEGG Pathway | Non-small cell lung cancer | -4.97412 |
| hsa05214 | KEGG Pathway | Glioma | -4.80331 |
| hsa05218 | KEGG Pathway | Melanoma | -4.6734 |
| hsa04014 | KEGG Pathway | Ras signaling pathway | -4.48541 |
| hsa04072 | KEGG Pathway | Phospholipase D signaling pathway | -4.37389 |
| hsa05216 | KEGG Pathway | Thyroid cancer | -4.36639 |
| hsa04919 | KEGG Pathway | thyroid hormone signaling pathway | -3.69332 |
| hsa04520 | KEGG Pathway | Adherens junction | -3.18486 |
| hsa04540 | KEGG Pathway | Gap junction | -2.93164 |
| hsa04912 | KEGG Pathway | GnRH signaling pathway | -2.87597 |
| hsa05231 | KEGG Pathway | Choline metabolism in cancer | -2.68801 |
| hsa04068 | KEGG Pathway | foxo signaling pathway | -17.1055 |
| hsa04211 | KEGG Pathway | Longevity regulating pathway | -13.0598 |
| hsa04213 | KEGG Pathway | Longevity regulating pathway - multiple species | -8.13763 |
| hsa04930 | KEGG Pathway | Type II diabetes mellitus | -5.37991 |
| hsa04152 | KEGG Pathway | AMPK signaling pathway | -4.87512 |
| hsa04150 | KEGG Pathway | mTOR signaling pathway | -4.54538 |
| hsa04022 | KEGG Pathway | cGMP-PKG signaling pathway | -4.26351 |
| hsa04960 | KEGG Pathway | Aldosterone-regulated sodium reabsorption | -4.04456 |
| hsa04923 | KEGG Pathway | Regulation of lipolysis in adipocytes | -3.4609 |
| hsa04910 | KEGG Pathway | Insulin signaling pathway | -2.37613 |
| hsa04932 | KEGG Pathway | Non-alcoholic fatty liver disease (NAFLD) | -15.8952 |
| hsa04931 | KEGG Pathway | insulin resistance | -11.2245 |
| hsa04920 | KEGG Pathway | Adipocytokine signaling pathway | -6.21712 |
| hsa05161 | KEGG Pathway | Hepatitis B | -15.6306 |
| hsa05160 | KEGG Pathway | Hepatitis C | -9.64777 |
| hsa04917 | KEGG Pathway | Prolactin signaling pathway | -7.59775 |
| hsa04062 | KEGG Pathway | Chemokine signaling pathway | -5.35458 |
| hsa05120 | KEGG Pathway | Epithelial cell signaling in Helicobacter pylori infection | -4.69857 |
| hsa05131 | KEGG Pathway | Shigellosis | -4.69857 |
| hsa04660 | KEGG Pathway | T cell receptor signaling pathway | -3.99055 |
| hsa05203 | KEGG Pathway | Viral carcinogenesis | -3.85743 |
| hsa05221 | KEGG Pathway | Acute myeloid leukemia | -3.52918 |
| hsa04622 | KEGG Pathway | RIG-I-like receptor signaling pathway | -3.22064 |
| hsa04024 | KEGG Pathway | cAMP signaling pathway | -2.70494 |
| hsa05212 | KEGG Pathway | Pancreatic cancer | -11.6305 |
| hsa04659 | KEGG Pathway | Th17 cell differentiation | -8.07792 |
| hsa05410 | KEGG Pathway | Hypertrophic cardiomyopathy (HCM) | -7.36613 |
| hsa05211 | KEGG Pathway | Renal cell carcinoma | -6.34772 |
| hsa05220 | KEGG Pathway | Chronic myeloid leukemia | -6.15479 |
| hsa04350 | KEGG Pathway | TGF-beta signaling pathway | -4.33617 |
| hsa05414 | KEGG Pathway | Dilated cardiomyopathy | -4.21876 |
| hsa04110 | KEGG Pathway | Cell cycle | -2.50648 |
| hsa04390 | KEGG Pathway | Hippo signaling pathway | -2.09805 |
| hsa05202 | KEGG Pathway | Transcriptional misregulation in cancer | -8.9235 |
| hsa05222 | KEGG Pathway | Small cell lung cancer | -8.9586 |
| hsa04137 | KEGG Pathway | Mitophagy - animal | -3.27639 |
| hsa05014 | KEGG Pathway | Amyotrophic lateral sclerosis (ALS) | -8.66209 |
| hsa04115 | KEGG Pathway | p53 signaling pathway | -4.6734 |
| hsa05016 | KEGG Pathway | Huntington's disease | -4.03894 |
| hsa05210 | KEGG Pathway | Colorectal cancer | -8.22521 |
| hsa04071 | KEGG Pathway | Sphingolipid signaling pathway | -7.67417 |
| hsa04210 | KEGG Pathway | Apoptosis | -6.05253 |
| hsa04722 | KEGG Pathway | Neurotrophin signaling pathway | -5.04541 |
| hsa04140 | KEGG Pathway | Regulation of autophagy | -4.82676 |
| hsa01524 | KEGG Pathway | Platinum drug resistance | -4.57641 |
| hsa04650 | KEGG Pathway | Natural killer cell mediated cytotoxicity | -3.56352 |
| hsa05010 | KEGG Pathway | Alzheimer's disease | -3.15142 |
| hsa04726 | KEGG Pathway | Serotonergic synapse | -2.55694 |
| hsa04261 | KEGG Pathway | Adrenergic signaling in cardiomyocytes | -2.29971 |
| hsa04670 | KEGG Pathway | Leukocyte transendothelial migration | -8.02422 |
| hsa04514 | KEGG Pathway | Cell adhesion molecules (CAMs) | -4.64441 |
| hsa05169 | KEGG Pathway | Epstein-Barr virus infection | -7.47968 |
| hsa04672 | KEGG Pathway | Intestinal immune network for IgA production | -5.2688 |
| hsa04630 | KEGG Pathway | Jak-STAT signaling pathway | -3.30099 |
| hsa04913 | KEGG Pathway | Ovarian steroidogenesis | -6.71544 |
| hsa_M00109 | KEGG Pathway | C21-Steroid hormone biosynthesis, progesterone => cortisol/cortisone | -6.61137 |
| hsa00140 | KEGG Pathway | Steroid hormone biosynthesis | -6.59815 |
| hsa04371 | KEGG Pathway | Apelin signaling pathway | -5.90938 |
| hsa04020 | KEGG Pathway | Calcium signaling pathway | -3.05054 |
| hsa04370 | KEGG Pathway | VEGF signaling pathway | -4.94439 |
| hsa04921 | KEGG Pathway | Oxytocin signaling pathway | -3.24946 |
| hsa04611 | KEGG Pathway | Platelet activation | -2.44875 |
| hsa04360 | KEGG Pathway | Axon guidance | -2.09126 |
| hsa04610 | KEGG Pathway | Complement and coagulation cascades | -4.44098 |
| hsa04146 | KEGG Pathway | Peroxisome | -4.35659 |
| hsa03320 | KEGG Pathway | PPAR signaling pathway | -3.18486 |
| hsa04924 | KEGG Pathway | Renin secretion | -3.16736 |
