# Supplementary material for: A network pharmacology study on mechanism of resveratrol in treating preeclampsia via regulation of AGE-RAGE and HIF-1 signalling pathways
Source: Front Endocrinol (Lausanne). 2023 Jan 5;13:1044775. doi: 10.3389/fendo.2022.1044775 (PMC9849370; doi:10.3389/fendo.2022.1044775)
Supplement: Supplementary file 6 [file Table_6.docx]

**Additional file 6. The Results of Topological Analysis of PPI Network**

| **Name** | **Betweenness Centrality** | **Closeness Centrality** | **Degree** |
| --- | --- | --- | --- |
| IL6 | 0.045019 | 0.890411 | 57 |
| TNF | 0.045019 | 0.890411 | 57 |
| IL1B | 0.022519 | 0.833333 | 52 |
| VEGFA | 0.018504 | 0.822785 | 51 |
| CCL2 | 0.017872 | 0.8125 | 50 |
| PTGS2 | 0.020252 | 0.792683 | 48 |
| MMP9 | 0.011268 | 0.792683 | 48 |
| TP53 | 0.014381 | 0.77381 | 47 |
| EGFR | 0.030875 | 0.783133 | 47 |
| CXCL8 | 0.01106 | 0.783133 | 47 |
| STAT3 | 0.017441 | 0.77381 | 47 |
| PPARG | 0.027049 | 0.77381 | 46 |
| CASP3 | 0.012985 | 0.77381 | 46 |
| HIF1A | 0.011319 | 0.747126 | 45 |
| IL10 | 0.006529 | 0.747126 | 44 |
| NOS3 | 0.022691 | 0.755814 | 44 |
| ESR1 | 0.03815 | 0.747126 | 43 |
| ICAM1 | 0.006064 | 0.730337 | 42 |
| SERPINE1 | 0.009567 | 0.730337 | 41 |
| CRP | 0.033678 | 0.730337 | 41 |
| MMP2 | 0.004775 | 0.722222 | 40 |
| CAT | 0.006737 | 0.722222 | 40 |
| VCAM1 | 0.005577 | 0.714286 | 40 |
| EDN1 | 0.017287 | 0.714286 | 40 |
| ACE | 0.017224 | 0.698925 | 37 |
| SIRT1 | 0.011249 | 0.691489 | 37 |
| TGFB1 | 0.002938 | 0.698925 | 37 |
| PECAM1 | 0.002512 | 0.691489 | 37 |
| IL1A | 0.003595 | 0.670103 | 35 |
| CXCL12 | 0.002458 | 0.663265 | 34 |
| SELE | 0.001677 | 0.656566 | 33 |
| PTEN | 0.003948 | 0.656566 | 33 |
| MPO | 0.014038 | 0.65 | 32 |
| RELA | 0.00493 | 0.65 | 32 |
| MMP1 | 0.001329 | 0.65 | 31 |
| ITGB1 | 8.69E-04 | 0.619048 | 27 |
| NOS2 | 0.001073 | 0.619048 | 27 |
| IRS1 | 0.003253 | 0.619048 | 26 |
| MAPK1 | 0.004865 | 0.619048 | 26 |
| NFE2L2 | 0.001274 | 0.619048 | 26 |
| AGTR1 | 0.028419 | 0.619048 | 25 |
| SOD2 | 0.001637 | 0.601852 | 25 |
| PLAU | 7.05E-04 | 0.601852 | 25 |
| F3 | 4.23E-04 | 0.59633 | 24 |
| TGFB2 | 3.05E-04 | 0.580357 | 23 |
| AR | 0.008646 | 0.59633 | 22 |
| SOD1 | 0.001856 | 0.575221 | 20 |
| PLAT | 1.51E-04 | 0.565217 | 19 |
| ESR2 | 0.005106 | 0.580357 | 19 |
| MIF | 3.48E-04 | 0.570175 | 19 |
| TLR9 | 1.83E-04 | 0.560345 | 18 |
| CYP1A1 | 0.007129 | 0.565217 | 17 |
| PON1 | 3.62E-04 | 0.53719 | 12 |
| ADIPOR1 | 1.28E-04 | 0.532787 | 10 |
| NR1H4 | 5.99E-04 | 0.528455 | 10 |
| INSR | 4.70E-04 | 0.524194 | 10 |
| SHBG | 0.002746 | 0.532787 | 9 |
| AKR1C3 | 0.004675 | 0.511811 | 8 |
| CYP17A1 | 0.004537 | 0.474453 | 8 |
| BCL2 | 6.54E-05 | 0.492424 | 7 |
| CYP11B2 | 0.002468 | 0.481481 | 6 |
| NR1H3 | 0 | 0.5 | 6 |
| TTR | 8.01E-05 | 0.5 | 5 |
| CYP11B1 | 3.97E-04 | 0.408805 | 4 |
| PAPPA | 0 | 0.433333 | 2 |
| DRD2 | 0 | 0.454545 | 2 |
