# Supplementary material for: A network pharmacology study on mechanism of resveratrol in treating preeclampsia via regulation of AGE-RAGE and HIF-1 signalling pathways
Source: Front Endocrinol (Lausanne). 2023 Jan 5;13:1044775. doi: 10.3389/fendo.2022.1044775 (PMC9849370; doi:10.3389/fendo.2022.1044775)
Supplement: Supplementary file 7 [file Table_7.docx]

**Additional file 7. The Results of Topological Analysis of TP Network**

| **Name** | **Type** | **Betweenness Centrality** | **Closeness Centrality** | **Degree** |
| --- | --- | --- | --- | --- |
| IL6 | Targets | 0.069019 | 0.512658 | 21 |
| RELA | Targets | 0.090466 | 0.525974 | 21 |
| TNF | Targets | 0.052211 | 0.487952 | 20 |
| MAPK1 | Targets | 0.056182 | 0.482143 | 18 |
| IL1B | Targets | 0.033048 | 0.455056 | 17 |
| TGFB1 | Targets | 0.034852 | 0.482143 | 16 |
| TGFB2 | Targets | 0.034457 | 0.47093 | 16 |
| CASP3 | Targets | 0.019575 | 0.445055 | 13 |
| CXCL8 | Targets | 0.023047 | 0.445055 | 13 |
| BCL2 | Targets | 0.028652 | 0.45 | 12 |
| STAT3 | Targets | 0.021107 | 0.445055 | 12 |
| VEGFA | Targets | 0.02062 | 0.435484 | 11 |
| TP53 | Targets | 0.036508 | 0.45 | 11 |
| IL10 | Targets | 0.01247 | 0.389423 | 11 |
| IL1A | Targets | 0.011679 | 0.413265 | 10 |
| EGFR | Targets | 0.018371 | 0.417526 | 10 |
| MMP9 | Targets | 0.015985 | 0.413265 | 9 |
| CCL2 | Targets | 0.007446 | 0.389423 | 8 |
| NOS2 | Targets | 0.005969 | 0.389423 | 8 |
| ICAM1 | Targets | 0.005669 | 0.378505 | 7 |
| PTGS2 | Targets | 0.006972 | 0.397059 | 7 |
| MMP2 | Targets | 0.009712 | 0.40099 | 6 |
| VCAM1 | Targets | 0.004193 | 0.37156 | 6 |
| ITGB1 | Targets | 0.003387 | 0.37156 | 6 |
| PTEN | Targets | 0.004941 | 0.375 | 6 |
| INSR | Targets | 0.008307 | 0.375 | 6 |
| IRS1 | Targets | 0.007162 | 0.364865 | 6 |
| NOS3 | Targets | 0.004665 | 0.378505 | 5 |
| SELE | Targets | 0.002682 | 0.361607 | 5 |
| TLR9 | Targets | 0.001898 | 0.3375 | 5 |
| EDN1 | Targets | 0.002009 | 0.364865 | 4 |
| MMP1 | Targets | 0.001541 | 0.352174 | 4 |
| CXCL12 | Targets | 0.001724 | 0.361607 | 4 |
| SERPINE1 | Targets | 9.75E-04 | 0.352174 | 3 |
| HIF1A | Targets | 0.001066 | 0.358407 | 3 |
| PLAU | Targets | 0.001125 | 0.34322 | 3 |
| SIRT1 | Targets | 0.001769 | 0.334711 | 3 |
| AGTR1 | Targets | 4.96E-04 | 0.364865 | 2 |
| F3 | Targets | 2.10E-04 | 0.340336 | 2 |
| PPARG | Targets | 0.002261 | 0.352174 | 2 |
| PECAM1 | Targets | 3.55E-04 | 0.331967 | 2 |
| ESR1 | Targets | 4.34E-04 | 0.316406 | 2 |
| CAT | Targets | 5.02E-04 | 0.316406 | 2 |
| SOD2 | Targets | 5.02E-04 | 0.316406 | 2 |
| NR1H3 | Targets | 1.86E-04 | 0.306818 | 2 |
| ADIPOR1 | Targets | 8.79E-04 | 0.318898 | 2 |
| AR | Targets | 0 | 0.329268 | 1 |
| NFE2L2 | Targets | 0 | 0.311538 | 1 |
| PLAT | Targets | 0 | 0.311538 | 1 |
| ACE | Targets | 0 | 0.304511 | 1 |
| SOD1 | Targets | 0 | 0.287234 | 1 |
| ESR2 | Targets | 0 | 0.27551 | 1 |
| Pathways in cancer | Pathways | 0.122974 | 0.487952 | 24 |
| AGE-RAGE signaling pathway in diabetic complications | Pathways | 0.093046 | 0.482143 | 23 |
| Fluid shear stress and atherosclerosis | Pathways | 0.096394 | 0.45 | 18 |
| Proteoglycans in cancer | Pathways | 0.051445 | 0.445055 | 16 |
| Chagas disease (American trypanosomiasis) | Pathways | 0.046102 | 0.435484 | 14 |
| HIF-1 signaling pathway | Pathways | 0.038894 | 0.435484 | 14 |
| MicroRNAs in cancer | Pathways | 0.034988 | 0.430851 | 14 |
| Malaria | Pathways | 0.027098 | 0.413265 | 13 |
| TNF signaling pathway | Pathways | 0.02337 | 0.430851 | 13 |
| foxo signaling pathway | Pathways | 0.056211 | 0.413265 | 13 |
| Non-alcoholic fatty liver disease (NAFLD) | Pathways | 0.049139 | 0.421875 | 13 |
| Hepatitis B | Pathways | 0.015603 | 0.430851 | 13 |
| Tuberculosis | Pathways | 0.017068 | 0.430851 | 13 |
| Rheumatoid arthritis | Pathways | 0.017365 | 0.409091 | 12 |
| Cytokine-cytokine receptor interaction | Pathways | 0.016156 | 0.417526 | 12 |
| PI3K-Akt signaling pathway | Pathways | 0.021943 | 0.426316 | 12 |
| Leishmania infection | Pathways | 0.011973 | 0.421875 | 11 |
| Pertussis | Pathways | 0.010868 | 0.421875 | 11 |
| IL-17 signaling pathway | Pathways | 0.014122 | 0.421875 | 11 |
| Toxoplasmosis | Pathways | 0.010446 | 0.421875 | 11 |
| NF-kappa B signaling pathway | Pathways | 0.017642 | 0.413265 | 10 |
| Amoebiasis | Pathways | 0.006374 | 0.409091 | 10 |
| Longevity regulating pathway | Pathways | 0.055453 | 0.40099 | 10 |
| Inflammatory bowel disease (IBD) | Pathways | 0.00523 | 0.40099 | 9 |
| insulin resistance | Pathways | 0.019457 | 0.40099 | 9 |
| Measles | Pathways | 0.01059 | 0.413265 | 9 |
| Bladder cancer | Pathways | 0.007048 | 0.397059 | 8 |
| African trypanosomiasis | Pathways | 0.007448 | 0.382075 | 8 |
| Pancreatic cancer | Pathways | 0.00469 | 0.405 | 8 |
| Endocrine resistance | Pathways | 0.033147 | 0.378505 | 8 |
